# Genetic Dissection of Quantitative Trait Loci for Hemostasis and Thrombosis on Mouse Chromosomes 11 and 5 Using Congenic and Subcongenic Strains

**DOI:** 10.1371/journal.pone.0077539

**Published:** 2013-10-17

**Authors:** Jane Hoover-Plow, Qila Sa, Menggui Huang, Jessica Grondolsky

**Affiliations:** Department of Cardiovascular Medicine and Department of Molecular Cardiology, Joseph J. Jacobs Center for Thrombosis and Vascular Biology, Clinic, Lerner Research Institute, Cleveland, Ohio, United States of America; University of Leuven, Belgium

## Abstract

Susceptibility to thrombosis varies in human populations as well as many inbred mouse strains. Only a small portion of this variation has been identified, suggesting that there are unknown modifier genes. The objective of this study was to narrow the quantitative trait locus (QTL) intervals previously identified for hemostasis and thrombosis on mouse distal chromosome 11 (*Hmtb6*) and on chromosome 5 (*Hmtb4* and *Hmtb5*). In a tail bleeding/rebleeding assay, a reporter assay for hemostasis and thrombosis, subcongenic strain (6A-2) had longer clot stability time than did C57BL/6J (B6) mice but a similar time to the B6-Chr11^A/J^ consomic mice, confirming the *Hmtb*6 phenotype. Six congenic and subcongenic strains were constructed for chromosome 5, and the congenic strain, 2A-1, containing the shortest A/J interval (16.6 cM, 26.6 Mbp) in the *Hmtb4* region, had prolonged clot stability time compared to B6 mice. In the 3A-2 and CSS-5 mice bleeding time was shorter than for B6, mice confirming the *Hmtb5* QTL. An increase in bleeding time was identified in another congenic strain (3A-1) with A/J interval (24.8 cM, 32.9 Mbp) in the proximal region of chromosome 5, confirming a QTL for bleeding previously mapped to that region and designated as *Hmtb10*. The subcongenic strain 4A-2 with the A/J fragment in the proximal region had a long occlusion time of the carotid artery after ferric chloride injury and reduced dilation after injury to the abdominal aorta compared to B6 mice, suggesting an additional locus in the proximal region, which was designated *Hmtb11* (5 cM, 21.4 Mbp). CSS-17 mice crossed with congenic strains, 3A-1 and 3A-2, modified tail bleeding. Using congenic and subcongenic analysis, candidate genes previously identified and novel genes were identified as modifiers of hemostasis and thrombosis in each of the loci *Hmtb*6, *Hmtb*4, *Hmtb*10, and *Hmtb*11.

## Introduction

Cardiac and peripheral vascular diseases are the leading cause of death, with thrombosis as the precipitating event. Numerous studies [1-6] show that inherited risk factors contribute significantly to the development of acute myocardial infarction, ischemic stroke, atherosclerosis, thromboembolism and peripheral arterial occlusion. Progress has been made in diagnosis and treating these diseases, but only few of the genetic factors has been identified that explain thrombosis variation. 

Approaches to identify genetic determinants of phenotypic traits have advanced rapidly in the last 15 years. Previously, identification relied on single gene spontaneous mutations that caused an overt phenotype [7-9]. The development of strategies to delete or insert human genes into mice led to the association of specific gene products with specific phenotypes [10-12]. Results of some of these deletions or insertions were surprising; in some cases, there was no obvious phenotype where one was anticipated, and in others, unanticipated phenotypes arose. In addition, murine strain backgrounds were shown to modify the phenotypes of expressed genes [13,14]. With the realization that most common chronic diseases in humans, such as atherosclerosis, obesity or diabetes, are multigenic with more than one gene and even unanticipated gene interactions contributing to disease, new approaches for gene identification and characterization were needed. The development of statistical analyses packages to map complex traits in mice has provided an approach to such complex diseases [15]. These approaches allow for the mapping of loci of genes that contribute quantitatively to a particular phenotype or pathology, a quantitative trait locus (QTL) [16]. The loci of a number of genes for obesity [17-21] and atherosclerosis [22-28] have been successfully mapped by such QTL analyses. The first step in QTL analysis is the identification of two phenotypically disparate strains, performing the strain intercross, and phenotyping and genotyping F2 progeny. Both inbred strains and recombinant congenic strains (strains with essentially the same background except for selective differential chromosomal segments) [29], have been used in such QTL mapping. Only a few studies [14,30] have investigated risk factors for thrombotic disease using mouse genetic approaches.

An approach that we have utilized is to analyze QTL with the chromosome substitution strains (CSS) [31]. In consomic strains the differential variation is a whole chromosome rather than just a chromosomal segment. This approach has been applied to the B6 and A/J mice, which have been characterized to have differences in many traits [32,33]. A panel of 21 CSS was constructed by a “marker-assisted” breeding program. Each of the 21 chromosomes were substituted individually with an A/J chromosome [34] into the B6 background. Often many more QTL are identified with the CSS than with genome-wide scans. In a previous study [35], we found that the two inbred strains, C57BL/6J (B6) and A/J, had marked differences in hemostatic and thrombotic responses. In a tail bleeding/rebleeding assay, rebleeding time (clot stability) was longer in A/J mice compared to B6 mice. In addition, A/J mice had a rapid occlusion time after FeCI_3_ injury of the carotid and reduced patency. The tail bleeding/rebleeding assay, a reporter of hemostasis and thrombosis, was used as screening assay for a panel of consomic strains (B6-Chr1-19, X, Y^A/J^), which had one chromosome from A/J mice in a B6 background [35]. The B6-Chr11^A/J^ (CSS-11) and B6-Chr5^A/J^ (CSS-5) had a phenotype similar to A/J mice, long clot stability. We identified the QTLs, *Hmtb6*, *Hmtb*4, and *Hmtb*5, for hemostasis and thrombosis on mouse chromosomes 11 and 5 in an F_2_ intercross from CSS-11 X B6 and CSS-5 X B6 [36]. The goal of this study was to fine map the QTLs, *Hmtb6*, *Hmtb4*, and *Hmtb*5, using congenic and subcongenic mouse strains. Here we report confirmation of the QTLs for clot stability in *Hmtb6* and *Hmtb4* and the QTL for bleeding in *Hmtb5*. In addition, another QTL for bleeding, *Hmtb10*, and a QTL for carotid occlusion time after injury, *Hmtb11*, were identified in the proximal region. 

## Materials and Methods

### Mice

The parental inbred mouse strains C57BL/6J (B6, 000664) and A/J (000646), and the C57BL/6J-Chr 5A/J/NaJ (CSS-5, 004383) and C57BL/6J-Chr 11A/J/NaJ (CSS-11, 004389) mouse strains were purchased from the Jackson Laboratory (Bar Harbor, ME) and maintained in the Biological Resources Unit facility at the Cleveland Clinic. Congenic and subcongenic strains were generated from CSS-5 and CSS-11 consomic mice using marker-assisted backcrossing [31]. Mice were housed in sterilized isolator cages with a 14hr/10hr light/dark cycle and ad libitum access to water and food. The bleeding/rebleeding assay was performed on both male and female mice at 6-9 wk. of age. The Cleveland Clinic Institutional Animal Care and Use Committee approved this study and procedures were followed in accordance with institutional guidelines. 

### Genotyping

Genomic DNA was prepared from ear punches of the mice and genotyping performed using polymerase chain reaction (PCR) for microsatellite markers (Mouse Mappairs, Invitrogen, Carlsbad, CA). PCR was performed using HotstarTaq Master Mix Kit (QIAGEN, Valencia, CA). The PCR products were detected by electrophoresis on 10% polyacrylamide gels (National Diagnostics, Atlanta, GA) and visualized by ethidium bromide staining. Microsatellite markers and primers for the RLF markers were used to identify mice by marker assisted selection [31,34] (Tables S1 and S2). Marker and genomic coordinates of genes were determined from the Mouse Genome Database (MGD), 2012 [37]. The following terms were used for the annotated analysis: hemostasis, thrombosis, clotting, platelets, fibrinolysis, bleeding, blood and coagulations. Genes were subjected to functional annotation programs, David Bioinformatics Resources 6.7 (http://david.abcc.ncifcrf.gov/home.jsp) [38] and the MGD, 2012 [37].

### Tail Bleeding/Clot Stability Assay

Phenotyping was performed using the bleeding/rebleeding assay as previously described [35]. The mice were anesthetized with ketamine/xylazine (90 mg/kg, 10 mg/kg), the tail prewarmed for 5 minutes in 10 mL of saline at 37°C in a water bath. The tail was lifted from the saline and a 5 mm tail segment amputated and immediately returned to the saline. Bleeding time was measured as the time between the start of the bleeding and cessation of the bleeding. Clot stability time was measured as the time between the cessation of the bleeding and the start of the second bleeding.

### FeCl_3_-induced Carotid Injury model

To induce thrombus formation in the carotid artery, a ferric chloride (FeCl_3_) model of vessel injury was employed as previously described [35]. Mice were anesthetized with ketamine/xylazine (90 mg/kg, 5 mg/kg), a midline cervical incision was made and the left common carotid artery isolated by blunt dissection. The flow probe (Transonic Systems, model 0.5PSB) was placed under the artery and when a stable baseline was reached, a 0.5 × 2 mm strip of filter paper saturated with 10% FeCl_3_ solution was applied to the surface of the carotid artery for 3 minutes. Occlusion time was determined from the addition of the FeCl_3_ solution to the occlusion of the artery (minimum blood flow). There was no difference in baseline blood flow data in carotid arteries among the mouse strains. The flow probe (Model 0.5PSB,Transonic Systems, Ithaca, NY) was in place from baseline measurements to several minutes after the stable occlusion had been reached, or stopped at 30 min if it had not occluded. Blood flow was recorded every 10 sec (Model TS420, Transonic Systems). 

### CaCl_2_ Abdominal Aorta Induced Aneurysm (AAA) Formation

AAA was induced by periaortic application of CaCl_2_ [39]. Mice at age 6~8 weeks were anesthetized (90mg /kg ketamine/ 5mg/kg xylazine) and the abdominal aorta between the renal arteries and bifurcation of the iliac arteries was isolated from surrounding tissue. An image of the aorta was acquired by Spot Image 4.0.3 through a cooled CCD camera attached to an Olympus SZ-PT dissection microscope. After image acquisition, cotton gauze (1 x 0.5 cm) that had been soaked in 0.5 M CaCl_2_ solution was applied to the external surface of the aorta. After 10 minutes the aorta was rinsed with 0.9% sterile saline and the incision was closed. Three weeks later, after the mice were anesthetized and the aorta exposed after dissecting away the surrounding fatty tissue and scarring adhesions, images were collected as above. The aorta diameter was measured from the before and after CaCl_2_ images by ImagePro software. 

### Statistics

Data are presented as the mean ± SEM. One-way ANOVA and Newman-Kuels post-test were used to determine statistical differences between groups. A P value of <0.05 was considered significant. 

## Results

### Chromosome 11 Congenic and Subcongenic Strains

**Figure 1:**
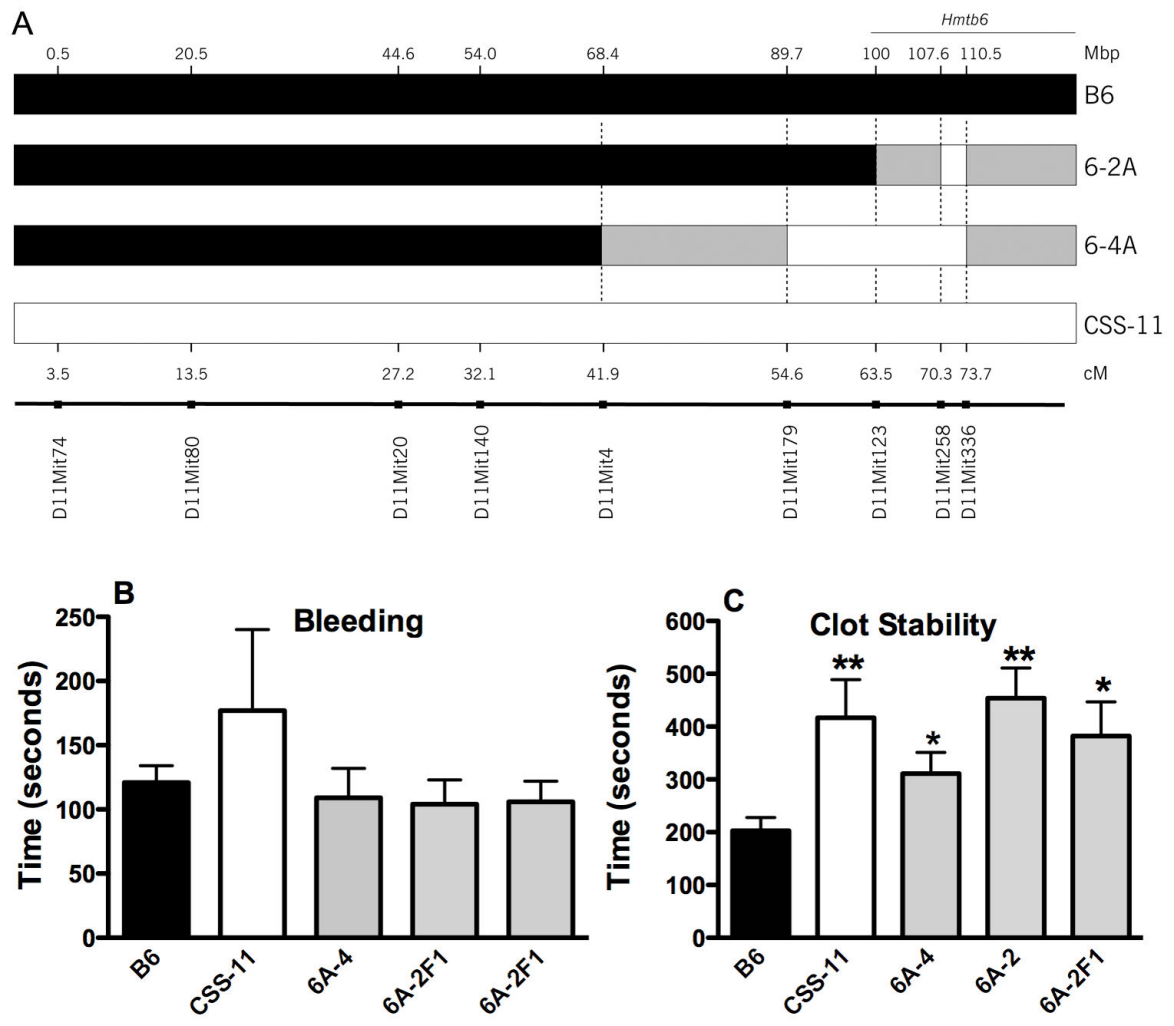
Genotype of Chromosome 11 Congenic and Subcongenic Mice. A. Marker positions. White bars-A/J, grey-uncertain, black-B6. B. First bleeding. C. Time between first and second bleeding. Values are the mean ± SEM, n=10-28, one-way ANOVA, * P< 0.05, **P<0.01.

Our previous study, using F2 progeny from a cross of CSS-11 and B6, identified a QTL for clot stability time in the bleeding/rebleeding assay on mouse distal chromosome 11 that peaked at the genetic marker *D11Mit336* [36]. To verify this QTL (*Hmtb6*), congenic strains were constructed by crossing the CSS-11 mice and B6 mice to produce the congenic strain 6A-4 and the subcongenic strain 6A-2. The position of the A/J regions is indicated in Figure 1A, and boundary markers are summarized in Table 1. The minimum congenic interval is the interval between markers with A/J genotypes on the B6 background. The maximum interval is the interval between two markers with B6 genotypes just outside the minimum interval. The interval between minimum and maximum intervals are of unknown genotype [29]. The minimum A/J interval for 6A-4 is 20.8 Mbp, and for 6A-2, 2.9 Mbp.

**Table 1:** Summary of Congenic and Subcongenic Strains.

		Marker	Region	Marker	Region
Strain	Markers	Positions(cM)	(cM)	Positions(Mbp)	(Mbp)
Minimum					
Chromosome 11					
6A-4	*D11Mit179-D11Mit336*	54.6-73.7	19.1	89.7-110.5	20.8
6A-2	*Dmit258-D11mit336*	70.3-73.7	3.4	107.6-110.5	2.9
Chromosome 5					
6A-1	*rs1680965-D5Mit338*	5-53.2	48.2	21.4-108.6	87.2
4A-2	*rs1680965-D5Mit197*	5-32.9	27.9	21.4-64.6	43.2
3A-1	*rs16809655-D5Mit394*	5-29.8	24.8	21.4-54.3	32.9
4A-1	*D5Mit394-D5Mit338*	29.8-53.2	23.4	54.3-108.6	54.3
3A-2	*rs6297441-D5Mit320*	48.6-65.2	16.6	100.2-126.8	26.6
2A-1	*D5Mit338-D5Mit320*	53.2-65.2	12	108.6-126.8	18.2
Maximum					
Chromosome 11					
6A-4	*D11Mit4-*	41.9-88	80.1	68.4-122	53.6
6A-2	*D11Mit123-*	63.5-88	58.5	100-122	22
*Chromosome 5*					
6A-1	*—D5Mit320*	0-65.2	65.2	0-126.8	126.8
4A-2	*—rs6297441*	0-48.6	48.6	0-100.2	100.2
3A-1	*—D5Mit197*	0-32.9	32.9	0-64.6	64.6
4A-1	*D5Mit13-D5Mit320*	20-65.2	45.2	37.4-126.8	89.4
3A-2	*DMit197-rs13478553*	32.9-77	44.1	64.6-138.8	74.2
2A-1	*rs6297441-rs13478553*	48.6-77	28.4	100.2-138.8	38.6

The minimum congenic interval is the interval between markers with A/J genotypes on the B6 background. The maximum interval is the interval between two markers with B6 genotypes just outside the minimum interval. The interval between minimum and maximum intervals are of unknown genotype [29]. Marker and genomic coordinates were determined from the Mouse Genome Database (MGD), 2012 [37].

### Bleeding Time in Chromosome 11 Congenic and Subcongenic Mice

The homozygous congenic and subcongenic mice were phenotyped using the tail bleeding/rebleeding assay as described in the Methods. The higher bleeding time value for CSS-11 mice was not statistically significant (P > 0.05) compared to B6 mice, and the bleeding times in 6A-4 mice and 6A-2 mice were similar to the bleeding time in the B6 mice (Figure 1B).

### Clot Stability Time in Chromosome 11 Congenic and Subcongenic Mice

Similar to the CSS-11 parental strain (417±71 sec, n=10), the congenic mice (6A-4) exhibited significantly longer (P<0.05) clot stability times (311±40 sec, n=27) compared to the B6 strain (203±25 sec, n=27) (Figure 1C). The clot stability time was 53% longer in the congenic strain than for B6. To further narrow the QTL interval, a subcongenic strain, 6A-2, was developed (Figure 1A, Table 1). The clot stability time (454 ± 57 sec, n=12) in this subcongenic strain mice was 2.2-fold longer (P=0.01) compared to B6 mice (Figure 1C), and compared to B6 mice, the 2A strain accounts for 100% of the increase in clot stability time for CSS-11, whereas the 4A strain accounts for only 50% of the increase suggesting the trait is isolated to the *D11Mit258* to *D11Mtt336* region. Although the region from *D11Mit258* and *D11Mit336* was genotyped as A/J, we cannot completely rule out the uncertain regions as harboring the gene(s) responsible for the phenotype of long clot stability. Hmtb6-2A F1, clot stability time (382 ± 65, n=11), was 2-fold longer (P=0.05) than for B6 mice, suggesting dominant inheritance of the QTL. These results demonstrate that this distal region on chromosome 11 contains a genetic factor (or factors) that dominantly controls the clot stability time. This *Hmtb*6 QTL was narrowly mapped to a minimum region of 2.9 Mbp. In this region 107.6-110.5 Mbp region on Chromosome 11 there are 25 protein-coding genes (Table S3, blue area) and when the two adjoining regions of uncertain genotype were included, there are 429 genes (Table S3). Annotation of these genes Identified 14 genes additional genes related to hemostasis, thrombosis, clotting, platelets, coagulation, bleeding, blood, or fibrinolysis (Table 2). The genes in the A/J fragment are *Abca5*, *Apoh*, *Gna13*, and the genes in the regions of uncertain genotype are *Ace*, *Adam11*, *Cntnap1*, *Gjc1*, *Itga2b*, *Itgb3*, *Pecam1*, *Plcd3*, *Cd300lf*, *Hn1*, and *Jmjd6* (Table 2A). 

**Table 2:** Candidate Genes for Thrombosis Modifiers on Chromosome 11 and Chromosome 5.

cM	Genome Coordinates (Mbp)	Symbol, Name
A. Hmtb6, Chromosome 11, 107.6-110.5 Mbp, 70.3-73.7 cM		
68.84	105967945-105989964 (+)	*Ace*, angiotensin I converting enzyme (peptidyl-dipeptidase A) 1
66.48	109362831-109401369 (+)	*Adam11*, a disintegrin and metallopeptidase domain 11
64.33	110269369-110337716 (-)	*Cntnap1*, contactin associated protein-like1
66.48	102799579-101190724 (-)	*Gjc1*, gap junction protein, gamma 1
66.29	102453297-102470122 (-)	*Itga2b*, integrin alpha 2
67.84	104608000-104670476 (+)	*Itgb3*, integrin beta 3
69.92	106654217-106750628 (-)	*Pecam1*, platelet/endothelial cell adhesion molecule 1
66.71	103070304-103101658 (-)	*Plcd3*, phospholipase C, delta 3
73.53	110269369-110337716 (-)	*Abca5*, ATP-binding cassette, sub-family A (ABC1), member 5
71.80	108343354-108414396 (+)	*Apoh*, apolipoprotein H, beta-2-glycoprotein 1
71.88	109362831-109401369 (+)	*Gna13*, guanine nucleotide binding protein, alpha 13
80.59	115116214-115133992 (-)	*Cd300lf*, CD300 antigen like family member F
80.84	115497353-115514387 (-)	*Hn1*, hematological and neurological expressed sequence 1
81.49	116837432-116843449 (-)	*Jmjd6*, jumonji domain containing 6
B. Hmtb5, Chromosome 5, 100.2-108.6 Mbp, 48.6-65.2 cM		
50.45	103692374-103855322 (+)	*Affl*, AF4/FMR2 family, member 1
52.23	107716657-107726036 (-)	*Gfi1*, growth factor independent 1
52.23	107548967-107597888 (-)	*Glmn*, glomulin, FKBP associated protein
48.51	100679484-100719716 (-)	*Hpse*, heparanase
53.13	108461232-108506976 (+)	*Pcgf3*, polycomb group ring finger 3
53.07	108388391-108506976 (+)	*Pde6b*, phosphodiesterase 6B, cGMP, rod receptor, beta polypeptide
50.68	104459450-108432397 (+)	*Pkd2*, polycystic kidney disease 2
48.49	100553725-100572245 (-)	*Plac8*, placenta-specific 8
50.43	103425192-103598359 (+)	*Ptn13*, protein tyrosine phosphatase, non-receptor type 13
50.68	104435118-104441050 (+)	*Spp1*, secreted phosphoprotein 1
51.9	107106570-107289629 (-)	*Tgfbr3*, transforming growth factor, beta receptor III
C. *Hmtb10*, Chromosome 5, 21.4-54.3 Mbp, 5-29.8 cM		
17.9	34573664-34632308 (+)	*Add1*, adducin 1 (alpha)
19.24	36747374-36748679 (-)	*Bloc1s4*, biogenesis of organelles complex-1, subunit 4, cappuccino
17.52	33247724-33275004 (-)	*Ctbp1*, C-terminal binding protein 1
20.4	38319509-38322310 (+)	*Drd5*, dopamine receptor D5
16.9	30913402-30921278 (-)	*EMILIN1*, elastin microfibril interfacer1
23.81	43744616-43821639 (-)	*Fbxl5*, F-box and leucine-rich repeat protein 5
17.27	31253280-31291114 (-)	*Ift172*, intraflagellar transport 172
10.33	23434441-23504235 (+)	*Mll5*, myeloid/lymphoid or mixed-lineage leukemia 5
20.21	37820485-37824583 (-)	*Msx1*, homeobox, msh-like 1
11.32	24364810-24384474 (+)	*Nos3*, nitric oxide synthase 3, endothelial cell
29.37	53590215-53657445 (+)	*Rbpj*, rcombination signal binding protein for immunoglobulin kappa 3 region
11.93	24802823-24842624 (-)	*Rheb*, Ras homolog enriched in brain
14.39	28456815-28467101 (-)	*Shh*, sonic hedgehog
17.06	31116712-31137630 (+)	*Trim54*, tripartite motif-containing 54
20.63	38526813-38561595 (-)	*Wdr1*, WD repeat domain 1
D. *Hmtb4*, Chromosome 5,108.6-126.8 Mbp, 53.2-65.2 cM		
61.84	121950106-121952989 (-)	*Adam1a*, a disintegrin and metallopeptidase domain 1a
61.83	121950107-121953444 (-)	*Adam1b*, a disintegrin and metallopeptidase domain 1b
60.64	120680202-120711927 (-)	*Dtx1*, deltex 1 homolog (Drosophila)
57.92	118064981-118155458 (-)	*Fbxw8*, F-box and WD-40 dommain protien 8
54.69	112343083-112378414 (+)	*Hps4*, Hermansky-Pudlak syndrome 4 homolog (human)
62.63	122870668-123015080 (-)	*Kdm2b*, lysine (K)-specific demethylase 2B
56.09	115429599-115455698 (+)	*Msi1*, musashi RNA-binding protein 1
62.16	122100951-122113472 (+)	*Myl2*, myosin, light polypeptide 2, regulatory, cardiac, slow
54.8	112688876-112896362 (-)	*Myo18b*, myosin XVIIIb
57.29	117781032-117958840 (+)	*Nos1*, nitric oxide synthase 1, neuronal
62.72	123015074-123030450 (+)	*Orai1*, ORAI calcium release-activated calcium modulator 1
53.49	110339812-110343035 (-)	*P2rx2*, purinergic receptor P2X, ligand-gated ion channel, 2
62.53	122707584-122729738 (+)	P2rx4, purinergic receptor P2X, ligand-gated ion channel, 4
61.72	121130533-121191397 (-)	*Ptpn11*, protein tyrosine phosphatase, non-receptor type 11
55.59	113817798-113830501 (-)	*Selplg*, selectin, platelet (p-selectin) ligand
61.99	121815488-121836859 (-)	*Sh2b3*, SH2B adaptor protein 3
60.34	119670862-119684724 (+)	*Tbx3*, T-box 3
63.03	123528764-123573015 (-)	*Vps33a*, vacuolar protein sorting 33A (yeast)
E. Hmtb 11, Chromosome 5, 0-21.4 Mbp.		
3.43	8660077-8748575 (+)	*Abcb1a*, ATP-binding cassette, sub-family B (MDR/TAP), member 1A
3.43	8798147-8866314 (+)	*Abcb1b*, ATP-binding cassette, sub-family B (MDR/TAP), member 1B
3.43	8893721-8959225 (+)	*Abcb4*, ATP-binding cassette, sub-family B (MDR/TAP), member 4
2.26	3928054-4080209 (+)	*Akap9*, A kinase (PRKA) anchor protein (yotiao) 9
8.11	17781690-17888959 (-)	*Cd36*, CD36 antigen
2.04	3344312-3522225 (+)	*Cdk6*, cyclin-dependent kinase 6
2.3	4081145-4104746 (-)	*Cyp51*, cytochrome P450, family 51
3.69	9118801-9161749 (-)	*Dmtf1*, cyclin D binding myb-like transcription factor 1
9.83	21372642-21378374 (+)	*Fgl2*, fibrinogen-like protein 2
2.61	4753873-4758035 (-)	*Fzd1*, frizzled homolog 1 (Drosophila)
7.07	16553550-16619439 (+)	*Hgf*, hepatocyte growth factor
2.26	3803165-3844515 (+)	*Krit1*, KRIT1, ankyrin repeat containing
9.83	20986645-21055911 (-)	*Ptpn12*, protein tyrosine phosphatase, non-receptor type 12
4.31	13396784-13602565 (+)	*Sema3a*, sema domain, immunoglobulin domain (Ig), short basic domain,
		secreted, (semaphorin) 3A
7.99	17574281-17730268 (+)	*Sema3c*, sema domain, immunoglobulin domain (Ig), short basic domain,
		secreted, (semaphorin) 3C
5.66	14025276-14256689 (+)	*Sema3e*, sema domain, immunoglobulin domain (Ig), short basic domain,
		secreted, (semaphorin) 3E
3.37	7960472-7982213 (+)	*Steap4*, STEAP family member 4

Protein coding genes of QTL intervals annotated were subjected to functional annotation programs, David Bioinformatics Resources 6.7 (http://david.abcc.ncifcrf.gov/home.jsp) [38], from the Mouse Genome Database (MGD), 2012 [37]. Annotation search words–hemostasis, thrombosis, clotting, platelets, coagulation, bleeding, blood, fibrinolysis. Shaded areas are genes of uncertain genotype.

### Chromosome 5 Congenic and Subcongenic Strains

**Figure 2:**
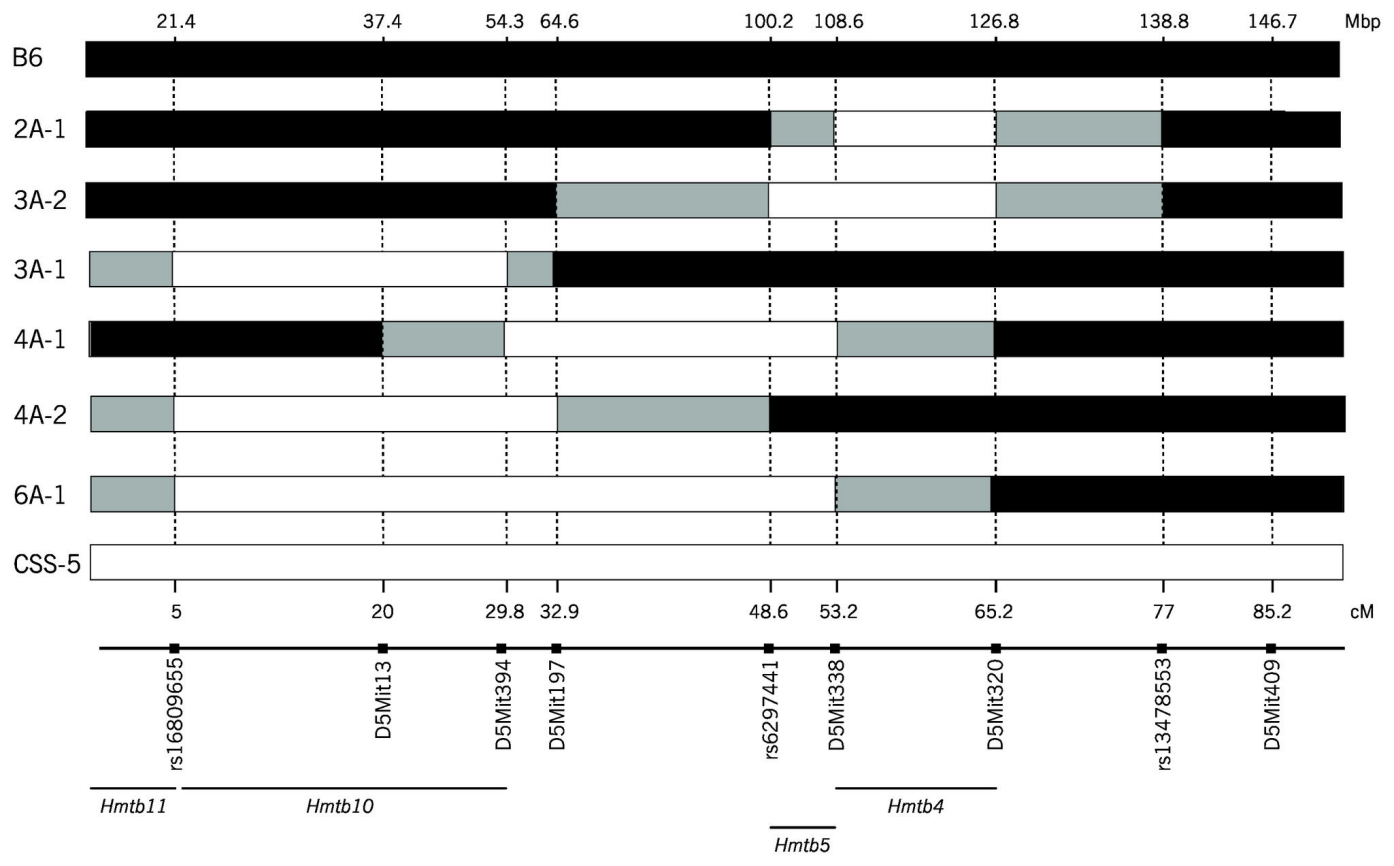
Genotype of Chromosome 5 Congenics and Subcongenic Mice. Marker positions. White bars-A/J, grey-uncertain, black-B6, hatched-heterozygous.

Congenic and subcongenic strains were constructed to verify the overlapping QTL on chromosome 5, *Hmtb*4 and *Hmtb*5, for clot stability and bleeding. Congenic strain 3A-2 and subcongenic strain 2A-1 were constructed for the distal region of chromosome 5 containing the *Hmtb4* and *Hmtb5* QTL peak markers (Table 1, Figure 2). In the QTL analysis there was a suggestive peak for bleeding in the proximal region of chromosome 5, and subcongenic strains 4A-1, 4A-2, and 3A-1 were constructed from the 6A-1 congenic strain for this region of chromosome 5 (Table 1, Figure 2).

### Bleeding Time in Chromosome 5 Congenic and Subcongenic Strains

**Figure 3:**
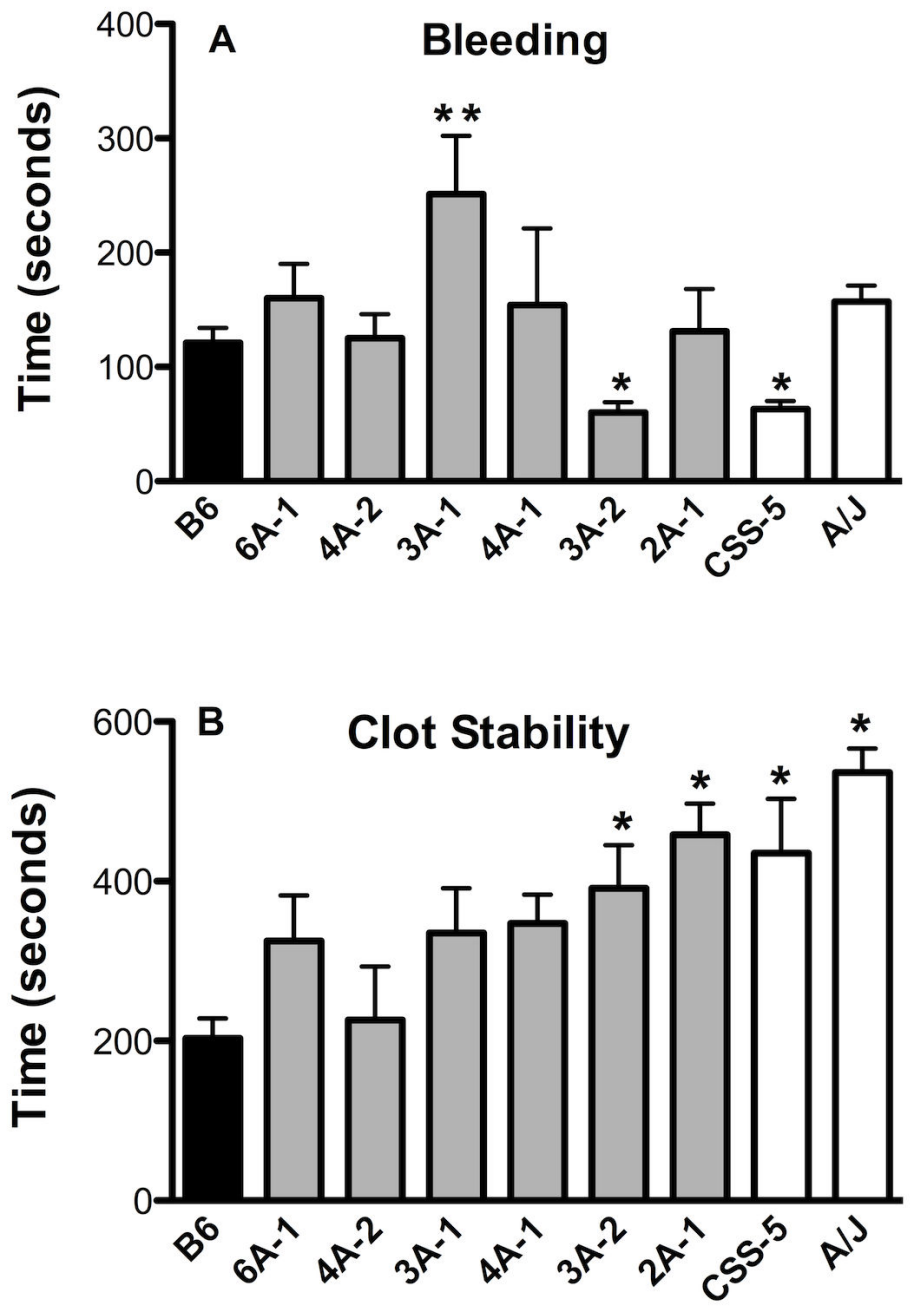
Tail Bleeding/Rebleeding Assay. A. First bleeding time. B. Clot Stability-time between first and second bleeding. Values are the mean ± SEM, n=7-28. One-way ANOVA, *P < 0.05, ** P < 0.01.

Bleeding time in the CSS-5 mice (63±7, n=7) was significantly shorter than for B6 mice (121±13, n=28) as previously reported [35] and the congenic strain, 3A-2 (60±9, n=17) also had the same phenotype. The A/J fragment of 3A-2 strain was slightly longer than for the 2A-1 strain with an additional 35.6 Mbp region between *D5Mit197* and rs6297441 (Table 1). The bleeding time of the 2A-1 was the same as the time in B6 mice suggesting the additional region of 3A-2 harbored the short bleeding time. This narrow region of 3A-2 may be the site for the CSS-5 short bleeding time phenotype (Figure 3A), and the *Hmtb*5 locus identified in the QTL mapping [36]. Thus, for the *Hmtb*5 locus, the minimum A/J interval on 3A-2 strain, 100.2-108.6 Mbp that did not overlap with 2A-1, there are 78 protein-coding genes (Table S4), and functional annotation analysis revealed 11 relevant genes (Table 2).

In the proximal region of chromosome 5, bleeding times for the subcongenic strains 4A-2 and 4A-1 mice were similar to B6 mice (Figure 3A). The 3A-1 mice, carrying the A/J interval from the proximal end to the marker *D5Mit394*, had a longer bleeding time (251±51, n=15, P< 0.01) that was 2-fold longer than A/J or B6 mice (Figure 3A). This suggests an additional locus for bleeding time on chromosome 5 and is consistent with the QTL analysis that identified a suggestive locus at the proximal end of the chromosome. This locus was named *Hmtb10* with the minimum interval of 32.9 Mbp. There are 266 protein-coding genes (Table S5), and the annotation analysis identified 15 genes (Table 2). Thus, there are two loci on chromosome 5 for bleeding, one in the distal region (Hmtb5) and one in the proximal region (*Hmtb10*). 

### Clot Stability Time in Chromosome 5 Congenic and Subcongenic Strains

The subcongenic strain with the smallest A/J interval, 2A-1, had significantly prolonged clot stability time (458±39, n=16), comparable to the CSS-5 parental strain (Figure 3B) and confirms the *Hmtb4* locus on chromosome 5 with the minimum interval of 18.2 Mbp (Figure 2, Table 1). Although 6A-1 (325±57, n=11), 4A-1 (347±47, n=7) and 3A-2 (335±58, n=13) mice had long clot stability times, none of the values were significantly different from the B6 mice (Figure 3B). The subcongenic strain 4A-2 had a clot stability time (226±67, n=7) similar to B6 mice. These data suggest that the distal region of chromosome 5 is the site of the clot stability time (*Hmtb4*). In the region of 108.6-126.8 Mbp on chromosome 5, interval of A/J minimum genotype on 3A-2 strain, there are 272 protein-coding genes (Table S6). Functional annotation analysis identified 18 relevant genes (Table 2). 

### Carotid and Abdominal Aorta Vascular Injury

**Figure 4:**
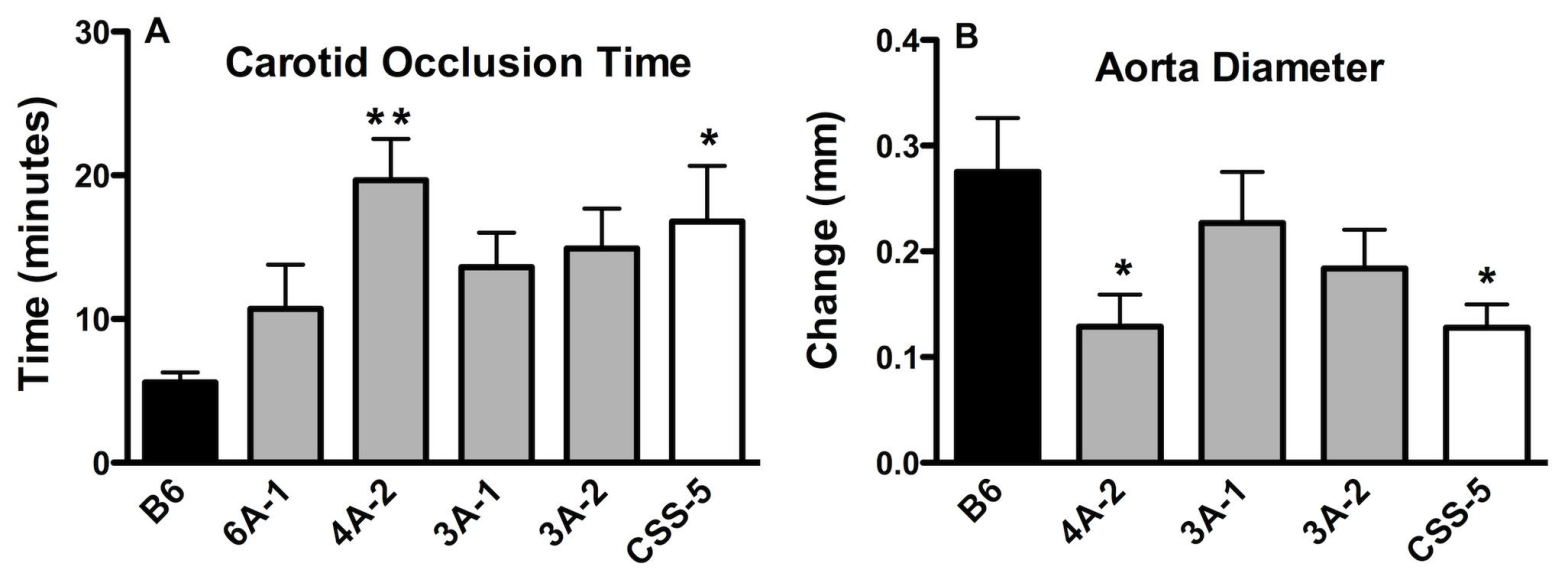
Vascular Injury. A. FeCl_3_ Induced Carotid Injury Occlusion Time. Time after injury for blood flow to cease. n=5-17. B. CaCl_2_ Induced Abdominal Aortic Aneurysm. Change in diameter 3wk after CaCl_2_ treatment. n=7-18, values are the mean ± SEM, one-way ANOVA, * P< 0.05, **P<0.01.

Hemostasis and thrombosis are complex polygenic processes. While QTL link genomic regions to phenotypic data, multiple traits within the QTL region may be responsible for a particular phenotype [40-42]. To further characterize the phenotype of the *Hmtb*4, *Hmtb5*, and *Hmtb10* loci, carotid occlusion time after FeCl_3_ injury (Figure 4A) and abdominal aortic aneurysm formation after CaCl_2_ injury (Figure 4B) were assessed in the congenic strains. In the carotid vascular injury model, a thrombus forms and occludes blood flow. The time required for the occlusion time can detect imbalances in coagulation and platelet functions. The strains 6A-1, 3A-1 and 3A-2, had occlusion times that were not statistically different from B6 or CSS-5 mice (Figure 4A), but the values for 3A-1 and 3A-2 mice were closer to the CSS-5 mice. The 4A-2 strain had a prolonged occlusion time that was 4-fold longer than B6 mice (P < 0.01) and similar to the CSS-5 strain. In addition, the 4A-2 strain had the lowest response to the CaCl_2_ injury and the increase in diameter of the congenic was similar to the CSS-5 mice. The results of these two models, carotid injury and abdominal aorta injury, revealed that another locus, related to thrombosis modification, occurs within this region on chromosome 5, Hmtb11. Compared to 3A-1 mapped at *rs1680965* and *D5Mit394*, the 4A-2 congenic was genotyped at *rs1680965* and *D5Mit197*, only a difference of 10 Mbp. Within this additional region there were only 10 additional protein coding genes and they did not associate with the annotation criteria (Table S7). One possibility is that the regions from 0-21.4 Mbp (rs1680965) in the two congenic strains, 3A-1and 4A-2, were genetically different. In this region (0-21.4 Mbp) there are 89 protein-coding genes (Table S8). Annotation analysis identified 17 genes in this region (Table 2).

### CCS-17 Backcross with Chromosome 5 Congenics

**Figure 5:**
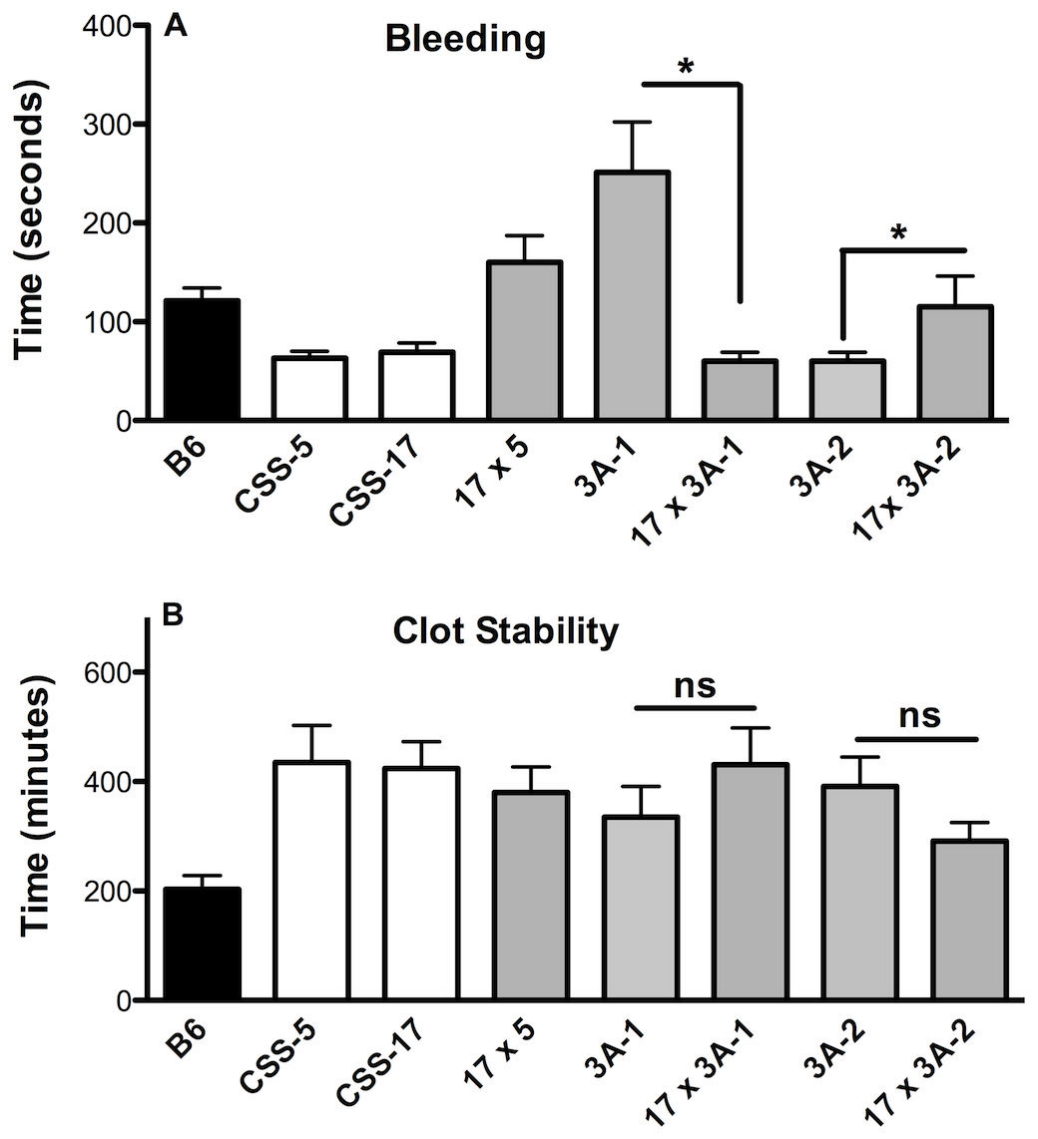
Comparison of Consomic and Congenic crosses. A. First bleeding time. B. Clot Stability-time between first and second bleeding. Values are the mean ± SEM, n=9-24, one-way ANOVA, * P< 0.05.

Previously, we reported an interaction of the genes on chromosome 5 and chromosome 17 that modified bleeding and clot stability in the tail bleeding/rebleeding assay [35]. To test if we could determine if this interaction was in the proximal or distal region on chromosome 5, the 3A-1 (proximal) mice and 3A-2 (distal) mice were crossed with CSS-17 mice (Figure 5A). When the 3A-1 mice with the long bleeding times were crossed with CSS-17 mice, the 17 x 3A-1 mice now had short bleeding times (60±9, n=9) suggesting an inhibition by chromosome 17 on the long bleeding times of 3A-1 mice. When the 3A-2 mice with the short bleeding times were crossed with CSS-17 mice, the 17 x 3A-2 mice had longer bleeding times (115±31, n=24) compared to the 3A-2 mice suggesting an interaction of chromosome 17 with this region. Thus, the genes of A/J chromosome 17 interacted with *Hmtb5* and *Hmtb10* of chromosome 5 to modify bleeding time. 

For clot stability time (Figure 5B), there was no difference between, the 3A-2 and 17 x 3A-2 or between 3A-1 and 17 x 3A-1. The clot stability time in the F1 progeny of the CSS-5 and CSS-17 mice was similar to the value for B6 mice [35], and in the cross of CSS-17 and CSS-5 (17 x 5) the long clot stability time was recovered. Taken together this suggests that long clot stability time requires two alleles for expression, two in the CSS-5 or two from CCS-17 or one allele from each. The one allele from chromosome 17 and either the locus *Hmtb5* (3A-2) or *Hmtb10* (3A-1) in the cross were sufficient for the expression of the phenotype.

## Discussion

**Figure 6:**
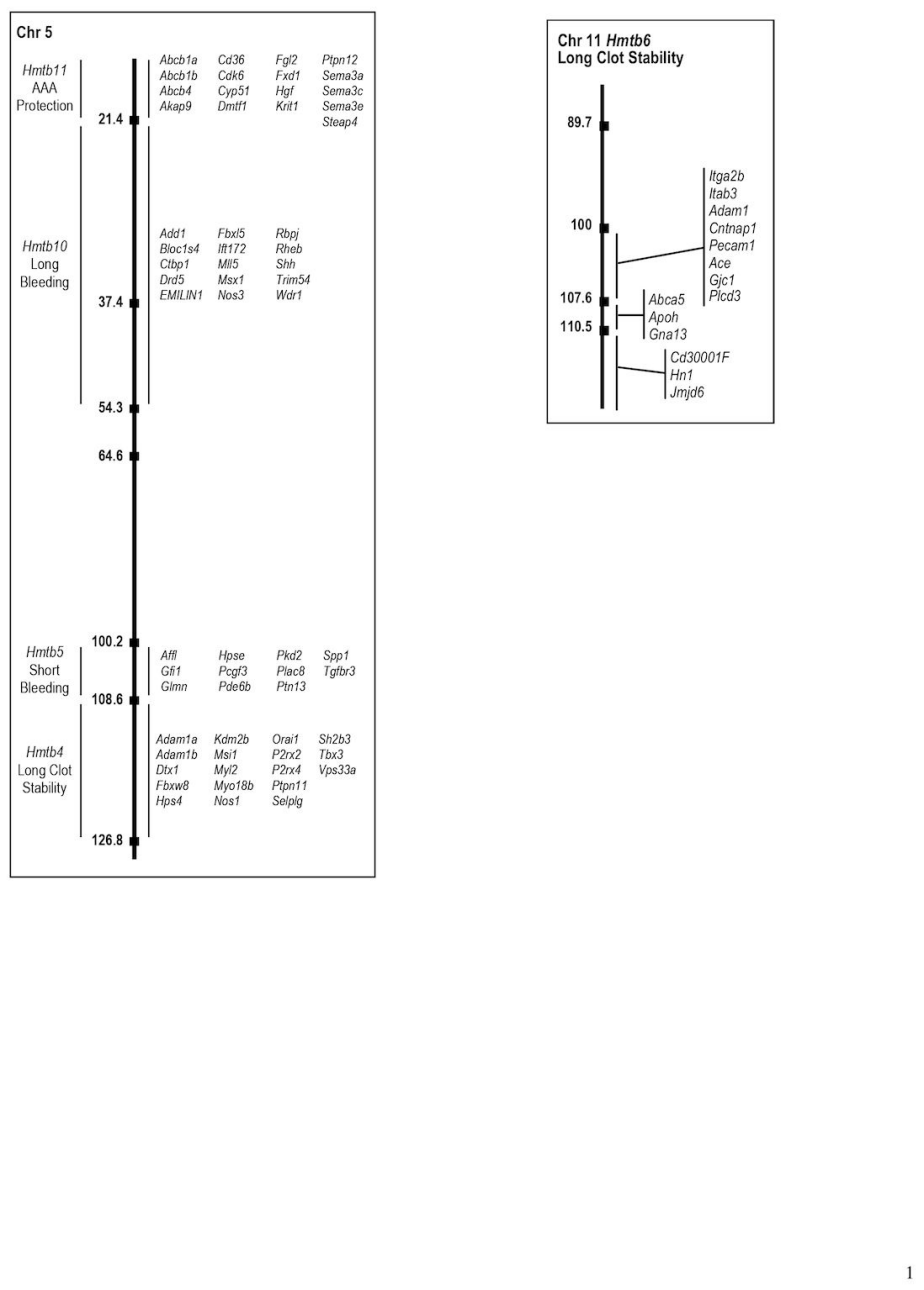
Summary QTL and Candidate Genes for Thrombosis and Hemostasis on Mouse Chromosome 5 and Chromosome 11.

In this study, we confirmed the previously identified QTL, *Hmtb6*, *Hmtb4*, and *Hmtb5* for hemostasis and thrombosis on mouse chromosomes 11 and 5 in congenic and subcongenic strains. Two additional QTL have also been identified, *Hmtb*10 and *Hmtb*11. To our knowledge, this is the first study to confirm QTLs associated with hemostasis and thrombosis using congenic and subcongenic mouse strains. Figure 6 summarizes the QTL and potential candidate genes. 

The phenotype of the congenic and subcongenic strains demonstrates that the gene (or genes) underlying *Hmtb*6 QTL is located in the distal region on mouse chromosome 11, with a minimum interval of 2.9 Mbp. This region contains 25 known and predicted genes and corresponds closely to a syntenic region on human chromosome 17, from q12 to q25.3. No QTL has been identified in this region in human populations for hemostasis and thrombosis. The only gene known to be related to hemostasis and thrombosis on mouse chromosome 11 is *Serpinf2* (α_2_-antiplasmin), an inhibitor of plasmin, the primary enzyme for clot lysis. *Serpinf2* is located at 75.2 Mbp and was excluded from the candidates for this trait from our previous study [36]. This conclusion was further confirmed in our current study. Interestingly, Mohlke et al. [43] identified a QTL, *Mvwf* as a modifier of von Willebrand factor (VWF), that regulates plasma VWF level on mouse chromosome 11, in a region between markers *Ngfr* and *Hoxb9* (95.5-96.2 Mbp). They further found that alterations in *Galgt2*, a candidate at the QTL locus at 95.8 Mbp on chromosome 11, influenced plasma VWF levels [44]. This locus was noted in the *Hmtb6* QTL in the previous study [36], and our current study clearly demonstrates that *Mvwf* is located proximal to the *Hmtb6* locus and outside the maximal interval (100-121.8 Mbp), indicating that a gene (or genes) other than *Mvwf* on chromosome 11 regulates hemostasis and thrombosis. Annotation analysis revealed 3 genes in the *Hmtb6* QTL, *Abca5*, *Apoh*, and *Gna13*. *Abca5* deficient mice develop a dilated cardiomyopathy with large thrombi [45,46]. *Gna13* plays a role in platelet -granule release [47-49]. *Apoh*, more commonly known as beta-2-glycoprotein 1, binds to cardiolipin, inhibits platelets activation, and is involved in anticoagulation [50-52]. These genes have not been commonly associated with thrombosis or hemostasis in humans, and may be novel modifiers. In addition to *Abca5* there are 4 other ATP-binding cassette proteins, and besides *Gna13*, *Rgs9* is also associated with G-protein signaling. Three genes are involved in calcium transport, *Cacng1*, *Cacng4* and Cacng5, and three genes code for protein kinases, *Map2k6*, *Prkar1a*, and *Prkca*. *Wipi1* and *Arsg* are associated with the Golgi and endoplasmic reticulum. In the regions of uncertain genotypes of Hmtb6, there were three genes, *Itga2b*, *Itgb3*, and *Pecam-1* that are associated with bleeding and thrombosis. Mutations of *Itga2b* and *Itgb3* are assoicated with Glanzmann thrombasthenia, the most common inherited disease of platelet function in humans, causing a bleeding disorder [53]. *Pecam-1* deficient mice have reduced occlusion time in the formation of a thrombus [54]. 

Previously [36] we detected a significant locus, Hmtb4, on mouse chromosome 5 for clot stability with a 1-LOD confidence interval 55-66 cM (112.9-127.6 Mbp). From the congenic mapping we confirmed the phenotype of this locus with increased clot stability similar to the consomic strain CSS-5, compared to the parental strain, B6 mice. Functional annotation analysis identified 18 relevant genes. Genes associated with human syndromes or diseases include: polymorphisms in *Hps4* and *Vps33a* genes, which are, associated with Hermansky-Pudlak syndrome type 4 and the absence of platelet dense granules [9,55]. Orai, the major calcium channel in the platelet plasma membrane, has recently been reported to play a role in platelet activation and thrombus formation [56]. *Ptpn11* is a member of the protein tyrosine phosphatase family, and mutations are identified with Noonan syndrome, one of the most common genetic disorders of congenital heart defects [37,57]. Mice homozygous null for *P2rx4*, a purinergic receptor, ligand gated ion channel 4, have hypertension, abnormal artery morphology and vascular remodeling [58]. The *Sh2b3*, adaptor protein 3 or LNK deficient mice have normal tail bleeding time, but in 60% of the deficient mice rebleeding occurred within 60 seconds, suggesting the clot was unstable. In the 3A-2 congenic strain tail bleeding was reduced and rebleeding inhibited, but similar to the Lnk-/- mice, FeCl_2_-induced occlusion time was higher in the CSS-5 mice than in B6 mice and similar to the 3A-2 mice. *Selplg* (P-selectin) is a cell adhesion molecule stored in -granules in platelets and Weibel-Palade bodies in endothelial cells and when these cells are activated P-selectin is released to the surface [59,60]. P-selectin is important in the recruitment of leukocytes to sites of injury [61]. *Adam1a* and *Adam1b* have disintegrin metallopeptidase domains that have been implicated as inhibitors of blood coagulation, but these two proteins have not previously been associated with coagulation [62]. From the previous study mapping the QTLs, the region of the 126.8 -138.6 Mbp was outside the 95% confidence region for the *Hmtb4* [36]. The annotated gene in this region was PAI-1, and we did not find any difference in mRNA expression of PAI-1 in the parent strains, and PAI-1 antigen was not different in the congenic strains. It’s not likely this is the candidate gene in this QTL. Although considered outside the region of confidence from the previous QTL mapping, there are two known genes that could be candidate genes, *Pf4* and *Pdgfr*, in the proximal region of uncertain genotype in 3A-2. 

Hmtb5 locus for bleeding with 1-LOD confidence interval, 45-75 cM (113-128 Mbp) at the distal end of chromosome 5, was previously identified [36]. The congenic strain, 3A-2 with the A/J interval of 100.2-108.6 Mbp, had a short bleeding time similar to the CSS-5 mice suggesting this region was the site of the suppressed bleeding time phenotype in CSS-5. In the subcongenic strain, 2A-1, bleeding time was similar to B6 mice. In the region (100.2-108.6Mbp) of 3A-2 that did not overlap with 2A-1 there were 11 annotated genes for Hmtb5. A potential candidate gene in this region is Hpse, *heparanase*, alpha-endoglucuronidase, a the major enzyme for heparin sulfate degradation. Diabetes and vascular injury increase the expression of *heparanase* and transgenic mice with overexpression of *heparanase* increase thrombosis after mild vascular injury [63,64]. 

This study has identified a new QTL, *Hmtb5* for bleeding in the proximal region of chromosome 5. From the congenic mapping we confirmed the locus identified at the RFLP marker, r*s1680965*, in the QTL mapping for long bleeding time. Annotation analysis identified 15 genes in this region. Two of these genes are associated with blood pressure regulation, Drd5 [62,65] and Nos3 [66-68]. The cappuccino deficiency, a spontaneous mutation of Bloc1s4, is associated with reduced platelet dense granules and prolonged bleeding [69,70]. Another gene of interest is EMILIN1. We identified EMILIN2, with a high homology to EMILIN1, in the QTL on chromosome 17 [71,72], in the Hmtb8 locus and found a thrombosis phenotype. The results of the tail bleeding/rebleeding assay and FeCl_3_ injury model in EMILIN1 deficient mice were not different than for B6 mice (Table S9) suggesting EMILIN1 is not a causative gene in this QTL. 

An additional new QTL was uncovered, *Hmtb11* in the congenic strain, 4A-2, that had an increased occlusion time in the carotid injury model and was protected from abdominal aneurysm after CaCl_2_ injury, which suggests another locus in the proximal region. Genes of particular note from the annotation search are *CD36* that is associated with platelet glycoprotein IV deficiency [73]. *Fgl2*, fibrinogen-like protein2, contributes to immunologically mediated thrombosis in experimental and human viral hepatitis [74]. 

### Limitations of study and future directions

Using congenic and subcongenic mice from two consomic strains, CSS-11 and CSS-5, three QTLs and two additional QTLs were fine-mapped for thrombosis and hemostasis modifiers. Sixty-four potential candidate genes were identified. The limitation of the study is that the causative gene has yet to be identified. Further fine mapping is needed with additional subcongenic strains, as well as expression QTL analysis and quantitative RT-PCR. Future directions will include identification of underlying polymorphisms located in the coding, noncoding or regulatory regions and translating these findings to variation in human genes to assess the relationship of genetic variation to hemostasis and thrombosis risk.

## Supporting Information

Table S1 **Genetic Markers and their Chromosomal Position.**
(DOCX)

Table S2 **RFLP Markers on Chr 5, Primers and Restriction Endonucleases.**
(DOCX)

Table S3 **Protein–coding Genes, *Hmtb6*, Chromosome 11 100-122Mbp.**
(DOCX)

Table S4 **Protein-coding Genes, *Hmtb5*, Chromosome 5, 100.2-108.6 Mbp.**
(DOCX)

Table S5 **Protein-coding Genes, *Hmtb10*, Chromosome 5, 21.4-54.3 Mbp.**
(DOCX)

Table S6 **Protein-coding Genes, *Hmtb4*, Chromosome 5, 108-126.8 Mbp.**
(DOCX)

Table S7 **Protein-coding Genes Non-overlapping Region 4A-2, *Hmtb11*, Chromosome 5, 54.3-64.6 Mbp.**
(DOCX)

Table S8 **Protein-coding Genes, *Hmtb11*, Protein-coding Genes, Chromosome 5, 0-21.4.**
(DOCX)

Table S9 **Candidate gene, Emilin1.**
(DOCX)
