# Supplementary material for: Genetic Dissection of Quantitative Trait Loci for Hemostasis and Thrombosis on Mouse Chromosomes 11 and 5 Using Congenic and Subcongenic Strains
Source: PLoS One. 2013 Oct 17;8(10):e77539. doi: 10.1371/journal.pone.0077539 (PMC3798288; doi:10.1371/journal.pone.0077539)
Supplement: Table S1 — Genetic Markers and their Chromosomal Position. (DOCX) [file pone.0077539.s001.docx]

| **Table S1. Genetic Markers and their Chromosomal Position** | | | | | |  |
| --- | --- | --- | --- | --- | --- | --- |
| **Marker**  **(Chr 11)** | **Position**  **(cM)** | **Position**  **(Mbp)** | **Marker**  **(Chr 5)** | **Position**  **(cM)** | **Position**  **(Mbp)** | |
| *D11Mit74* | 3.5 | 0.5 | *rs16809655* | 5.0 | 21.4 | |
| *D11Mit80* | 13.5 | 20.5 | *D5Mit13* | 20.2 | 37.4 | |
| *D11Mit20* | 27.2 | 44.6 | *D5Mit394* | 29.8 | 54.3 | |
| *D11Mit140* | 32.1 | 54.0 | *D5Mit197* | 32.9 | 64.6 | |
| *D11Mit4* | 41.9 | 68.4 | *rs6297441* | 48.6 | 100.2 | |
| *D11Mit179* | 54.6 | 89.7 | *D5Mit338* | 53.2 | 108.6 | |
| *D11Mit123* | 63.5 | 100.0 | *D5Mit320* | 65.2 | 126.8 | |
| *D11Mit258* | 70.3 | 107.6 | *rs13478553* | 77.0 | 138.8 | |
| *D11Mit336* | 73.7 | 110.5 | *D5Mit409* | 85.2 | 146.7 | |

Marker genomic coordinates were determined from the Mouse Genome

Database (MGD), 2012. Eppig JT, *et al.* Nucleic Acids Res 2012; 40:D881-886.
