# Supplementary material for: Genetic Dissection of Quantitative Trait Loci for Hemostasis and Thrombosis on Mouse Chromosomes 11 and 5 Using Congenic and Subcongenic Strains
Source: PLoS One. 2013 Oct 17;8(10):e77539. doi: 10.1371/journal.pone.0077539 (PMC3798288; doi:10.1371/journal.pone.0077539)
Supplement: Table S2 — RFLP Markers on Chr 5, Primers and Restriction Endonucleases. (DOCX) [file pone.0077539.s002.docx]

| **Table S2. RFLP Markers on Chr 5, Primers and Restriction Endonucleases** | | | |
| --- | --- | --- | --- |
| **SNP ID** | **Forward Primer** | **Reverse Primer** | **Enzyme** |
| *rs16809655* | GCAACCCAGATCAAGCATAAGA | ATGATGAGAAGGTCCCCACA | Sall |
| *rs6297441* | TAAGGCTGGGGAATGGTTTG | GGATTGGGTCTGACAACATAGG | Apall |
| *rs13478553* | CATAGCCCAGCCCTCTGC | GGAGACACCACAAGCAGAATTG | Xhol |

Primers for restriction fragment length polymorphism (RFLP) were previously described. Sa Q, Hart E, Hill AE *et al.* Mamm Genome 2008; 19:406-412.
