# Supplementary material for: Genetic Dissection of Quantitative Trait Loci for Hemostasis and Thrombosis on Mouse Chromosomes 11 and 5 Using Congenic and Subcongenic Strains
Source: PLoS One. 2013 Oct 17;8(10):e77539. doi: 10.1371/journal.pone.0077539 (PMC3798288; doi:10.1371/journal.pone.0077539)
Supplement: Table S4 — Protein-coding Genes, Hmtb5, Chromosome 5, 100.2-108.6 Mbp. (DOCX) [file pone.0077539.s004.docx]

| **Table S4. Protein-coding Genes**  ***Hmtb5*, Chromosome 5, 100.2-108.6 Mbp** | | | | |  |  |  |  |
| --- | --- | --- | --- | --- | --- | --- | --- | --- |
| **cM** | **Genome Coordinates (Mbp)** | | | **Symbol** | Name |  |  |  |
| 50.45 | 103648039 | 103656010 | - | 1700016H13Rik | RIKEN cDNA 1700016H13 gene | |  |  |
| 52.23 | 107508944 | 107551549 | + | 1700028K03Rik | RIKEN cDNA 1700028K03 gene | |  |  |
| 52.23 | 107497378 | 107580596 | + | A830010M20Rik | RIKEN cDNA A830010M20 gene | |  |  |
| 50.68 | 104935057 | 104982718 | - | Abcg3 | ATP-binding cassette, sub-family G (WHITE), member 3 | | | |
| 50.45 | 103692374 | 103855322 | + | Aff1 | AF4/FMR2 family, member 1 | |  |  |
| 48.52 | 100845713 | 100899102 | + | Agpat9 | 1-acylglycerol-3-phosphate O-acyltransferase 9 | | |  |
| 49.53 | 102481391 | 102897829 | + | Arhgap24 | Rho GTPase activating protein 24 | |  |  |
| 53.11 | 108433244 | 108434448 | - | Atp5k | ATP synthase, H+ transporting, mitochondrial F1F0 complex, subunit e | | | |
| 51.43 | 106452514 | 106458440 | - | Barhl2 | BarH-like 2 (Drosophila) | |  |  |
| 50.68 | 104508352 | 104522383 | + | BC005561 | cDNA sequence BC005561 | |  |  |
| 52.23 | 107331159 | 107387058 | + | Brdt | bromodomain, testis-specific | |  |  |
| 52.23 | 107437997 | 107491628 | + | Btbd8 | BTB (POZ) domain containing 8 | |  |  |
| 52.55 | 108132914 | 108232947 | + | Ccdc18 | coiled-coil domain containing 18 | |  |  |
| 51.43 | 106964322 | 106984432 | + | Cdc7 | cell division cycle 7 (S. cerevisiae) | | |  |
| 48.93 | 101765130 | 101823858 | + | Cds1 | CDP-diacylglycerol synthase 1 | |  |  |
| 48.49 | 100518309 | 100547803 | + | Cops4 | COP9 (constitutive photomorphogenic) homolog, subunit 4 | | | |
| 48.5 | 100654723 | 100675140 | - | Coq2 | coenzyme Q2 homolog, prenyltransferase (yeast) | | |  |
| 53.17 | 108518554 | 108550024 | - | Cplx1 | complexin 1 |  |  |  |
| 50.61 | 104202617 | 104214102 | + | Dmp1 | dentin matrix protein 1 | |  |  |
| 52.82 | 108268897 | 108280526 | + | Dr1 | down-regulator of transcription 1 | |  |  |
| 50.59 | 104170712 | 104180127 | + | Dspp | dentin sialophosphoprotein | |  |  |
| 52.23 | 107402736 | 107430035 | + | Ephx4 | epoxide hydrolase 4 | |  |  |
| 52.23 | 107744795 | 107875107 | - | Evi5 | ecotropic viral integration site 5 | |  |  |
| 48.52 | 100805192 | 100820965 | - | Fam175a | family with sequence similarity 175, member A | | |  |
| 52.23 | 107908053 | 107987085 | - | Fam69a | family with sequence similarity 69, member A | | |  |
| 53.2 | 108569411 | 108629739 | - | Gak | cyclin G associated kinase | |  |  |
| 50.68 | 105215699 | 105239533 | - | Gbp10 | guanylate-binding protein 10 | |  |  |
| 50.68 | 105323042 | 105346472 | - | Gbp11 | guanylate binding protein 11 | |  |  |
| 50.68 | 105115767 | 105139586 | - | Gbp4 | guanylate binding protein 4 | |  |  |
| 50.68 | 105270704 | 105293699 | - | Gbp6 | guanylate binding protein 6 | |  |  |
| 50.68 | 105014153 | 105053561 | - | Gbp8 | guanylate-binding protein 8 | |  |  |
| 50.68 | 105078394 | 105110292 | - | Gbp9 | guanylate-binding protein 9 | |  |  |
| 52.23 | 107716657 | 107726036 | - | Gfi1 | growth factor independent 1 | |  |  |
| 52.23 | 107548967 | 107597888 | - | Glmn | glomulin, FKBP associated protein | | |  |
| 50.68 | 104455138 | 104456467 | - | Gm10047 | predicted gene 10047 | |  |  |
| 51.43 | 106609098 | 106609201 | + | Gm17304 | predicted gene, 17304 | |  |  |
| 50.54 | 104070064 | 104077608 | - | Gm17660 | predicted gene, 17660 | |  |  |
| 50.68 | 104662448 | 104695330 | + | Gm20385 | predicted gene, 20385 | |  |  |
| 48.46 | 100196611 | 100200724 | + | Gm9932 | predicted gene 9932 | |  |  |
| 48.52 | 100762145 | 100798598 | - | Helq | helicase, POLQ-like | |  |  |
| 51.43 | 106840192 | 106926321 | - | Hfm1 | HFM1, ATP-dependent DNA helicase homolog (S. cerevisiae) | | | |
| 48.51 | 100679484 | 100719716 | - | Hpse | heparanase |  |  |  |
| 50.52 | 103989762 | 104021919 | - | Hsd17b11 | hydroxysteroid (17-beta) dehydrogenase 11 | | |  |
| 50.46 | 103955440 | 103977410 | - | Hsd17b13 | hydroxysteroid (17-beta) dehydrogenase 13 | | |  |
| 50.68 | 104299171 | 104311469 | + | Ibsp | integrin binding sialoprotein | |  |  |
| 50.45 | 103861973 | 103911259 | - | Klhl8 | kelch-like 8 |  |  |  |
| 48.48 | 100441918 | 100500639 | - | Lin54 | lin-54 homolog (C. elegans) | |  |  |
| 52.23 | 107431549 | 107435039 | + | Lpcat2b | lysophosphatidylcholine acyltransferase 2B | | |  |
| 50.68 | 105415775 | 105486189 | + | Lrrc8b | leucine rich repeat containing 8 family, member B | | |  |
| 50.68 | 105519388 | 105613018 | + | Lrrc8c | leucine rich repeat containing 8 family, member C | | |  |
| 50.68 | 105699969 | 105832436 | + | Lrrc8d | leucine rich repeat containing 8D | |  |  |
| 49.61 | 102912354 | 103211334 | - | Mapk10 | mitogen-activated protein kinase 10 | | |  |
| 50.68 | 104325329 | 104338611 | + | Mepe | matrix extracellular phosphoglycoprotein with ASARM motif (bone) | | | |
| 53.11 | 108441054 | 108448891 | - | Mfsd7a | major facilitator superfamily domain containing 7A | | |  |
| 48.52 | 100798627 | 100804471 | + | Mrps18c | mitochondrial ribosomal protein S18C | | |  |
| 52.41 | 108065674 | 108109004 | + | Mtf2 | metal response element binding transcription factor 2 | | | |
| 48.84 | 101658139 | 101665226 | - | Nkx6-1 | NK6 homeobox 1 | |  |  |
| 50.54 | 104046306 | 104065379 | + | Nudt9 | nudix (nucleoside diphosphate linked moiety X)-type motif 9 | | | |
| 53.13 | 108461232 | 108506976 | + | Pcgf3 | polycomb group ring finger 3 | |  |  |
| 53.07 | 108388391 | 108432397 | + | Pde6b | phosphodiesterase 6B, cGMP, rod receptor, beta polypeptide | | | |
| 52.9 | 108312609 | 108349355 | + | Pigg | phosphatidylinositol glycan anchor biosynthesis, class G | | | |
| 50.68 | 104459450 | 104505819 | + | Pkd2 | polycystic kidney disease 2 | |  |  |
| 48.49 | 100553725 | 100572245 | - | Plac8 | placenta-specific 8 | |  |  |
| 50.43 | 103425192 | 103598359 | + | Ptpn13 | protein tyrosine phosphatase, non-receptor type 13 | | | |
| 52.23 | 107597373 | 107661838 | + | Rpap2 | RNA polymerase II associated protein 2 | | |  |
| 52.23 | 107900502 | 107909005 | + | Rpl5 | ribosomal protein L5 | |  |  |
| 48.48 | 100361650 | 100416234 | - | Sec31a | Sec31 homolog A (S. cerevisiae) | |  |  |
| 50.45 | 103605711 | 103629403 | - | Slc10a6 | solute carrier family 10 (sodium/bile acid cotransporter family), member 6 | | | |
| 50.55 | 104079111 | 104114088 | - | Sparcl1 | SPARC-like 1 |  |  |  |
| 50.68 | 104435118 | 104441050 | + | Spp1 | secreted phosphoprotein 1 | |  |  |
| 51.9 | 107106570 | 107289629 | - | Tgfbr3 | transforming growth factor, beta receptor III | | |  |
| 52.49 | 108106366 | 108132620 | - | Tmed5 | transmembrane emp24 protein transport domain containing 5 | | | |
| 52.23 | 107830099 | 107831776 | + | Ube2d2b | ubiquitin-conjugating enzyme E2D 2B | | |  |
| 48.95 | 101832956 | 102069921 | - | Wdfy3 | WD repeat and FYVE domain containing 3 | | |  |
| 50.88 | 105876565 | 105915818 | + | Zfp326 | zinc finger protein 326 | |  |  |
| 50.68 | 104571742 | 104644180 | + | Zfp33b | zinc finger protein 33B | |  |  |
| 51.43 | 106500894 | 106697287 | - | Zfp644 | zinc finger protein 644 | |  |  |
| 50.68 | 104814343 | 104828957 | - | Zfp951 | zinc finger protein 951 | |  |  |

Genomic coordinates of genes were determined from the Mouse Genome Database (MGD), 2012. Eppig JT, *et al.* Nucleic Acids Res 2012; 40:D881-886.
