# Supplementary material for: Genetic Dissection of Quantitative Trait Loci for Hemostasis and Thrombosis on Mouse Chromosomes 11 and 5 Using Congenic and Subcongenic Strains
Source: PLoS One. 2013 Oct 17;8(10):e77539. doi: 10.1371/journal.pone.0077539 (PMC3798288; doi:10.1371/journal.pone.0077539)
Supplement: Table S5 — Protein-coding Genes, Hmtb10, Chromosome 5, 21.4-54.3 Mbp. (DOCX) [file pone.0077539.s005.docx]

| \|  \|  \|  \| \| --- \| --- \| --- \| \| **Table S5. Protein-coding Genes *Hmtb10,* Chromosome 5, 21.4-54.3 Mbp** \| \|  \| \| **cM** \| **Genome Coordinates** \| **Symbol, Name** \| \| 12.06 \| 24985842-24998168 (+) \| 1500035N22Rik, RIKEN cDNA 1500035N22 gene \| \| 16.57 \| 30466047-30484088 (+) \| 1700001C02Rik, RIKEN cDNA 1700001C02 gene \| \| 29.05 \| 53267106-53283510 (+) \| 1810013D10Rik, RIKEN cDNA 1810013D10 gene \| \| 15.92 \| 30103889-30105359 (-) \| 3110082J24Rik, RIKEN cDNA 3110082J24 gene \| \| 17.27 \| 31485859-31488476 (+) \| 4930548H24Rik, RIKEN cDNA 4930548H24 gene \| \| 11.93 \| 24543434-24555469 (-) \| 4931409K22Rik, RIKEN cDNA 4931409K22 gene \| \| 12.45 \| 26098668-26105362 (-) \| 5031410I06Rik, RIKEN cDNA 5031410I06 gene \| \| 11.57 \| 24393663-24410054 (+) \| *Abcb8,* ATP-binding cassette, sub-family B (MDR/TAP), member 8 \| \| 11.93 \| 24565341-24577467 (-) \| *Abcf2,* ATP-binding cassette, sub-family F (GCN20), member 2 \| \| 16.9 \| 30950066-30955091 (+) \| *Abhd1,* abhydrolase domain containing 1 \| \| 18.75 \| 35757880-35884973 (+) \| *Ablim2,* actin-binding LIM protein 2 \| \| 18.32 \| 35583040-35613801 (+) \| *Acox3,* acyl-Coenzyme A oxidase 3, pristanoyl \| \| 12.35 \| 25759997-25850342 (+) \| *Actr3b,* ARP3 actin-related protein 3B \| \| 17.9 \| 34573664-34632308 (+) \| *Add1,* adducin 1 (alpha) \| \| 18.09 \| 35278566-35281761 (+) \| *Adra2c,* adrenergic receptor, alpha 2c \| \| 19.02 \| 35893319-36003923 (+) \| *Afap1,* actin filament associated protein 1 \| \| 11.81 \| 24452177-24502047 (+) \| *Agap3,* ArfGAP with GTPase domain, ankyrin repeat and PH domain 3 \| \| 16.9 \| 30888694-30906965 (+) \| *Agbl5,* ATP/GTP binding protein-like 5 \| \| 28.81 \| 52834015-52867797 (+) \| *Anapc4,* anaphase promoting complex subunit 4 \| \| 9.97 \| 21645813-21662694 (+) \| *Armc10,* armadillo repeat containing 10 \| \| 11.93 \| 24532697-24540978 (-) \| *Asb10,* ankyrin repeat and SOCS box-containing 10 \| \| 11.72 \| 24413451-24417834 (+) \| *Asic3,* acid-sensing (proton-gated) ion channel 3 \| \| 11.49 \| 24384181-24392143 (-) \| *Atg9b,* autophagy related 9B \| \| 16.94 \| 31048312-31054633 (+) \| *Atraid,* all-trans retinoic acid induced differentiation factor \| \| 19.24 \| 36747374-36748679 (-) \| *Bloc1s4,* biogenesis of organelles complex-1, subunit 4, cappuccino \| \| 22.58 \| 41787542-41844168 (-) \| *Bod1l,* biorientation of chromosomes in cell division 1-like \| \| 17.27 \| 31697684-32084962 (+) \| *Bre,* brain and reproductive organ-expressed protein \| \| 23.84 \| 43818886-43843247 (+) \| *Bst1,* bone marrow stromal cell antigen 1 \| \| 23.78 \| 43515588-43618817 (+) \| *C1qtnf7,* C1q and tumor necrosis factor related protein 7 \| \| 16.95 \| 31054780-31078479 (+) \| *Cad,* carbamoyl-phosphate synthetase 2, aspartate transcarbamylase, and dihydroorotase \| \| 23.78 \| 43662379-43740972 (+) \| *Cc2d2a,* coiled-coil and C2 domain containing 2A \| \| 9.83 \| 21292961-21424677 (-) \| *Ccdc146,* coiled-coil domain containing 146 \| \| 27.94 \| 52374651-52471543 (-) \| *Ccdc149,* coiled-coil domain containing 149 \| \| 16.42 \| 30341663-30366708 (+) \| *Ccdc164,* coiled-coil domain containing 164 \| \| 19.14 \| 36484588-36488172 (+) \| *Ccdc96,* coiled-coil domain containing 96 \| \| 29.52 \| 53698485-53707704 (-) \| *Cckar,* cholecystokinin A receptor \| \| 12.35 \| 25516067-25518021 (+) \| *Cct8l1,* chaperonin containing TCP1, subunit 8 (theta)-like 1 \| \| 23.85 \| 43868809-43912375 (+) \| *Cd38,* CD38 antigen \| \| 11.73 \| 24418250-24423530 (-) \| *Cdk5,* cyclin-dependent kinase 5 \| \| 16.76 \| 30666777-30674827 (+) \| *Cenpa,* centromere protein A \| \| 16.9 \| 30933146-30945480 (-) \| *Cgref1,* cell growth regulator with EF hand domain 1 \| \| 11.93 \| 24586741-24592487 (+) \| *Chpf2,* chondroitin polymerizing factor 2 \| \| 16.59 \| 30485583-30545836 (-) \| *Cib4,* calcium and integrin binding family member 4 \| \| 20.81 \| 38706457-38876693 (-) \| *Clnk,* cytokine-dependent hematopoietic cell linker \| \| 24.93 \| 45453751-45464149 (+) \| *Clrn2,* clarin 2 \| \| 13.99 \| 28200819-28245792 (-) \| *Cnpy1,* canopy 1 homolog (zebrafish) \| \| 23.65 \| 43233956-43289724 (+) \| *Cpeb2,* cytoplasmic polyadenylation element binding protein 2 \| \| 18.16 \| 35502215-35525698 (-) \| *Cpz,* carboxypeptidase Z \| \| 19.96 \| 37242080-37292132 (+) \| *Crmp1,* collapsin response mediator protein 1 \| \| 11.93 \| 24751003-24758008 (-) \| *Crygn,* crystallin, gamma N \| \| 17.52 \| 33247724-33275004 (-) \| *Ctbp1,* C-terminal binding protein 1 \| \| 20.21 \| 37735519-37739820 (+) \| *Cytl1,* cytokine-like 1 \| \| 19.18 \| 36584788-36696024 (-) \| *D5Ertd579e,* DNA segment, Chr 5, ERATO Doi 579, expressed \| \| 17.35 \| 32863701-32994231 (+) \| *Depdc5,* DEP domain containing 5 \| \| 27.51 \| 52150209-52190519 (-) \| *Dhx15,* DEAH (Asp-Glu-Ala-His) box polypeptide 15 \| \| 15.27 \| 29735688-29786478 (+) \| *Dnajb6,* DnaJ (Hsp40) homolog, subfamily B, member 6 \| \| 9.97 \| 21757267-21785251 (-) \| *Dnajc2,* DnaJ (Hsp40) homolog, subfamily C, member 2 \| \| 17.03 \| 31108319-31112524 (+) \| *Dnajc5g,* DnaJ (Hsp40) homolog, subfamily C, member 5 gamma \| \| 18 \| 35056813-35087839 (+) \| *Dok7,* docking protein 7 \| \| 12.92 \| 26817203-27727505 (+) \| *Dpp6,* dipeptidylpeptidase 6 \| \| 16.8 \| 30711564-30799375 (+) \| *Dpysl5,* dihydropyrimidinase-like 5 \| \| 20.4 \| 38319509-38322310 (+) \| *Drd5,* dopamine receptor D5 \| \| 12.35 \| 25222848-25223153 (-) \| *E130116L18Rik,* RIKEN cDNA E130116L18 gene \| \| 17.24 \| 31187558-31193430 (-) \| *Eif2b4,* eukaryotic translation initiation factor 2B, subunit 4 delta \| \| 16.9 \| 30913402-30921278 (+) \| *Emilin1,* elastin microfibril interfacer 1 \| \| 13.94 \| 28165696-28173612 (+) \| *En2,* engrailed 2 \| \| 16.22 \| 30232581-30272012 (+) \| *Ept1,* ethanolaminephosphotransferase 1 (CDP-ethanolamine-specific) \| \| 20.06 \| 37289098-37336894 (-) \| *Evc,* Ellis van Creveld gene syndrome \| \| 20.15 \| 37338499-37425055 (+) \| *Evc2,* Ellis van Creveld syndrome 2 \| \| 10.53 \| 23915276-24030690 (-) \| *Fam126a,* family with sequence similarity 126, member A \| \| 25.04 \| 45529706-45639501 (-) \| *Fam184b,* family with sequence similarity 184, member B \| \| 9.83 \| 21424958-21482124 (+) \| *Fam185a,* family with sequence similarity 185, member A \| \| 17.88 \| 34426337-34485747 (+) \| *Fam193a,* family with sequence similarity 193, member A \| \| 17.83 \| 33600351-33629635 (-) \| *Fam53a,* family with sequence similarity 53, member A \| \| 11.78 \| 24438440-24445287 (-) \| *Fastk,* Fas-activated serine/threonine kinase \| \| 9.83 \| 21483847-21645605 (-) \| *Fbxl13,* F-box and leucine-rich repeat protein 13 \| \| 23.81 \| 43744616-43821639 (-) \| *Fbxl5,* F-box and leucine-rich repeat protein 5 \| \| 23.95 \| 43978865-43981762 (-) \| *Fgfbp1,* fibroblast growth factor binding protein 1 \| \| 17.83 \| 33721724-33737067 (+) \| *Fgfr3,* fibroblast growth factor receptor 3 \| \| 17.27 \| 31292255-31295877 (-) \| *Fndc4,* fibronectin type III domain containing 4 \| \| 17.33 \| 32136472-32157831 (+) \| *Fosl2,* fos-like antigen 2 \| \| 12.35 \| 25222882-25265918 (+) \| *Galnt11,* UDP-N-acetyl-alpha-D-galactosamine:polypeptide \| \|  \|  \| N-acetylgalactosaminyltransferase 11 \| \| 12.33 \| 25181460-25220297 (+) \| *Galntl5,* UDP-N-acetyl-alpha-D-galactosamine:polypeptide \| \|  \|  \| N-acetylgalactosaminyltransferase-like 5 \| \| 15.92 \| 30105161-30118378 (+) \| *Gareml,* GRB2 associated, regulator of MAPK1-like \| \| 11.93 \| 24502757-24527276 (-) \| *Gbx1,* gastrulation brain homeobox 1 \| \| 17.27 \| 31297581-31327300 (+) \| *Gckr,* glucokinase regulatory protein \| \| 12.45 \| 26115717-26121421 (-) \| *Gm10220,* predicted gene 10220 \| \| 19.84 \| 37185897-37193023 (+) \| *Gm1043,* predicted gene 1043 \| \| 17.33 \| 32130283-32133168 (-) \| *Gm10463,* predicted gene 10463 \| \| 12.44 \| 26082574-26089291 (-) \| *Gm10471,* predicted gene 10471 \| \| 11.81 \| 24451174-24451707 (-) \| *Gm10472,* predicted gene 10472 \| \| 23 \| 42067960-42216798 (+) \| *Gm16223,* predicted gene 16223 \| \| 17.83 \| 33983437-33985013 (+) \| *Gm1673,* predicted gene 1673 \| \| 10.28 \| 23333807-23339204 (+) \| *Gm17434,* predicted gene, 17434 \| \| 13.8 \| 27971436-27976845 (+) \| *Gm17695,* predicted gene, 17695 \| \| 12.44 \| 25999022-26004798 (-) \| *Gm1979,* predicted gene 1979 \| \| 17.33 \| 32737230-32827109 (-) \| *Gm20671,* predicted gene 20671 \| \| 24.24 \| 44700876-44706890 (-) \| *Gm3364,* predicted gene 3364 \| \| 23.89 \| 43943902-43944483 (-) \| *Gm5292,* predicted gene 5292 \| \| 17.86 \| 34306715-34307176 (-) \| *Gm5553,* predicted gene 5553 \| \| 12.44 \| 26017278-26022916 (-) \| *Gm5862,* predicted gene 5862 \| \| 12.44 \| 26051883-26058581 (-) \| *Gm7347,* predicted gene 7347 \| \| 12.72 \| 26257691-26264308 (+) \| *Gm7361,* predicted gene 7361 \| \| 9.97 \| 21613991-21614530 (-) \| *Gm8539,* predicted gene 8539 \| \| 17 \| 31094992-31097294 (+) \| *Gm9924,* predicted gene 9924 \| \| 17.27 \| 31240675-31241232 (-) \| *Gm9970,* predicted gene 9970 \| \| 17.27 \| 31494741-31512903 (+) \| *Gpn1,* GPN-loop GTPase 1 \| \| 16.13 \| 30193431-30205722 (-) \| *Gpr113,* G protein-coupled receptor 113 \| \| 26.85 \| 49959951-50058996 (-) \| *Gpr125,* G protein-coupled receptor 125 \| \| 17.91 \| 34660379-34755305 (+) \| *Grk4,* G protein-coupled receptor kinase 4 \| \| 19.14 \| 36464998-36474080 (+) \| *Grpel1,* GrpE-like 1, mitochondrial \| \| 17.15 \| 31151142-31180144 (-) \| *Gtf3c2,* general transcription factor IIIC, polypeptide 2, beta \| \| 15.95 \| 30118301-30155162 (-) \| *Hadha,* hydroxyacyl-Coenzyme A dehydrogenase/3-ketoacyl-Coenzyme A \| \|  \|  \| thiolase/enoyl-Coenzyme A hydratase (trifunctional protein), alpha subunit \| \| 16.04 \| 30155248-30184593 (+) \| *Hadhb,* hydroxyacyl-Coenzyme A dehydrogenase/3-ketoacyl-Coenzyme A \| \|  \|  \| thiolase/enoyl-Coenzyme A hydratase (trifunctional protein), beta subunit \| \| 17.84 \| 34153921-34169445 (-) \| *Haus3,* HAUS augmin-like complex, subunit 3 \| \| 17.99 \| 35041553-35048436 (+) \| *Hgfac,* hepatocyte growth factor activator \| \| 18.14 \| 35388858-35399730 (+) \| *Hmx1,* H6 homeobox 1 \| \| 21.14 \| 39613935-39755475 (-) \| *Hs3st1,* heparan sulfate (glucosamine) 3-O-sulfotransferase 1 \| \| 13.24 \| 27841947-27855086 (+) \| *Htr5a,* 5-hydroxytryptamine (serotonin) receptor 5A \| \| 18.49 \| 35652041-35679782 (-) \| *Htra3,* HtrA serine peptidase 3 \| \| 17.92 \| 34761740-34912534 (+) \| *Htt,* huntingtin \| \| 17.27 \| 31253280-31291114 (-) \| *Ift172,* intraflagellar transport 172 \| \| 15.7 \| 30013161-30019968 (+) \| *Il6*, interleukin 6 \| \| 13.94 \| 28071363-28078662 (+) \| *Insig1,* insulin induced gene 1 \| \| 19.66 \| 37028335-37148001 (+) \| *Jakmip1,* janus kinase and microtubule interacting protein 1 \| \| 10.94 \| 24319589-24351604 (-) \| *Kcnh2,* potassium voltage-gated channel, subfamily H (eag-related), member 2 \| \| 26.26 \| 48389502-49524904 (-) \| *Kcnip4,* Kv channel interacting protein 4 \| \| 16.68 \| 30588170-30625270 (+) \| *Kcnk3,* potassium channel, subfamily K, member 3 \| \| 16.9 \| 30921556-30931246 (+) \| *Khk,* ketohexokinase \| \| 10.67 \| 24100590-24161223 (+) \| *Klhl7,* kelch-like 7 (Drosophila) \| \| 17.27 \| 31251691-31253202 (+) \| *Krtcap3,* keratinocyte associated protein 3 \| \| 24.98 \| 45493374-45512674 (+) \| *Lap3,* leucine aminopeptidase 3 \| \| 25.27 \| 45697181-45857615 (-) \| *Lcorl,* ligand dependent nuclear receptor corepressor-like \| \| 24.24 \| 44472133-44799707 (-) \| *Ldb2,* LIM domain binding 2 \| \| 17.83 \| 33739673-33782817 (-) \| *Letm1,* leucine zipper-EF-hand containing transmembrane protein 1 \| \| 28.25 \| 52537864-52566280 (-) \| *Lgi2,* leucine-rich repeat LGI family, member 2 \| \| 10.07 \| 22746193-23275597 (+) \| *Lhfpl3,* lipoma HMGIC fusion partner-like 3 \| \| 14.81 \| 29229814-29378390 (-) \| *Lmbr1,* limb region 1 \| \| 18.01 \| 35091490-35105766 (-) \| *Lrpap1,* low density lipoprotein receptor-related protein associated protein 1 \| \| 9.89 \| 21543527-21575900 (+) \| *Lrrc17,* leucine rich repeat containing 17 \| \| 20.29 \| 38220470-38234306 (+) \| *Lyar,* Ly1 antibody reactive clone \| \| 17.55 \| 33335572-33373294 (+) \| *Maea,* macrophage erythroblast attacher \| \| 19.26 \| 36806921-36830653 (-) \| *Man2b2,* mannosidase 2, alpha B2 \| \| 16.9 \| 30814641-30866106 (+) \| *Mapre3,* microtubule-associated protein, RP/EB family, member 3 \| \| 25.02 \| 45520221-45529276 (+) \| *Med28,* mediator of RNA polymerase II transcription, subunit 28 homolog (yeast) \| \| 17.9 \| 34633642-34637212 (-) \| *Mfsd10,* major facilitator superfamily domain containing 10 \| \| 12.35 \| 25271798-25498783 (-) \| *Mll3,* myeloid/lymphoid or mixed-lineage leukemia 3 \| \| 10.33 \| 23434441-23504235 (+) \| *Mll5,* myeloid/lymphoid or mixed-lineage leukemia 5 \| \| 15.04 \| 29473038-29478519 (-) \| *Mnx1,* motor neuron and pancreas homeobox 1 \| \| 17.12 \| 31140660-31158153 (-) \| *Mpv17,* MpV17 mitochondrial inner membrane protein \| \| 19.26 \| 36794867-36796772 (-) \| *Mrfap1,* Morf4 family associated protein 1 \| \| 17.27 \| 31596935-31701972 (+) \| *Mrpl33,* mitochondrial ribosomal protein L33 \| \| 17.94 \| 34915915-34923839 (+) \| *Msantd1,* Myb/SANT-like DNA-binding domain containing 1 \| \| 20.21 \| 37820485-37824583 (-) \| *Msx1,* homeobox, msh-like 1 \| \| 17.84 \| 34173887-34187720 (-) \| *Mxd4,* Max dimerization protein 4 \| \| 9.97 \| 21662901-21701396 (-) \| *Napepld,* N-acyl phosphatidylethanolamine phospholipase D \| \| 17.83 \| 33995984-34005916 (+) \| *Nat8l,* N-acetyltransferase 8-like \| \| 25.23 \| 45669922-45700544 (+) \| *Ncapg,* non-SMC condensin I complex, subunit G \| \| 17.65 \| 33430734-33433976 (-) \| *Nkx1-1,* NK1 transcription factor related, locus 1 (Drosophila) \| \| 22.58 \| 41761483-41764496 (-) \| *Nkx3-2,* NK3 homeobox 2 \| \| 15 \| 29434664-29453506 (+) \| *Nom1,* nucleolar protein with MIF4G domain 1 \| \| 17.91 \| 34638532-34660148 (-) \| *Nop14,* NOP14 nucleolar protein \| \| 11.32 \| 24364810-24384474 (+) \| *Nos3,* nitric oxide synthase 3, endothelial cell \| \| 17.27 \| 31240864-31251566 (+) \| *Nrbp1,* nuclear receptor binding protein 1 \| \| 20.21 \| 38137193-38159467 (-) \| *Nsg1,* neuron specific gene family member 1 \| \| 11.93 \| 24685815-24710555 (+) \| *Nub1,* negative regulator of ubiquitin-like proteins 1 \| \| 10.72 \| 24164963-24184013 (+) \| *Nupl2,* nucleoporin like 2 \| \| 10.07 \| 22486485-22550429 (-) \| *Orc5,* origin recognition complex, subunit 5 \| \| 16.9 \| 30905885-30907788 (-) \| *Ost4,* oligosaccharyltransferase 4 homolog (S. cerevisiae) \| \| 16.48 \| 30370076-30461932 (-) \| *Otof,* otoferlin \| \| 20.35 \| 38277404-38304217 (+) \| *Otop1,* otopetrin 1 \| \| 26.25 \| 48372392-48388756 (+) \| *Pacrgl,* PARK2 co-regulated-like \| \| 13.23 \| 27740667-27791339 (-) \| *Paxip1,* PAX interacting (with transcription-activation domain) protein 1 \| \| 28.63 \| 52741587-52769344 (+) \| *Pi4k2b,* phosphatidylinositol 4-kinase type 2 beta \| \| 17.33 \| 32736301-32785646 (-) \| *Pisd,* phosphatidylserine decarboxylase \| \| 17.33 \| 32232708-32366520 (+) \| *Plb1,* phospholipase B1 \| \| 9.97 \| 21737141-21757152 (+) \| *Pmpcb,* peptidase (mitochondrial processing) beta \| \| 17.83 \| 34007198-34169448 (-) \| *Poln,* DNA polymerase N \| \| 27.02 \| 51454249-51567725 (-) \| *Ppargc1a,* peroxisome proliferative activated receptor, gamma, coactivator 1 alpha \| \| 17.27 \| 31202668-31220545 (-) \| *Ppm1g,* protein phosphatase 1G (formerly 2C), magnesium-dependent, gamma isoform \| \| 17.33 \| 32458974-32517433 (+) \| *Ppp1cb,* protein phosphatase 1, catalytic subunit, beta isoform \| \| 19.28 \| 36868513-36955078 (+) \| *Ppp2r2c,* protein phosphatase 2 (formerly 2A), regulatory subunit B (PR 52), gamma isoform \| \| 16.9 \| 30950853-30960330 (-) \| *Preb,* prolactin regulatory element binding \| \| 11.93 \| 24862744-25100642 (-) \| *Prkag2,* protein kinase, AMP-activated, gamma 2 non-catalytic subunit \| \| 23.97 \| 43993622-44101736 (-) \| *Prom1,* prominin 1 \| \| 17.33 \| 32789218-32854256 (-) \| *Prr14l,* proline rich 14-like \| \| 19.14 \| 36204021-36206568 (+) \| *Psapl1,* prosaposin-like 1 \| \| 9.97 \| 21785283-21803787 (+) \| *Psmc2,* proteasome (prosome, macropain) 26S subunit, ATPase 2 \| \| 10.42 \| 23740648-23783711 (-) \| *Pus7,* pseudouridylate synthase 7 homolog (S. cerevisiae) \| \| 24.9 \| 45434032-45450235 (-) \| *Qdpr,* quinoid dihydropteridine reductase \| \| 22.58 \| 41624976-41708155 (-) \| *Rab28,* RAB28, member RAS oncogene family \| \| 17.27 \| 31624439-31697627 (-) \| *Rbks,* ribokinase \| \| 14.36 \| 28317121-28419244 (+) \| *Rbm33,* RNA binding motif protein 33 \| \| 29.37 \| 53590215-53657445 (+) \| *Rbpj,* recombination signal binding protein for immunoglobulin kappa J region \| \| 9.98 \| 21884454-22344702 (-) \| *Reln,* reelin \| \| 17.96 \| 34949445-35039644 (+) \| *Rgs12,* regulator of G-protein signaling 12 \| \| 11.93 \| 24802823-24842624 (-) \| *Rheb,* Ras homolog enriched in brain \| \| 10.45 \| 23787711-23820369 (+) \| *Rint1,* RAD50 interactor 1 \| \| 14.78 \| 29195992-29225524 (+) \| *Rnf32,* ring finger protein 32 \| \| 17.87 \| 34336390-34353430 (+) \| *Rnf4,* ring finger protein 4 \| \| 28.96 \| 53107084-53213452 (-) \| *Sel1l3,* sel-1 suppressor of lin-12-like 3 (C. elegans) \| \| 28.44 \| 52640087-52669729 (-) \| *Sepsecs,* Sep (O-phosphoserine) tRNA:Sec (selenocysteine) tRNA synthase \| \| 17.89 \| 34525838-34563625 (+) \| *Sh3bp2,* SH3-domain binding protein 2 \| \| 18.6 \| 35697180-35729276 (-) \| *Sh3tc1,* SH3 domain and tetratricopeptide repeats 1 \| \| 14.39 \| 28456815-28467101 (-) \| *Shh,* sonic hedgehog \| \| 17.83 \| 33634952-33652574 (-) \| *Slbp,* stem-loop binding protein \| \| 9.97 \| 21810655-21865604 (-) \| *Slc26a5,* solute carrier family 26, member 5 \| \| 20.43 \| 38349273-38503143 (-) \| *Slc2a9,* solute carrier family 2 (facilitated glucose transporter), member 9 \| \| 16.97 \| 31086106-31093615 (-) \| *Slc30a3,* solute carrier family 30 (zinc transporter), member 3 \| \| 28.92 \| 53038082-53071664 (+) \| *Slc34a2,* solute carrier family 34 (sodium phosphate), member 2 \| \| 16.74 \| 30647933-30659731 (+) \| *Slc35f6,* solute carrier family 35, member F6 \| \| 17.27 \| 31526995-31554042 (+) \| *Slc4a1ap,* solute carrier family 4 (anion exchanger), member 1, adaptor protein \| \| 11.74 \| 24423837-24440950 (+) \| *Slc4a2,* solute carrier family 4 (anion exchanger), member 2 \| \| 17.46 \| 33104219-33162872 (+) \| *Slc5a1,* solute carrier family 5 (sodium/glucose cotransporter), member 1 \| \| 16.93 \| 31036036-31048924 (-) \| *Slc5a6,* solute carrier family 5 (sodium-dependent vitamin transporter), member 6 \| \| 26.05 \| 47983155-48306282 (+) \| *Slit2,* slit homolog 2 (Drosophila) \| \| 11.93 \| 24590818-24602012 (-) \| *Smarcd3,* SWI/SNF related, matrix associated, actin dependent regulator of \| \|  \|  \| chromatin, subfamily d, member 3 \| \| 17.26 \| 31193227-31198900 (+) \| *Snx17,* sorting nexin 17 \| \| 27.92 \| 52363804-52369738 (+) \| *Sod3,* superoxide dismutase 3, extracellular \| \| 19.14 \| 36017180-36398139 (-) \| *Sorcs2,* sortilin-related VPS10 domain containing receptor 2 \| \| 12.44 \| 26032846-26039506 (-) \| *Speer4a,* spermatogenesis associated glutamate (E)-rich protein 4a \| \| 13.23 \| 27495639-27501438 (-) \| *Speer4b,* spermatogenesis associated glutamate (E)-rich protein 4b \| \| 17.5 \| 33213518-33218238 (-) \| *Spon2,* spondin 2, extracellular matrix protein \| \| 10.36 \| 23503356-23616571 (-) \| *Srpk2,* serine/arginine-rich protein specific kinase 2 \| \| 29.69 \| 53998417-54121057 (+) \| *Stim2,* stromal interaction molecule 2 \| \| 20.18 \| 37446828-37717153 (-) \| *Stk32b,* serine/threonine kinase 32B \| \| 20.21 \| 38038742-38137765 (+) \| *Stx18,* syntaxin 18 \| \| 17.27 \| 31514569-31527807 (-) \| *Supt7l,* suppressor of Ty 7-like \| \| 17.83 \| 33658128-33678995 (+) \| *Tacc3,* transforming, acidic coiled-coil containing protein 3 \| \| 19.14 \| 36473670-36484285 (-) \| *Tada2b,* transcriptional adaptor 2B \| \| 24.24 \| 44175164-44226606 (-) \| *Tapt1,* transmembrane anterior posterior transformation 1 \| \| 19.15 \| 36490604-36593276 (-) \| *Tbc1d14,* TBC1 domain family, member 14 \| \| 29.65 \| 53809627-53904380 (+) \| *Tbc1d19,* TBC1 domain family, member 19 \| \| 16.9 \| 30968677-30977018 (+) \| *Tcf23,* transcription factor 23 \| \| 20.33 \| 38260185-38269622 (+) \| *Tmem128,* transmembrane protein 128 \| \| 17.83 \| 33653216-33657977 (-) \| *Tmem129,* transmembrane protein 129 \| \| 16.9 \| 30869579-30879191 (+) \| *Tmem214,* transmembrane protein 214 \| \| 11.79 \| 24445458-24447854 (-) \| *Tmub1,* transmembrane and ubiquitin-like domain containing 1 \| \| 17.89 \| 34496087-34513997 (-) \| *Tnip2,* TNFAIP3 interacting protein 2 \| \| 10.48 \| 23838944-23844161 (-) \| *Tomm7,* translocase of outer mitochondrial membrane 7 homolog (yeast) \| \| 17.06 \| 31116712-31137630 (+) \| *Trim54,* tripartite motif-containing 54 \| \| 18.25 \| 35557206-35575070 (-) \| *Trmt44,* tRNA methyltransferase 44 \| \| 15.81 \| 30058202-30073617 (-) \| *Tyms,* thymidylate synthase \| \| 15.12 \| 29569242-29676077 (+) \| *Ube3c,* ubiquitin protein ligase E3C \| \| 17.11 \| 31138063-31138829 (-) \| *Ucn,* urocortin \| \| 17.6 \| 33378551-33419754 (+) \| *Uvssa,* UV stimulated scaffold protein A \| \| 20.63 \| 38526813-38561595 (-) \| *Wdr1,* WD repeat domain 1 \| \| 11.93 \| 24711738-24730727 (-) \| *Wdr86,* WD repeat domain 86 \| \| 19.46 \| 36966104-36988982 (-) \| *Wfs1,* Wolfram syndrome 1 homolog (human) \| \| 17.83 \| 33820725-33897975 (+) \| *Whsc1,* Wolf-Hirschhorn syndrome candidate 1 (human) \| \| 17.83 \| 33896936-33936413 (-) \| *Whsc2,* Wolf-Hirschhorn syndrome candidate 2 (human) \| \| 12.35 \| 25689812-25705825 (-) \| *Xrcc2,* X-ray repair complementing defective repair in Chinese hamster cells 2 \| \| 17.33 \| 32611171-32687066 (+) \| *Yes1,* Yamaguchi sarcoma viral (v-yes) oncogene homolog 1 \| \| 17.42 \| 33018816-33027966 (+) \| *Ywhah,* tyrosine 3-monooxygenase/tryptophan 5-monooxygenase \| \|  \|  \| activation protein, eta polypeptide \| \| 20.25 \| 38189735-38220457 (-) \| *Zbtb49,* zinc finger and BTB domain containing 49 \| \| 28.7 \| 52775409-52824665 (+) \| *Zcchc4,* zinc finger, CCHC domain containing 4 \| \| 17.27 \| 31452436-31481749 (+) \| *Zfp512,* zinc finger protein 512 \| \| 17.27 \| 31198981-31202303 (-) \| *Zfp513,* zinc finger protein 513 \| \| 20.77 \| 38668484-38684826 (-) \| *Zfp518b,* zinc finger protein 518B \| \| 17.85 \| 34194874-34288449 (-) \| *Zfyve28,* zinc finger, FYVE domain containing 28 \| |
| --- | --- | --- | --- | --- | --- | --- | --- | --- | --- | --- | --- | --- | --- | --- | --- | --- | --- | --- | --- | --- | --- | --- | --- | --- | --- | --- | --- | --- | --- | --- | --- | --- | --- | --- | --- | --- | --- | --- | --- | --- | --- | --- | --- | --- | --- | --- | --- | --- | --- | --- | --- | --- | --- | --- | --- | --- | --- | --- | --- | --- | --- | --- | --- | --- | --- | --- | --- | --- | --- | --- | --- | --- | --- | --- | --- | --- | --- | --- | --- | --- | --- | --- | --- | --- | --- | --- | --- | --- | --- | --- | --- | --- | --- | --- | --- | --- | --- | --- | --- | --- | --- | --- | --- | --- | --- | --- | --- | --- | --- | --- | --- | --- | --- | --- | --- | --- | --- | --- | --- | --- | --- | --- | --- | --- | --- | --- | --- | --- | --- | --- | --- | --- | --- | --- | --- | --- | --- | --- | --- | --- | --- | --- | --- | --- | --- | --- | --- | --- | --- | --- | --- | --- | --- | --- | --- | --- | --- | --- | --- | --- | --- | --- | --- | --- | --- | --- | --- | --- | --- | --- | --- | --- | --- | --- | --- | --- | --- | --- | --- | --- | --- | --- | --- | --- | --- | --- | --- | --- | --- | --- | --- | --- | --- | --- | --- | --- | --- | --- | --- | --- | --- | --- | --- | --- | --- | --- | --- | --- | --- | --- | --- | --- | --- | --- | --- | --- | --- | --- | --- | --- | --- | --- | --- | --- | --- | --- | --- | --- | --- | --- | --- | --- | --- | --- | --- | --- | --- | --- | --- | --- | --- | --- | --- | --- | --- | --- | --- | --- | --- | --- | --- | --- | --- | --- | --- | --- | --- | --- | --- | --- | --- | --- | --- | --- | --- | --- | --- | --- | --- | --- | --- | --- | --- | --- | --- | --- | --- | --- | --- | --- | --- | --- | --- | --- | --- | --- | --- | --- | --- | --- | --- | --- | --- | --- | --- | --- | --- | --- | --- | --- | --- | --- | --- | --- | --- | --- | --- | --- | --- | --- | --- | --- | --- | --- | --- | --- | --- | --- | --- | --- | --- | --- | --- | --- | --- | --- | --- | --- | --- | --- | --- | --- | --- | --- | --- | --- | --- | --- | --- | --- | --- | --- | --- | --- | --- | --- | --- | --- | --- | --- | --- | --- | --- | --- | --- | --- | --- | --- | --- | --- | --- | --- | --- | --- | --- | --- | --- | --- | --- | --- | --- | --- | --- | --- | --- | --- | --- | --- | --- | --- | --- | --- | --- | --- | --- | --- | --- | --- | --- | --- | --- | --- | --- | --- | --- | --- | --- | --- | --- | --- | --- | --- | --- | --- | --- | --- | --- | --- | --- | --- | --- | --- | --- | --- | --- | --- | --- | --- | --- | --- | --- | --- | --- | --- | --- | --- | --- | --- | --- | --- | --- | --- | --- | --- | --- | --- | --- | --- | --- | --- | --- | --- | --- | --- | --- | --- | --- | --- | --- | --- | --- | --- | --- | --- | --- | --- | --- | --- | --- | --- | --- | --- | --- | --- | --- | --- | --- | --- | --- | --- | --- | --- | --- | --- | --- | --- | --- | --- | --- | --- | --- | --- | --- | --- | --- | --- | --- | --- | --- | --- | --- | --- | --- | --- | --- | --- | --- | --- | --- | --- | --- | --- | --- | --- | --- | --- | --- | --- | --- | --- | --- | --- | --- | --- | --- | --- | --- | --- | --- | --- | --- | --- | --- | --- | --- | --- | --- | --- | --- | --- | --- | --- | --- | --- | --- | --- | --- | --- | --- | --- | --- | --- | --- | --- | --- | --- | --- | --- | --- | --- | --- | --- | --- | --- | --- | --- | --- | --- | --- | --- | --- | --- | --- | --- | --- | --- | --- | --- | --- | --- | --- | --- | --- | --- | --- | --- | --- | --- | --- | --- | --- | --- | --- | --- | --- | --- | --- | --- | --- | --- | --- | --- | --- | --- | --- | --- | --- | --- | --- | --- | --- | --- | --- | --- | --- | --- | --- | --- | --- | --- | --- | --- | --- | --- | --- | --- | --- | --- | --- | --- | --- | --- | --- | --- | --- | --- | --- | --- | --- | --- | --- | --- | --- | --- | --- | --- | --- | --- | --- | --- | --- | --- | --- | --- | --- | --- | --- | --- | --- | --- | --- | --- | --- | --- | --- | --- | --- | --- | --- | --- | --- | --- | --- | --- | --- | --- | --- | --- | --- | --- | --- | --- | --- | --- | --- | --- | --- | --- | --- | --- | --- | --- | --- | --- | --- | --- | --- | --- | --- | --- | --- | --- | --- | --- | --- | --- | --- | --- | --- | --- | --- | --- | --- | --- | --- | --- | --- | --- | --- | --- | --- | --- | --- | --- | --- | --- | --- | --- | --- | --- | --- | --- | --- | --- | --- | --- | --- | --- | --- | --- | --- | --- | --- | --- | --- | --- | --- | --- | --- | --- | --- | --- | --- | --- | --- | --- | --- | --- | --- | --- | --- | --- | --- | --- | --- | --- | --- | --- | --- | --- | --- | --- | --- | --- | --- | --- | --- | --- | --- | --- | --- | --- | --- | --- | --- | --- | --- | --- | --- | --- | --- | --- | --- | --- | --- | --- | --- | --- | --- | --- | --- | --- | --- | --- | --- | --- | --- | --- | --- | --- | --- | --- | --- | --- | --- | --- | --- |
| Genomic coordinates of genes were determined from the Mouse Genome Database (MGD), 2012. Genomic coordinates of genes were determined from the Mouse Genome Database (MGD), 2012. Eppig JT, *et al.* Nucleic Acids Res 2012; 40:D881-886. |
