# Supplementary material for: Genetic Dissection of Quantitative Trait Loci for Hemostasis and Thrombosis on Mouse Chromosomes 11 and 5 Using Congenic and Subcongenic Strains
Source: PLoS One. 2013 Oct 17;8(10):e77539. doi: 10.1371/journal.pone.0077539 (PMC3798288; doi:10.1371/journal.pone.0077539)
Supplement: Table S6 — Protein-coding Genes, Hmtb4, Chromosome 5, 108-126.8 Mbp. (DOCX) [file pone.0077539.s006.docx]

| **Table S6. Protein-coding Genes**  ***Hmtb4*, Chromosome 5, 108-126.8 Mbp** | | | |  |  |  |  |
| --- | --- | --- | --- | --- | --- | --- | --- |
| **cM** | **Genome Coordinates (Mbp)** | **Symbol, Name** |  |  |  |  |  |
| 60.63 | 120594305-120612589 (-) | 1110008J03Rik, RIKEN cDNA 1110008J03 gene | | |  |  |  |
| 55.99 | 114808196-114813976 (-) | 1500011B03Rik, RIKEN cDNA 1500011B03 gene | | |  |  |  |
| 55.55 | 113692422-113724772 (-) | 1700069L16Rik, RIKEN cDNA 1700069L16 gene | | |  |  |  |
| 55.99 | 114942158-114949783 (+) | 2210016L21Rik, RIKEN cDNA 2210016L21 gene | | |  |  |  |
| 58.32 | 118245227-118266093 (+) | 2410131K14Rik, RIKEN cDNA 2410131K14 gene | | |  |  |  |
| 55.99 | 114821935-114823468 (-) | 2610524H06Rik, RIKEN cDNA 2610524H06 gene | | |  |  |  |
| 63.52 | 124328089-124341844 (+) | 2810006K23Rik, RIKEN cDNA 2810006K23 gene | | |  |  |  |
| 55.38 | 113086323-113163351 (-) | 2900026A02Rik, RIKEN cDNA 2900026A02 gene | | |  |  |  |
| 55.99 | 114853664-114883659 (+) | 4930519G04Rik, RIKEN cDNA 4930519G04 gene | | |  |  |  |
| 53.26 | 109735990-109751886 (-) | 4930522L14Rik, RIKEN cDNA 4930522L14 gene | | |  |  |  |
| 53.25 | 109674545-109691041 (-) | 5430403G16Rik, RIKEN cDNA 5430403G16 gene | | |  |  |  |
| 64.24 | 125475814-125517412 (+) | *Aacs,* acetoacetyl-CoA synthetase | | |  |  |  |
| 63.36 | 124061530-124095798 (-) | *Abcb9,* ATP-binding cassette, sub-family B (MDR/TAP), member 9 | | | |  |  |
| 55.97 | 114146535-114250761 (+) | *Acacb,* acetyl-Coenzyme A carboxylase beta | | |  |  |  |
| 61.88 | 121621026-121660514 (-) | *Acad10,* acyl-Coenzyme A dehydrogenase family, member 10 | | | |  |  |
| 61.87 | 121598284-121618938 (-) | *Acad12,* acyl-Coenzyme A dehydrogenase family, member 12 | | | |  |  |
| 55.99 | 115110299-115119346 (-) | *Acads,* acyl-Coenzyme A dehydrogenase, short chain | | | |  |  |
| 61.84 | 121518576-121521695 (-) | *Adam1a,* a disintegrin and metallopeptidase domain 1a | | | |  |  |
| 61.83 | 121500098-121503435 (-) | *Adam1b,* a disintegrin and metallopeptidase domain 1b | | | |  |  |
| 55.29 | 112910478-113015514 (-) | *Adrbk2,* adrenergic receptor kinase, beta 2 | | |  |  |  |
| 61.86 | 121566027-121593824 (-) | *Aldh2,* aldehyde dehydrogenase 2, mitochondrial | | |  |  |  |
| 55.97 | 114123926-114128218 (-) | *Alkbh2,* alkB, alkylation repair homolog 2 (E. coli) | | |  |  |  |
| 62.58 | 122787469-122821339 (-) | *Anapc5,* anaphase-promoting complex subunit 5 | | |  |  |  |
| 62.36 | 122421693-122444912 (+) | *Anapc7,* anaphase promoting complex subunit 7 | | |  |  |  |
| 53.4 | 110231004-110256651 (+) | *Ankle2,* ankyrin repeat and LEM domain containing 2 | | | |  |  |
| 55.99 | 114774677-114806200 (+) | A*nkrd13a,* ankyrin repeat domain 13a | | |  |  |  |
| 63.39 | 124116089-124118196 (+) | *Arl6ip4,* ADP-ribosylation factor-like 6 interacting protein 4 | | | |  |  |
| 62.34 | 122391878-122406179 (+) | *Arpc3,* actin related protein 2/3 complex, subunit 3 | | |  |  |  |
| 54.7 | 112385448-112392213 (-) | *Asphd2,* aspartate beta-hydroxylase domain containing 2 | | | |  |  |
| 62.38 | 122453513-122502225 (-) | *Atp2a2,* ATPase, Ca++ transporting, cardiac muscle, slow twitch 2 | | | |  |  |
| 63.7 | 124629067-124722144 (+) | *Atp6v0a2,* ATPase, H+ transporting, lysosomal V0 subunit A2 | | | |  |  |
| 61.93 | 121711337-121816493 (+) | *Atxn2,* ataxin 2 |  |  |  |  |  |
| 55.54 | 113357294-113357821 (+) | *Aym1,* activator of yeast meiotic promoters 1 | | |  |  |  |
| 63.02 | 123510460-123511882 (+) | *B3gnt4,* UDP-GlcNAc:betaGal beta-1,3-N-acetylglucosaminyltransferase 4 | | | | |  |
| 62.92 | 123343834-123374082 (+) | *Bcl7a,* B cell CLL/lymphoma 7A | |  |  |  |  |
| 61.9 | 121660563-121687248 (+) | *Brap,* BRCA1 associated protein | |  |  |  |  |
| 64.21 | 125441568-125460872 (+) | *Bri3bp,* Bri3 binding protein | |  |  |  |  |
| 55.99 | 115168689-115194381 (-) | *Cabp1,* calcium binding protein 1 | | |  |  |  |
| 62.55 | 122731173-122779410 (-) | *Camkk2,* calcium/calmodulin-dependent protein kinase kinase 2, beta | | | | |  |
| 56.12 | 116124641-116288985 (-) | *Ccdc60,* coiled-coil domain containing 60 | | |  |  |  |
| 63.28 | 123927418-123968909 (+) | *Ccdc62,* coiled-coil domain containing 62 | | |  |  |  |
| 62.17 | 122108040-122138957 (-) | *Ccdc63,* coiled-coil domain containing 63 | | |  |  |  |
| 56.1 | 115648175-115731621 (-) | Ccdc64, coiled-coil domain containing 64 | | |  |  |  |
| 63.85 | 124834418-124862424 (-) | *Ccdc92,* coiled-coil domain containing 92 | | |  |  |  |
| 63.53 | 124345417-124354671 (-) | *Cdk2ap1,* CDK2 (cyclin-dependent kinase 2)-associated protein 1 | | | |  |  |
| 53.73 | 110840017-110874133 (+) | *Chek2,* checkpoint kinase 2 | |  |  |  |  |
| 53.32 | 110135840-110171972 (+) | *Chfr,* checkpoint with forkhead and ring finger domains | | | |  |  |
| 56.1 | 115845278-116008947 (+) | *Cit,* citron |  |  |  |  |  |
| 63.06 | 123577795-123684618 (-) | *Clip1,* CAP-GLY domain containing linker protein 1 | | | |  |  |
| 55.55 | 113612354-113650426 (-) | *Cmklr1,* chemokine-like receptor 1 | | |  |  |  |
| 56.03 | 115279666-115296972 (+) | *Coq5,* coenzyme Q5 homolog, methyltransferase (yeast) | | | |  |  |
| 55.64 | 113842436-113908758 (-) | *Coro1c,* coronin, actin binding protein 1C | | |  |  |  |
| 56.06 | 115345642-115348981 (-) | *Cox6a1,* cytochrome c oxidase subunit VIa polypeptide 1 | | | |  |  |
| 53.24 | 109554711-109558993 (-) | *Crlf2,* cytokine receptor-like factor 2 | | |  |  |  |
| 54.62 | 112246493-112252518 (-) | *Cryba4,* crystallin, beta A4 | |  |  |  |  |
| 54.63 | 112255815-112269585 (+) | *Crybb1,* crystallin, beta B1 | |  |  |  |  |
| 55.38 | 113058258-113070117 (-) | *Crybb2,* crystallin, beta B2 | |  |  |  |  |
| 55.38 | 113075839-113081584 (-) | *Crybb3,* crystallin, beta B3 | |  |  |  |  |
| 62.02 | 121857963-122050102 (-) | *Cux2,* cut-like homeobox 2 | |  |  |  |  |
| 55.93 | 114003703-114025449 (+) | *Dao,* D-amino acid oxidase | |  |  |  |  |
| 53.69 | 110653451-110660496 (+) | *Ddx51,* DEAD (Asp-Glu-Ala-Asp) box polypeptide 51 | | | |  |  |
| 60.63 | 120613130-120628590 (+) | *Ddx54,* DEAD (Asp-Glu-Ala-Asp) box polypeptide 54 | | | |  |  |
| 63.66 | 124552864-124569660 (+) | *Ddx55,* DEAD (Asp-Glu-Ala-Asp) box polypeptide 55 | | | |  |  |
| 63.26 | 123907175-123928832 (+) | *Denr,* density-regulated protein | |  |  |  |  |
| 64.2 | 125414406-125434048 (-) | *Dhx37,* DEAH (Asp-Glu-Ala-His) box polypeptide 37 | | | |  |  |
| 63.02 | 123509765-123524176 (-) | *Diablo,* diablo homolog (Drosophila) | | |  |  |  |
| 63.78 | 124731391-124834303 (+) | *Dnahc10,* dynein, axonemal, heavy chain 10 | | |  |  |  |
| 60.64 | 120680202-120711927 (-) | *Dtx1,* deltex 1 homolog (Drosophila) | | |  |  |  |
| 56.04 | 115297110-115300999 (-) | *Dynll1,* dynein light chain LC8-type 1 | | |  |  |  |
| 63.67 | 124570213-124579131 (-) | *Eif2b1,* eukaryotic translation initiation factor 2B, subunit 1 (alpha) | | | |  |  |
| 53.69 | 110664373-110770717 (-) | *Ep400,* E1A binding protein p400 | | |  |  |  |
| 61.8 | 121428590-121452506 (-) | *Erp29,* endoplasmic reticulum protein 29 | | |  |  |  |
| 63.95 | 125003447-125012547 (+) | *Fam101a,* family with sequence similarity 101, member A | | | |  |  |
| 62.01 | 121849028-121854599 (+) | *Fam109a,* family with sequence similarity 109, member A | | | |  |  |
| 62.33 | 122364580-122372364 (-) | *Fam216a,* family with sequence similarity 216, member A | | | |  |  |
| 55.99 | 114568016-114613220 (+) | *Fam222a,* family with sequence similarity 222, member A | | | |  |  |
| 53.51 | 110361754-110448503 (-) | *Fbrsl1,* fibrosin-like 1 | |  |  |  |  |
| 57.73 | 117976770-118010191 (+) | *Fbxo21,* F-box protein 21 | |  |  |  |  |
| 57.92 | 118064981-118155458 (-) | *Fbxw8,* F-box and WD-40 domain protein 8 | | |  |  |  |
| 55.55 | 113735782-113740600 (+) | *Ficd,* FIC domain containing | |  |  |  |  |
| 55.99 | 114254164-114273807 (-) | *Foxn4,* forkhead box N4 | |  |  |  |  |
| 53.66 | 110544345-110621382 (+) | *Galnt9,* UDP-N-acetyl-alpha-D-galactosamine:polypeptide | | | |  |  |
|  |  | N-acetylgalactosaminyltransferase 9 | | |  |  |  |
| 56.05 | 115333242-115341178 (-) | *Gatc,* glutamyl-tRNA(Gln) amidotransferase, subunit C homolog (bacterial) | | | | |  |
| 56.1 | 115565254-115622656 (+) | *Gcn1l1,* GCN1 general control of amino-acid synthesis 1-like 1 (yeast) | | | | |  |
| 55.99 | 114727407-114775517 (-) | *Git2,* G protein-coupled receptor kinase-interactor 2 | | | |  |  |
| 55.99 | 114669403-114690984 (-) | *Gltp,* glycolipid transfer protein | |  |  |  |  |
| 64.18 | 125389322-125390554 (+) | G*m10382,* predicted gene 10382 | | |  |  |  |
| 56.88 | 117318745-117319242 (-) | *Gm10399,* predicted gene 10399 | | |  |  |  |
| 55.99 | 114842349-114842862 (-) | *Gm13821,* predicted gene 13821 | | |  |  |  |
| 55.99 | 115102922-115110268 (-) | *Gm13826,* predicted gene 13826 | | |  |  |  |
| 53.28 | 109923400-109943936 (+) | *Gm15446,* predicted gene 15446 | | |  |  |  |
| 61.72 | 121220219-121368577 (+) | *Gm15800,* predicted gene 15800 | | |  |  |  |
| 53.3 | 110046232-110046486 (-) | *Gm17655,* predicted gene, 17655 | | |  |  |  |
| 63.15 | 123725183-123725359 (+) | *Gm19601,* predicted gene, 19601 | | |  |  |  |
| 55.99 | 114809036-114823460 (-) | *Gm20499,* predicted gene 20499 | | |  |  |  |
| 64.46 | 125847949-125848740 (-) | *Gm4868,* predicted gene 4868 | |  |  |  |  |
| 60.4 | 119803739-119831785 (-) | *Gm5563,* predicted gene 5563 | |  |  |  |  |
| 54.69 | 112353801-112356033 (-) | *Gm6583,* predicted gene 6583 | |  |  |  |  |
| 54.72 | 112449426-112451738 (+) | *Gm6588,* predicted gene 6588 | |  |  |  |  |
| 57.92 | 118065360-118069710 (+) | G*m9754,* predicted gene 9754 | |  |  |  |  |
| 55.99 | 114855758-114858682 (-) | *Gm9936,* predicted gene 9936 | |  |  |  |  |
| 53.36 | 110176701-110226470 (+) | *Golga3,* golgi autoantigen, golgin subfamily a, 3 | | |  |  |  |
| 62.33 | 122371876-122382902 (+) | *Gpn3,* GPN-loop GTPase 3 | |  |  |  |  |
| 63.24 | 123876736-123880020 (-) | *Gpr81,* G protein-coupled receptor 81 | | |  |  |  |
| 63.67 | 124579140-124597680 (+) | *Gtf2h3,* general transcription factor IIH, polypeptide 3 | | | |  |  |
| 53.3 | 110103975-110108197 (-) | *Gtpbp6,* GTP binding protein 6 (putative) | | |  |  |  |
| 63.3 | 123973628-124003211 (+) | *Hip1r,* huntingtin interacting protein 1 related | | |  |  |  |
| 55.99 | 114948361-114971067 (-) | *Hnf1a,* HNF1 homeobox A | |  |  |  |  |
| 62.82 | 123171807-123182725 (-) | *Hpd,* 4-hydroxyphenylpyruvic acid dioxygenase | | |  |  |  |
| 54.69 | 112343083-112378414 (+) | *Hps4,* Hermansky-Pudlak syndrome 4 homolog (human) | | | |  |  |
| 58.16 | 118169764-118189478 (+) | *Hrk,* harakiri, BCL2 interacting protein (contains only BH3 domain) | | | |  |  |
| 53.72 | 110829070-110839777 (-) | *Hscb,* HscB iron-sulfur cluster co-chaperone homolog (E. coli) | | | |  |  |
| 56.41 | 116408491-116422864 (-) | *Hspb8,* heat shock protein 8 | |  |  |  |  |
| 62.23 | 122206804-122242297 (+) | *Hvcn1,* hydrogen voltage-gated channel 1 | | |  |  |  |
| 62.44 | 122550204-122614518 (-) | *Ift81,* intraflagellar transport 81 | |  |  |  |  |
| 63 | 123480153-123482101 (-) | *Il31,* interleukin 31 | |  |  |  |  |
| 60.63 | 120589016-120607114 (+) | *Iqcd,* IQ motif containing D | |  |  |  |  |
| 55.55 | 113772748-113778288 (+) | *Iscu,* IscU iron-sulfur cluster scaffold homolog (E. coli) | | | |  |  |
| 55.99 | 114363567-114380515 (-) | *Kctd10,* potassium channel tetramerisation domain containing 10 | | | |  |  |
| 62.63 | 122870668-123015080 (-) | *Kdm2b,* lysine (K)-specific demethylase 2B | | |  |  |  |
| 63.17 | 123749726-123821593 (+) | *Kntc1,* kinetochore associated 1 | |  |  |  |  |
| 56.88 | 117414000-117555942 (+) | *Ksr2,* kinase suppressor of ras 2 | |  |  |  |  |
| 60.62 | 120431770-120441455 (+) | *Lhx5,* LIM homeobox protein 5 | |  |  |  |  |
| 53.5 | 110354096-110356087 (+) | *Lrcol1,* leucine rich colipase-like 1 | | |  |  |  |
| 63.01 | 123489325-123508205 (+) | *Lrrc43,* leucine rich repeat containing 43 | | |  |  |  |
| 61.84 | 121518613-121545905 (-) | *Mapkapk5,* MAP kinase-activated protein kinase 5 | | |  |  |  |
| 59.1 | 118560719-118765435 (+) | *Med13l,* mediator complex subunit 13-like | | |  |  |  |
| 55.99 | 115142981-115158179 (-) | *Mlec,* malectin |  |  |  |  |  |
| 62.95 | 123394798-123457932 (+) | *Mlxip,* MLX interacting protein | |  |  |  |  |
| 55.99 | 114431034-114444059 (-) | *Mmab,* methylmalonic aciduria (cobalamin deficiency) type B homolog (human) | | | | |  |
| 53.86 | 111417362-111457033 (+) | *Mn1,* meningioma 1 | |  |  |  |  |
| 62.73 | 123035769-123047016 (-) | *Morn3,* MORN repeat containing 3 | | |  |  |  |
| 63.47 | 124250959-124327972 (-) | *Mphosph9,* M-phase phosphoprotein 9 | | |  |  |  |
| 56.09 | 115429599-115455698 (+) | *Msi1,* musashi RNA-binding protein 1 | | |  |  |  |
| 55.99 | 114444269-114460591 (+) | *Mvk,* mevalonate kinase | |  |  |  |  |
| 62.16 | 122100951-122113472 (+) | *Myl2,* myosin, light polypeptide 2, regulatory, cardiac, slow | | | |  |  |
| 54.8 | 112688876-112896362 (-) | *Myo18b,* myosin XVIIIb | |  |  |  |  |
| 55.99 | 114314941-114364576 (+) | *Myo1h,* myosin 1H | |  |  |  |  |
| 61.79 | 121397936-121444378 (+) | *Naa25,* N(alpha)-acetyltransferase 25, NatB auxiliary subunit | | | |  |  |
| 63.96 | 125017153-125179219 (-) | *Ncor2,* nuclear receptor co-repressor 2 | | |  |  |  |
| 63.24 | 123863570-123865516 (-) | *Niacr1,* niacin receptor 1 | |  |  |  |  |
| 53.69 | 110648418-110653417 (-) | *Noc4l,* nucleolar complex associated 4 homolog (S. cerevisiae) | | | |  |  |
| 57.29 | 117781032-117958840 (+) | *Nos1,* nitric oxide synthase 1, neuronal | | |  |  |  |
| 60.65 | 120896256-120907521 (-) | *Oas1a,* 2'-5' oligoadenylate synthetase 1A | | |  |  |  |
| 60.64 | 120812635-120824158 (+) | *Oas1b,* 2'-5' oligoadenylate synthetase 1B | | |  |  |  |
| 60.64 | 120800194-120812514 (-) | O*as1c,* 2'-5' oligoadenylate synthetase 1C | | |  |  |  |
| 60.87 | 120914536-120921652 (+) | *Oas1d,* 2'-5' oligoadenylate synthetase 1D | | |  |  |  |
| 60.64 | 120786312-120795530 (-) | *Oas1e,* 2'-5' oligoadenylate synthetase 1E | | |  |  |  |
| 60.64 | 120847367-120857986 (+) | *Oas1f,* 2'-5' oligoadenylate synthetase 1F | | |  |  |  |
| 60.65 | 120876142-120887613 (-) | *Oas1g,* 2'-5' oligoadenylate synthetase 1G | | |  |  |  |
| 60.64 | 120861421-120873512 (+) | *Oas1h,* 2'-5' oligoadenylate synthetase 1H | | |  |  |  |
| 60.64 | 120730333-120749853 (-) | *Oas2,* 2'-5' oligoadenylate synthetase 2 | | |  |  |  |
| 60.64 | 120753098-120777661 (-) | *Oas3,* 2'-5' oligoadenylate synthetase 3 | | |  |  |  |
| 55.99 | 114923240-114937915 (+) | *Oasl1,* 2'-5' oligoadenylate synthetase-like 1 | | |  |  |  |
| 55.99 | 114896936-114912234 (+) | *Oasl2,* 2'-5' oligoadenylate synthetase-like 2 | | |  |  |  |
| 63.39 | 124112297-124115483 (+) | *Ogfod2,* 2-oxoglutarate and iron-dependent oxygenase domain containing 2 | | | | |  |
| 62.72 | 123015074-123030450 (+) | *Orai1,* ORAI calcium release-activated calcium modulator 1 | | | |  |  |
| 53.49 | 110339812-110343035 (-) | *P2rx2,* purinergic receptor P2X, ligand-gated ion channel, 2 | | | |  |  |
| 62.53 | 122707584-122729738 (+) | *P2rx4,* purinergic receptor P2X, ligand-gated ion channel 4 | | | |  |  |
| 62.5 | 122643911-122691432 (+) | *P2rx7,* purinergic receptor P2X, ligand-gated ion channel, 7 | | | |  |  |
| 56.88 | 117282651-117287625 (-) | *Pebp1,* phosphatidylethanolamine binding protein 1 | | | |  |  |
| 53.43 | 110259130-110269913 (-) | *Pgam5,* phosphoglycerate mutase family member 5 | | | |  |  |
| 53.79 | 111330763-111388359 (+) | *Pitpnb,* phosphatidylinositol transfer protein, beta | | |  |  |  |
| 63.39 | 124118690-124249760 (-) | *Pitpnm2,* phosphatidylinositol transfer protein, membrane-associated 2 | | | | |  |
| 56.1 | 115466262-115474722 (+) | *Pla2g1b,* phospholipase A2, group IB, pancreas | | |  |  |  |
| 60.63 | 120483282-120503625 (-) | *Plbd2,* phospholipase B domain containing 2 | | |  |  |  |
| 53.3 | 110099969-110105953 (+) | P*lcxd1,* phosphatidylinositol-specific phospholipase C, X domain containing 1 | | | | |  |
| 53.45 | 110286306-110337474 (+) | *Pole,* polymerase (DNA directed), epsilon | | |  |  |  |
| 56.01 | 115235836-115245351 (+) | *Pop5,* processing of precursor 5, ribonuclease P/MRP family (S. cerevisiae) | | | | |  |
| 62.2 | 122158278-122175273 (+) | *Ppp1cc,* protein phosphatase 1, catalytic subunit, gamma isoform | | | |  |  |
| 62.28 | 122284365-122324281 (+) | *Pptc7,* PTC7 protein phosphatase homolog (S. cerevisiae) | | | |  |  |
| 56.1 | 116013586-116024508 (-) | P*rkab1,* protein kinase, AMP-activated, beta 1 non-catalytic subunit | | | |  |  |
| 62.85 | 123228190-123250131 (+) | *Psmd9,* proteasome (prosome, macropain) 26S subunit, non-ATPase, 9 | | | | |  |
| 61.72 | 121130533-121191397 (-) | *Ptpn11,* protein tyrosine phosphatase, non-receptor type 11 | | | |  |  |
| 53.7 | 110773667-110780659 (-) | *Pus1,* pseudouridine synthase 1 | |  |  |  |  |
| 53.44 | 110274282-110286186 (-) | *Pxmp2,* peroxisomal membrane protein 2 | | |  |  |  |
| 56.1 | 115506676-115555987 (+) | *Pxn,* paxillin |  |  |  |  |  |
| 56.1 | 115631929-115647736 (+) | *Rab35,* RAB35, member RAS oncogene family | | |  |  |  |
| 62.3 | 122323223-122354233 (-) | *Rad9b,* RAD9 homolog B | |  |  |  |  |
| 60.63 | 120648812-120679597 (+) | *Rasal1,* RAS protein activator like 1 (GAP1 like) | | |  |  |  |
| 60.58 | 120116510-120197979 (+) | *Rbm19,* RNA binding motif protein 19 | | |  |  |  |
| 56.88 | 117378103-117389047 (-) | *Rfc5,* replication factor C (activator 1) 5 | | |  |  |  |
| 62.77 | 123103044-123132692 (-) | *Rhof,* ras homolog gene family, member f | | |  |  |  |
| 63.62 | 124493082-124531391 (-) | *Rilpl1,* Rab interacting lysosomal protein-like 1 | | |  |  |  |
| 63.6 | 124463265-124478366 (-) | *Rilpl2,* Rab interacting lysosomal protein-like 2 | | |  |  |  |
| 56.01 | 115241412-115272898 (-) | *Rnf10,* ring finger protein 10 | |  |  |  |  |
| 62.62 | 122850188-122868945 (+) | *Rnf34,* ring finger protein 34 | |  |  |  |  |
| 58.2 | 118190736-118245116 (-) | *Rnft2,* ring finger protein, transmembrane 2 | | |  |  |  |
| 61.17 | 120940502-121009518 (-) | *Rph3a,* rabphilin 3A | |  |  |  |  |
| 61.72 | 121204481-121209241 (+) | *Rpl6,* ribosomal protein L6 | |  |  |  |  |
| 56.1 | 115559467-115563727 (+) | *Rplp0,* ribosomal protein, large, P0 | | |  |  |  |
| 63.15 | 123728430-123749412 (-) | *Rsrc2,* arginine/serine-rich coiled-coil 2 | | |  |  |  |
| 55.55 | 113742446-113771649 (-) | *Sart3,* squamous cell carcinoma antigen recognized by T cells 3 | | | |  |  |
| 63.54 | 124368702-124425914 (-) | *Sbno1,* sno, strawberry notch homolog 1 (Drosophila) | | | |  |  |
| 64.11 | 125277087-125341094 (-) | *Scarb1,* scavenger receptor class B, member 1 | | |  |  |  |
| 60.63 | 120476531-120483932 (+) | *Sds,* serine dehydratase | |  |  |  |  |
| 60.62 | 120458186-120472810 (-) | *Sdsl,* serine dehydratase-like | |  |  |  |  |
| 55.59 | 113817798-113830501 (-) | *Selplg,* selectin, platelet (p-selectin) ligand | | |  |  |  |
| 62.8 | 123142193-123167435 (+) | *Setd1b,* SET domain containing 1B | | |  |  |  |
| 63.59 | 124439930-124462308 (+) | *Setd8,* SET domain containing (lysine methyltransferase) 8 | | | |  |  |
| 54.72 | 112419151-112577198 (-) | *Sez6l,* seizure related 6 homolog like | | |  |  |  |
| 55.45 | 113243220-113310786 (-) | *Sgsm1,* small G protein signaling modulator 1 | | |  |  |  |
| 61.99 | 121815488-121836859 (-) | *Sh2b3,* SH2B adaptor protein 3 | |  |  |  |  |
| 56.1 | 115478010-115484725 (-) | *Sirt4,* sirtuin 4 (silent mating type information regulation 2 homolog) 4 (S. cerevisiae) | | | | |  |
| 60.63 | 120511168-120534024 (+) | *Slc24a6,* solute carrier family 24 (sodium/potassium/calcium exchanger), member 6 | | | | |  |
| 63.61 | 124483134-124491124 (+) | *Snrnp35,* small nuclear ribonucleoprotein 35 (U11/U12) | | | |  |  |
| 55.99 | 115011137-115098790 (+) | *Sppl3,* signal peptide peptidase 3 | | |  |  |  |
| 54.69 | 112337392-112343040 (-) | *Srrd,* SRR1 domain containing | |  |  |  |  |
| 56.45 | 116439272-116467860 (-) | *Srrm4,* serine/arginine repetitive matrix 4 | | |  |  |  |
| 56.05 | 115327177-115333080 (+) | *Srsf9,* serine/arginine-rich splicing factor 9 | | |  |  |  |
| 55.82 | 113937094-113993894 (-) | *Ssh1,* slingshot homolog 1 (Drosophila) | | |  |  |  |
| 56.88 | 117091682-117115993 (-) | *Suds3,* suppressor of defective silencing 3 homolog (S. cerevisiae) | | | |  |  |
| 55.94 | 114026910-114091570 (-) | *Svop,* SV2 related protein | |  |  |  |  |
| 56.88 | 117120129-117275219 (+) | *Taok3,* TAO kinase 3 | |  |  |  |  |
| 60.34 | 119670862-119684724 (+) | *Tbx3,* T-box 3 |  |  |  |  |  |
| 60.42 | 119834663-119885218 (+) | *Tbx5,* T-box 5 |  |  |  |  |  |
| 55.99 | 114707760-114722327 (+) | *Tchp,* trichoplein, keratin filament binding | | |  |  |  |
| 62.25 | 122241287-122264460 (-) | *Tctn1,* tectonic family member 1 | |  |  |  |  |
| 63.68 | 124598749-124619108 (+) | *Tctn2,* tectonic family member 2 | |  |  |  |  |
| 57.84 | 118027743-118061878 (+) | *Tesc,* tescalcin |  |  |  |  |  |
| 54.69 | 112326358-112338073 (+) | *Tfip11,* tuftelin interacting protein 11 | | |  |  |  |
| 63.65 | 124540695-124550506 (+) | *Tmed2,* transmembrane emp24 domain trafficking protein 2 | | | |  |  |
| 61.81 | 121451893-121524183 (+) | *Tmem116,* transmembrane protein 116 | | |  |  |  |
| 55.55 | 113793729-113800516 (-) | *Tmem119,* transmembrane protein 119 | | |  |  |  |
| 62.75 | 123068415-123117749 (+) | *Tmem120b,* transmembrane protein 120B | | |  |  |  |
| 64.27 | 125532418-125792583 (+) | *Tmem132b,* transmembrane protein 132B | | |  |  |  |
| 55.43 | 113226909-113239263 (+) | *Tmem211,* transmembrane protein 211 | | |  |  |  |
| 56.1 | 116038755-116083244 (-) | *Tmem233,* transmembrane protein 233 | | |  |  |  |
| 60.63 | 120534160-120588613 (-) | *Tpcn1,* two pore channel 1 | |  |  |  |  |
| 54.65 | 112276691-112315361 (+) | *Tpst2,* protein-tyrosine sulfotransferase 2 | | |  |  |  |
| 61.78 | 121371725-121385632 (-) | *Trafd1,* TRAF type zinc finger domain containing 1 | | |  |  |  |
| 56.06 | 115341225-115343569 (+) | *Triap1,* TP53 regulated inhibitor of apoptosis 1 | | |  |  |  |
| 55.99 | 114622152-114658421 (-) | *Trpv4,* transient receptor potential cation channel, subfamily V, member 4 | | | | |  |
| 53.74 | 110879803-111289780 (+) | *Ttc28,* tetratricopeptide repeat domain 28 | | |  |  |  |
| 64.18 | 125385965-125390202 (-) | *Ubc,* ubiquitin C |  |  |  |  |  |
| 55.99 | 114380628-114421168 (+) | *Ube3b,* ubiquitin protein ligase E3B | | |  |  |  |
| 53.7 | 110784489-110810081 (-) | *Ulk1,* unc-51 like kinase 1 | |  |  |  |  |
| 55.99 | 115122550-115134975 (-) | *Unc119b,* unc-119 homolog B (C. elegans) | | |  |  |  |
| 55.97 | 114130386-114139323 (+) | *Ung,* uracil DNA glycosylase | |  |  |  |  |
| 55.96 | 114100333-114123506 (+) | *Usp30,* ubiquitin specific peptidase 30 | | |  |  |  |
| 53.24 | 108993412-109006471 (-) | *Vmn2r10,* vomeronasal 2, receptor 10 | | |  |  |  |
| 53.24 | 109046873-109059452 (-) | *Vmn2r11,* vomeronasal 2, receptor 11 | | |  |  |  |
| 53.24 | 109085849-109097864 (-) | *Vmn2r12,* vomeronasal 2, receptor 12 | | |  |  |  |
| 53.24 | 109156068-109192107 (-) | *Vmn2r13,* vomeronasal 2, receptor 13 | | |  |  |  |
| 53.24 | 109215502-109224622 (-) | *Vmn2r14,* vomeronasal 2, receptor 14 | | |  |  |  |
| 53.24 | 109286269-109297556 (-) | *Vmn2r15,* vomeronasal 2, receptor 15 | | |  |  |  |
| 53.24 | 109330381-109364481 (+) | *Vmn2r16,* vomeronasal 2, receptor 16 | | |  |  |  |
| 53.24 | 109420013-109453387 (+) | *Vmn2r17,* vomeronasal 2, receptor 17 | | |  |  |  |
| 62.32 | 122354369-122364984 (+) | *Vps29,* vacuolar protein sorting 29 (S. pombe) | | |  |  |  |
| 63.03 | 123528764-123573015 (-) | *Vps33a,* vacuolar protein sorting 33A (yeast) | | |  |  |  |
| 63.32 | 124004641-124032260 (-) | *Vps37b,* vacuolar protein sorting 37B (yeast) | | |  |  |  |
| 56.88 | 117319266-117355005 (+) | *Vsig10,* V-set and immunoglobulin domain containing 10 | | | |  |  |
| 62.86 | 123252102-123327484 (+) | *Wdr66,* WD repeat domain 66 | |  |  |  |  |
| 56.88 | 117357304-117378601 (+) | *Wsb2,* WD repeat and SOCS box-containing 2 | | |  |  |  |
| 55.55 | 113490752-113490940 (+) | *Wscd2,* WSC domain containing 2 | | |  |  |  |
| 63.14 | 123698302-123721044 (-) | *Zcchc8,* zinc finger, CCHC domain containing 8 | | |  |  |  |
| 53.3 | 110110092-110129794 (+) | *Zfp605,* zinc finger protein 605 | |  |  |  |  |
| 63.86 | 124862691-124902693 (+) | *Zfp664,* zinc finger protein 664 | |  |  |  |  |
| 53.29 | 109996521-110010411 (+) | *Zfp932,* zinc finger protein 932 | |  |  |  |  |

Genomic coordinates of genes were determined from the Mouse Genome Database (MGD), 2012. Eppig JT, *et al.* Nucleic Acids Res 2012; 40:D881-886.
