# Supplementary material for: Genetic Dissection of Quantitative Trait Loci for Hemostasis and Thrombosis on Mouse Chromosomes 11 and 5 Using Congenic and Subcongenic Strains
Source: PLoS One. 2013 Oct 17;8(10):e77539. doi: 10.1371/journal.pone.0077539 (PMC3798288; doi:10.1371/journal.pone.0077539)
Supplement: Table S7 — Protein-coding Genes Non-overlapping Region 4A-2, Hmtb11, Chromosome 5, 54.3-64.6 Mbp. (DOCX) [file pone.0077539.s007.docx]

| **Table S7. Protein-coding Genes Nonoverlapping Region 4A-2**  **Hmtb11, Chromosome 5, 54.3-64.6 Mbp** | | |
| --- | --- | --- |
| **cM** | **Genome Coordinates** | **Symbol, Name** |
| 32.8 | 63812495-63899619 (+) | *0610040J01Rik,* RIKEN cDNA 0610040J01 gene |
| 32.8 | 63649103-63810546 (+) | *3110047P20Rik,* RIKEN cDNA 3110047P20 gene |
| 32.4 | 62602445-62766198 (-) | *Arap2,*ArfGAP with RhoGAP domain, ankyrin repeat and PH domain 2 |
| 31.99 | 61808884-61810425 (+) | *G6pd2,* glucose-6-phosphate dehydrogenase 2 |
| 32.04 | 62813823-62888308 (+) | *Gm17384,* predicted gene, 17384 |
| 32.8 | 63649950-63650264 (-) | *Gm9954,* predicted gene, 9954 |
| 31.65 | 57718021-58129351 (+) | *Pcdh7,* protocadherin 7 |
| 32.8 | 64092936-64128355 (+) | *Pgm1,* phosphoglucomutase 1 |
| 32.8 | 63908896-63968905 (-) | *Rell1,* RELT-like 1 |
| 32.8 | 64159451-64351482 (+) | *Tbc1d1,* TBC1 domain family, member 1 |

Genomic coordinates of genes were determined from the Mouse Genome Database (MGD), 2012. Genomic coordinates of genes were determined from the Mouse Genome Database (MGD), 2012. Eppig JT, *et al.* Nucleic Acids Res 2012; 40:D881-886.
