# Supplementary material for: Genetic Dissection of Quantitative Trait Loci for Hemostasis and Thrombosis on Mouse Chromosomes 11 and 5 Using Congenic and Subcongenic Strains
Source: PLoS One. 2013 Oct 17;8(10):e77539. doi: 10.1371/journal.pone.0077539 (PMC3798288; doi:10.1371/journal.pone.0077539)
Supplement: Table S8 — Protein-coding Genes, Hmtb11, Protein-coding Genes, Chromosome 5, 0-21.4. (DOCX) [file pone.0077539.s008.docx]

| **Table S8. Protein-coding Genes**  ***Hmtb11,* Protein-coding Genes, Chromosome 5, 0-21.4** | | |
| --- | --- | --- |
| **cM** | **Genome Coordinates** | **Symbol, Name** |
| 2.89 cM | 5437827-5479143 (-) | *1700015F17Rik,* RIKEN cDNA 1700015F17 gene |
| 2.24 cM | 3571566-3584356 (+) | *1700109H08Rik,* RIKEN cDNA 1700109H08 gene |
| 3.37 cM | 7304125-7311491 (+) | *4921511H03Rik,* RIKEN cDNA 4921511H03 gene |
| 2.26 cM | 3657004-3680325 (+) | *4930511M11Rik,* RIKEN cDNA 4930511M11 gene |
| 6.51 cM | 15652286-15657059 (-) | *4930572O03Rik,* RIKEN cDNA 4930572O03 gene |
| 3.91 cM | 11918043-11922821 (+) | *4933402N22Rik,* RIKEN cDNA 4933402N22 gene |
| 3.69 cM | 9266118-9481825 (+) | *9330182L06Rik,* RIKEN cDNA 9330182L06 gene |
| 3.07 cM | 5579278-5664239 (-) | *A330021E22Rik,* RIKEN cDNA A330021E22 gene |
| 3.43 cM | 8660077-8748575 (+) | *Abcb1a,* ATP-binding cassette, sub-family B (MDR/TAP), member 1A |
| 3.43 cM | 8798147-8866314 (+) | *Abcb1b,* ATP-binding cassette, sub-family B (MDR/TAP), member 1B |
| 3.43 cM | 8893721-8959225 (+) | *Abcb4,* ATP-binding cassette, sub-family B (MDR/TAP), member 4 |
| 3.39 cM | 8072352-8368160 (-) | *Adam22,* a disintegrin and metallopeptidase domain 22 |
| 2.26 cM | 3928054-4080209 (+) | *Akap9,* A kinase (PRKA) anchor protein (yotiao) 9 |
| 2.26 cM | 3690000-3803109 (-) | *Ankib1,* ankyrin repeat and IBR domain containing 1 |
| 6.56 cM | 15934691-16374511 (+) | *Cacna2d1,* calcium channel, voltage-dependent, alpha2/delta subunit 1 |
| 9.83 cM | 21292961-21424677 (-) | *Ccdc146,* coiled-coil domain containing 146 |
| 8.11 cM | 17781690-17888959 (-) | *Cd36,* CD36 antigen |
| 2.61 cM | 4803391-5420312 (-) | *Cdk14,* cyclin-dependent kinase 14 |
| 2.04 cM | 3344312-3522225 (+) | *Cdk6,* cyclin-dependent kinase 6 |
| 2.98 cM | 5505109-5514958 (-) | *Cldn12,* claudin 12 |
| 3.5 cM | 8966033-8997324 (-) | *Crot,* carnitine O-octanoyltransferase |
| 2.3 cM | 4081145-4104746 (-) | *Cyp51,* cytochrome P450, family 51 |
| 3.43 cM | 8396973-8422716 (-) | *Dbf4,* DBF4 homolog (S. cerevisiae) |
| 3.69 cM | 9118801-9161749 (-) | *Dmtf1,* cyclin D binding myb-like transcription factor 1 |
| 2.22 cM | 3543844-3569810 (+) | *Fam133b*, family with sequence similarity 133, member B |
| 9.83 cM | 21372642-21378374 (+) | *Fgl2,* fibrinogen-like protein 2 |
| 2.61 cM | 4753873-4758035 (-) | *Fzd1,* frizzled homolog 1 (Drosophila) |
| 2.26 cM | 3632932-3647934 (-) | *Gatad1,* GATA zinc finger domain containing 1 |
| 6.48 cM | 14974113-14978935 (-) | *Gm10354,* predicted gene 10354 |
| 3.7 cM | 10236829-10237461 (+) | *Gm10482,* predicted gene 10482 |
| 6.49 cM | 15028950-15032998 (-) | *Gm17019,* predicted gene 17019 |
| 2.15 cM | 3473071-3473178 (-) | *Gm17590,* predicted gene, 17590 |
| syntenic | 15577337-15582097 (+) | *Gm21083,* predicted gene, 21083 |
| syntenic | 15471698-15476500 (-) | *Gm21149,* predicted gene, 21149 |
| syntenic | 15524731-15605059 (-) | *Gm21190,* predicted gene, 21190 |
| syntenic | 8179636-8181375 (+) | *Gm21759*, predicted gene, 21759 |
| syntenic | 15516489-15656679 (+) | *Gm21847,* predicted gene, 21847 |
| 7.96 cM | 17373208-17377788 (+) | *Gm3495,* predicted gene 3495 |
| 3.7 cM | 10242041-10246492 (-) | *Gm5152,* predicted gene 5152 |
| 3.82 cM | 11183072-11187810 (+) | *Gm5861,* predicted gene 5861 |
| 3.78 cM | 10866072-10870816 (+) | *Gm6455,* predicted gene 6455 |
| 3.87 cM | 11594956-11599784 (+) | *Gm6460,* predicted gene 6460 |
| 3.91 cM | 11845539-11850352 (+) | *Gm6465,* predicted gene 6465 |
| 3.79 cM | 10947730-10952590 (+) | *Gm8857,* predicted gene 8857 |
| 3.8 cM | 11040115-11044948 (+) | *Gm8871,* predicted pseudogene 8871 |
| 3.82 cM | 11127634-11132487 (+) | *Gm8879,* predicted gene 8879 |
| 3.83 cM | 11255933-11260789 (+) | *Gm8890,* predicted gene 8890 |
| 3.85 cM | 11416418-11421235 (+) | *Gm8897,* predicted gene 8897 |
| 3.86 cM | 11502685-11507417 (+) | *Gm8906,* predicted gene 8906 |
| 3.89 cM | 11682002-11686856 (+) | *Gm8922,* predicted gene 8922 |
| 3.9 cM | 11770548-11775300 (+) | *Gm8926,* predicted gene 8926 |
| 6.47 cM | 14910122-14914899 (-) | *Gm9758,* predicted gene 9758 |
| 2.34 cM | 4192367-4197651 (+) | *Gm9897,* predicted gene 9897 |
| 8.16 cM | 18265135-18360413 (-) | *Gnai1,* guanine nucleotide binding protein (G protein), alpha inhibiting 1 |
| 8.14 cM | 17962570-18019668 (+) | *Gnat3,* guanine nucleotide binding protein, alpha transducing 3 |
| 3.69 cM | 9485236-9725352 (-) | *Grm3,* glutamate receptor, metabotropic 3 |
| 3.02 cM | 5537454-5559538 (-) | *Gtpbp10,* GTP-binding protein 10 (putative) |
| 7.07 cM | 16553550-16619439 (+) | *Hgf,* hepatocyte growth factor |
| 2.26 cM | 3803165-3844515 (+) | *Krit1,* ankyrin repeat containing |
| 2.26 cM | 3845173-3866596 (+) | *Lrrd1,* leucine rich repeats and death domain containing 1 |
| 8.6 cM | 19227046-20704798 (+) | *Magi2,* membrane associated guanylate kinase, WW and PDZ domain containing 2 |
| 2.26 cM | 3890581-3893933 (-) | *Mterf,* mitochondrial transcription termination factor |
| 6.27 cM | 14514918-14863457 (+) | *Pclo,* piccolo (presynaptic cytomatrix protein) |
| 2.26 cM | 3596066-3637232 (+) | *Pex1,* peroxisomal biogenesis factor 1 |
| 9.83 cM | 20758663-20882124 (-) | *Phtf2,* putative homeodomain transcription factor 2 |
| 9.83 cM | 21186267-21291701 (+) | *Pion,* pigeon homolog (Drosophila) |
| 9.83 cM | 20986645-21055911 (-) | *Ptpn12,* protein tyrosine phosphatase, non-receptor type 12 |
| 2.25 cM | 3583978-3596585 (-) | *Rbm48,* RNA binding motif protein 48 |
| 9.83 cM | 21119172-21119522 (-) | *Rpl31-ps21,* ribosomal protein L31, pseudogene 21 |
| 9.83 cM | 20893028-20951822 (-) | *Rsbn1l,* round spermatid basic protein 1-like |
| 3.43 cM | 8490334-8622952 (-) | *Rundc3b,* RUN domain containing 3B |
| 4.31 cM | 13396784-13602565 (+) | *Sema3a,* sema domain, immunoglobulin domain (Ig), short basic domain, secreted, (semaphorin) 3A |
| 7.99 cM | 17574281-17730268 (+) | *Sema3c,* sema domain, immunoglobulin domain (Ig), short basic domain, secreted, (semaphorin) 3C |
| 3.97 cM | 12383166-12588943 (+) | *Sema3d,* sema domain, immunoglobulin domain (Ig), short basic domain, secreted, (semaphorin) 3D |
| 5.66 cM | 14025276-14256689 (+) | *Sema3e,* sema domain, immunoglobulin domain (Ig), short basic domain, secreted, (semaphorin) 3E |
| 3.43 cM | 8422850-8454790 (+) | *Slc25a40,* solute carrier family 25, member 40 |
| 5.26 cM | 13791619-13796820 (+) | *Speer3,* spermatogenesis associated glutamate (E)-rich protein 3 |
| 6.52 cM | 15709501-15714236 (-) | *Speer4c,* spermatogenesis associated glutamate (E)-rich protein 4c |
| 6.5 cM | 15619064-15623864 (+) | *Speer4d,* spermatogenesis associated glutamate (E)-rich protein 4d |
| 6.47 cM | 14933221-14938429 (-) | *Speer4e,* spermatogenesis associated glutamate (E)-rich protein 4e |
| 7.98 cM | 17476098-17480936 (+) | *Speer4f,* spermatogenesis associated glutamate (E)-rich protein 4f |
| 3.38 cM | 8046078-8069379 (+) | *Sri,* sorcin |
| 3.2 cM | 5736317-5749326 (-) | *Steap1,* six transmembrane epithelial antigen of the prostate 1 |
| 3.18 cM | 5665954-5694578 (-) | *Steap2,* six transmembrane epithelial antigen of prostate 2 |
| 3.37 cM | 7960472-7982213 (+) | *Steap4,* STEAP family member 4 |
| 3.69 cM | 9100737-9118983 (+) | *Tmem243,* transmembrane protein 243, mitochondrial |
| 9.83 cM | 20882191-20886870 (+) | *Tmem60,* transmembrane protein 60 |
| 1.77 cM | 3000101-3001111 (-) | *V1rg10,* vomeronasal 1 receptor, G10 |
| 3.2 cM | 6769030-6876523 (-) | *Zfp804b,* zinc finger protein 804B |

Genomic coordinates of genes were determined from the Mouse Genome Database (MGD), 2012. Eppig JT, *et al.* Nucleic Acids Res 2012; 40:D881-886.
