# Supplementary material for: Genetic Dissection of Quantitative Trait Loci for Hemostasis and Thrombosis on Mouse Chromosomes 11 and 5 Using Congenic and Subcongenic Strains
Source: PLoS One. 2013 Oct 17;8(10):e77539. doi: 10.1371/journal.pone.0077539 (PMC3798288; doi:10.1371/journal.pone.0077539)
Supplement: Table S9 — Candidate gene, Emilin1. (DOCX) [file pone.0077539.s009.docx]

| **Table S9. Candidtate gene, Emilin1 Deficient Mice.** | | | | | |
| --- | --- | --- | --- | --- | --- |
|  | **Tail Bleeding/Clot Stability Assay** | | **FeCl_3_ Induced Carotid Injury** | **CaCl_2_ Induced AAA Formation** | |
|  | Tail-BleedingTime (sec) | Clot StabilityTime (sec) | Occlusion Time (sec) | Change Diameter mm | |
|  |  |  |  | **8 wk age** | **20 wk age** |
| Wild-type | 125±26 | 211±37 | 11.8±1.2 | 0.16±0.32 | 0.07±0.03 |
| Emilin1-/- | 148±35 | 190±51 | 11.9±1.2 | 0.19±0.02 | 0.08±0.02 |

Emilin1+/- mice in C57BL/6J background obtained from G. Bressan, rederived at Jackson

Laboratory, and maintained with homozygous breeding pair. Wild-type mice = C57BL/6J. Values

are the mean±SEM, n=8-28. No significant differences between mouse strains. P > 0.05.
