# Supplementary material for: Genetic Dissection of Quantitative Trait Loci for Hemostasis and Thrombosis on Mouse Chromosomes 11 and 5 Using Congenic and Subcongenic Strains
Source: PLoS One. 2013 Oct 17;8(10):e77539. doi: 10.1371/journal.pone.0077539 (PMC3798288; doi:10.1371/journal.pone.0077539)
Supplement: Supplementary file 9 [file pone.0077539.s003.docx]

Table S3. Protein-coding Genes

Hmtb 6, Chromosome 11, 100-122 Mbp

cM	Genome Coodinates (Mbp)			Symbol	Name
63.35	100011195	100016212	-	*Krt33a*	keratin 33A
63.37	100023634	100029868	-	*Krt33b*	keratin 33B
63.38	100037347	100041554	-	*Krt34*	keratin 34
63.39	100046646	100050551	-	*Krt31*	keratin 31
63.4	100080848	100088226	-	*Krt32*	keratin 32
63.4	100092193	100096241	-	*Krt35*	keratin 35
63.41	100102007	100105626	-	*Krt36*	keratin 36
63.41	100117327	100121566	-	*Krt13*	keratin 13
63.41	100131758	100135928	-	*Krt15*	keratin 15
63.42	100140810	100148665	-	*Krt19*	keratin 19
63.43	100186781	100193246	-	*Krt9*	keratin 9
63.43	100203162	100207548	-	*Krt14*	keratin 14
63.44	100246091	100248902	-	*Krt16*	keratin 16
63.44	100256217	100261029	-	*Krt17*	keratin 17
63.44	100262882	100269871	-	*Krt42*	keratin 42
63.46	100319885	100322096	+	*Eif1*	eukaryotic translation initiation factor 1
63.46	100334407	100336996	+	*Gast*	gastrin
63.47	100347327	100356128	-	*Hap1*	huntingtin-associated protein 1
63.47	100368958	100397763	-	*Jup*	junction plakoglobin
63.47	100408456	100414836	-	*Leprel4*	leprecan-like 4
63.47	100415697	100424824	+	*Fkbp10*	FK506 binding protein 10
63.47	100422321	100441808	-	*Nt5c3b*	5'-nucleotidase, cytosolic IIIB
63.47	100441917	100457022	-	*Klhl10*	kelch-like 10
63.47	100462614	100472741	-	*Klhl11*	kelch-like 11
63.47	100476353	100528000	-	*Acly*	ATP citrate lyase
63.47	100545607	100572568	+	*Ttc25*	tetratricopeptide repeat domain 25
63.47	100574904	100591729	+	*Cnp*	2',3'-cyclic nucleotide 3' phosphodiesterase
63.47	100582818	100620168	-	*Dnajc7*	DnaJ (Hsp40) homolog, subfamily C, member 7
63.47	100619244	100627607	+	*Nkiras2*	NFKB inhibitor interacting Ras-like protein 2
63.47	100627543	100692455	-	*Zfp385c*	zinc finger protein 385C
63.52	100694884	100704271	-	*Dhx58*	DEXH (Asp-Glu-X-His) box polypeptide 58
63.53	100704746	100712465	-	*Kat2a*	K(lysine) acetyltransferase 2A
63.54	100713922	100714573	+	*Hspb9*	heat shock protein, alpha-crystallin-related, B9
63.54	100715009	100738215	-	*Rab5c*	RAB5C, member RAS oncogene family
63.57	100740376	100759942	-	*Kcnh4*	potassium voltage-gated channel, subfamily H (eag-related), member 4
63.6	100761069	100762931	-	*Hcrt*	hypocretin
63.6	100766032	100770957	-	*Ghdc*	GH3 domain containing
63.63	100780731	100850724	-	*Stat5b*	signal transducer and activator of transcription 5B
63.77	100859351	100885169	+	*Stat5a*	signal transducer and activator of transcription 5A
63.82	100885098	100939540	-	*Stat3*	signal transducer and activator of transcription 3
63.95	100956733	100970887	-	*Ptrf*	polymerase I and transcript release factor
64.04	101009452	101063719	+	*Atp6v0a1*	ATPase, H+ transporting, lysosomal V0 subunit A1
64.15	101070012	101077672	+	*Naglu*	alpha-N-acetylglucosaminidase (Sanfilippo disease IIIB)
64.17	101078411	101080527	+	*Hsd17b1*	hydroxysteroid (17-beta) dehydrogenase 1
64.17	101082565	101086619	+	*Coasy*	Coenzyme A synthase
64.18	101087277	101092207	+	*Mlx*	MAX-like protein X
64.19	101091823	101095436	-	*Psmc3ip*	proteasome (prosome, macropain) 26S subunit, ATPase 3, interacting protein
64.2	101096322	101119893	-	*Fam134c*	family with sequence similarity 134, member C
64.24	101119938	101126419	+	*Tubg1*	tubulin, gamma 1
64.3	101155907	101161787	+	*Tubg2*	tubulin, gamma 2
64.32	101162679	101171351	-	*Plekhh3*	pleckstrin homology domain containing, family H (with MyTH4 domain) member 3
64.33	101170523	101190724	+	*Cntnap1*	contactin associated protein-like 1
64.34	101172997	101175443	-	*Ccr10*	chemokine (C-C motif) receptor 10
64.37	101191115	101226463	-	*Ezh1*	enhancer of zeste homolog 1 (Drosophila)
64.47	101246028	101259546	+	*Ramp2*	receptor (calcitonin) activity modifying protein 2

64.48	101253707	101259549	+	*Vps25*	vacuolar protein sorting 25 (yeast)
64.51	101260567	101277409	+	*Wnk4*	WNK lysine deficient protein kinase 4
64.56	101277968	101279114	-	*Coa3*	cytochrome C oxidase assembly factor 3
64.56	101279012	101296317	+	*Cntd1*	cyclin N-terminal domain containing 1
64.58	101285952	101302286	-	*Becn1*	beclin 1, autophagy related
64.67	101316213	101323537	+	*Psme3*	proteaseome (prosome, macropain) activator subunit 3 (PA28 gamma, Ki)
64.7	101325063	101329702	+	*Aoc2*	amine oxidase, copper containing 2 (retina-specific)
64.72	101330605	101341938	+	*Aoc3*	amine oxidase, copper containing 3
64.83	101367561	101377903	+	*G6pc*	glucose-6-phosphatase, catalytic
64.95	101406839	101417615	-	*Aarsd1*	alanyl-tRNA synthetase domain containing 1
64.98	101418814	101425333	-	*Ptges3l*	prostaglandin E synthase 3 (cytosolic)-like
65.0	101425085	101435673	+	*Rundc1*	RUN domain containing 1
65.05	101442298	101445529	+	*Rpl27*	ribosomal protein L27
65.07	101448407	101458698	+	*Ifi35*	interferon-induced protein 35
65.1	101458745	101466230	-	*Vat1*	vesicle amine transport protein 1 homolog (T californica)
65.12	101464999	101471853	+	*Rnd2*	Rho family GTPase 2
65.18	101488764	101551955	-	*Brca1*	breast cancer 1
65.36	101552149	101581951	+	*Nbr1*	neighbor of Brca1 gene 1
65.44	101582242	101591788	+	*Tmem106a*	transmembrane protein 106A
65.48	101627195	101636100	+	*Rdm1*	RAD52 motif 1
65.48	101665541	101667832	+	*Arl4d*	ADP-ribosylation factor-like 4D
65.48	101732919	101767358	+	*Dhx8*	DEAH (Asp-Glu-Ala-His) box polypeptide 8
65.48	101769747	101785371	-	*Etv4*	ets variant gene 4 (E1A enhancer binding protein, E1AF)
65.48	101877510	101894374	-	*Meox1*	mesenchyme homeobox 1
65.48	101962458	101967015	-	*Sost*	sclerostin
65.48	101971143	101987013	-	*Dusp3*	dual specificity phosphatase 3 (vaccinia virus phosphatase VH1-related)
65.48	101984279	101992264	+	*1700006E09Rik*	RIKEN cDNA 1700006E09 gene
65.48	101999652	102028461	-	*Mpp3*	membrane protein, palmitoylated 3 (MAGUK p55 subfamily member 3)
65.48	102041509	102055620	+	*Cd300lg*	CD300 antigen like family member G
65.48	102057015	102088515	-	*Mpp2*	membrane protein, palmitoylated 2 (MAGUK p55 subfamily member 2)
65.48	102099930	102101319	-	*Ppy*	pancreatic polypeptide
65.48	102106676	102107832	-	*Pyy*	peptide YY
65.48	102145513	102149477	+	*Nags*	N-acetylglutamate synthase
65.52	102152546	102156404	-	*Tmem101*	transmembrane protein 101
65.6	102162497	102185296	-	*Lsm12*	LSM12 homolog (S. cerevisiae)
65.79	102189620	102194081	+	*G6pc3*	glucose 6 phosphatase, catalytic, 3
65.82	102194432	102230166	-	*Hdac5*	histone deacetylase 5
66.21	102248882	102265187	+	*BC030867*	cDNA sequence BC030867
66.29	102268743	102279462	+	*Asb16*	ankyrin repeat and SOCS box-containing 16
66.29	102284931	102289237	+	*Tmub2*	transmembrane and ubiquitin-like domain containing 2
66.29	102289300	102296631	-	*Atxn7l3*	ataxin 7-like 3
66.29	102304560	102319742	-	*Ubtf*	upstream binding transcription factor, RNA polymerase I
66.29	102348824	102366203	-	*Slc4a1*	solute carrier family 4 (anion exchanger), member 1
66.29	102393403	102402555	+	*Rundc3a*	RUN domain containing 3A
66.29	102402985	102407946	-	*Slc25a39*	solute carrier family 25, member 39
66.29	102430315	102437048	+	*Grn*	granulin
66.29	102436981	102447682	-	*Fam171a2*	family with sequence similarity 171, member A2
66.29	102453297	102470122	-	*Itga2b*	integrin alpha 2b
66.31	102475915	102556392	-	*Gpatch8*	G patch domain containing 8
66.42	102576398	102579461	-	*Gm11627*	predicted gene 11627
66.45	102604396	102608058	+	*Fzd2*	frizzled homolog 2 (Drosophila)
66.48	102663716	102682237	+	*Gm1564*	predicted gene 1564
66.48	102684686	102697782	-	*Ccdc43*	coiled-coil domain containing 43
66.48	102761439	102780262	+	*Adam11*	a disintegrin and metallopeptidase domain 11
66.48	102799579	102819700	-	*Gjc1*	gap junction protein, gamma 1
66.48	102835849	102838040	+	*Higd1b*	HIG1 domain family, member 1B
66.48	102838473	102880985	-	*Eftud2*	elongation factor Tu GTP binding domain containing 2
66.48	102881204	102885216	+	*Ccdc103*	coiled-coil domain containing 103
66.48	102885169	102886731	+	*Fam187a*	family with sequence similarity 187, member A

66.48	102887336	102900912	-	*Gfap*	glial fibrillary acidic protein
66.48	102905529	102925124	-	*Kif18b*	kinesin family member 18B
66.48	102939264	102946688	-	*C1ql1*	complement component 1, q subcomponent-like 1
66.71	102994047	103028333	-	*Dcakd*	dephospho-CoA kinase domain containing
66.71	103028190	103068912	+	*Nmt1*	N-myristoyltransferase 1
66.71	103070304	103101658	-	*Plcd3*	phospholipase C, delta 3
66.71	103101682	103112200	+	*Acbd4*	acyl-Coenzyme A binding domain containing 4
66.71	103116231	103119725	+	*Hexim1*	hexamethylene bis-acetamide inducible 1
66.71	103132429	103139876	+	*Hexim2*	hexamethylene bis-acetamide inducible 2
66.71	103171107	103198901	+	*Fmnl1*	formin-like 1
66.75	103198946	103208548	-	*1700023F06Rik*	RIKEN cDNA 1700023F06 gene
66.77	103208127	103218432	-	*Spata32*	spermatogenesis associated 32
66.8	103219762	103267472	-	*Map3k14*	mitogen-activated protein kinase kinase kinase 14
67.02	103331497	103363692	-	*Arhgap27*	Rho GTPase activating protein 27
67.09	103365088	103412664	-	*Plekhm1*	pleckstrin homology domain containing, family M (with RUN domain) member 1
67.27	103451955	103504597	-	*Lrrc37a*	leucine rich repeat containing 37A
67.38	103534577	103621140	-	*Gm884*	predicted gene 884
67.4	103631070	103638890	-	*Lyzl6*	lysozyme-like 6
67.41	103649570	103650579	+	*Rprml*	reprimo-like
67.43	103676849	103697898	-	*Gosr2*	golgi SNAP receptor complex member 2
67.47	103727364	103749821	-	*Wnt9b*	wingless-type MMTV integration site 9B
67.5	103774150	103817957	+	*Wnt3*	wingless-related MMTV integration site 3
67.54	103821782	103954056	-	*Nsf*	N-ethylmaleimide sensitive fusion protein
67.58	103879915	103880243	-	*Gm8738*	predicted gene 8738
67.64	103966739	103985337	+	*Arf2*	ADP-ribosylation factor 2
67.77	104132855	104175523	+	*Crhr1*	corticotropin releasing hormone receptor 1
67.79	104186327	104188551	+	*Sppl2c*	signal peptide peptidase 2C
67.79	104231390	104332090	+	*Mapt*	microtubule-associated protein tau
67.79	104333501	104468861	-	*Kansl1*	KAT8 regulatory NSL complex subunit 1
67.79	104502745	104550620	-	*Cdc27*	cell division cycle 27
67.79	104550663	104595753	+	*Myl4*	myosin, light polypeptide 4
67.79	104573547	104573897	+	*Gm7020*	predicted gene 7020
67.84	104608000	104670476	+	*Itgb3*	integrin beta 3
67.95	104685707	104753962	+	*Gm11639*	predicted gene 11639
68.61	105063592	105117537	+	*Efcab3*	EF-hand calcium binding domain 3
68.61	105126425	105140394	+	*Mettl2*	methyltransferase like 2
68.61	105146893	105147261	+	*Gm10842*	predicted gene 10842
68.61	105178807	105283959	+	*Tlk2*	tousled-like kinase 2 (Arabidopsis)
68.73	105292643	105351139	+	*Mrc2*	mannose receptor, C type 2
68.73	105360798	105456735	-	*March10*	membrane-associated ring finger (C3HC4) 10
68.73	105589986	105929304	+	*Tanc2*	tetratricopeptide repeat, ankyrin repeat and coiled-coil containing 2
68.81	105933702	105953336	-	*Cyb561*	cytochrome b-561
68.84	105967945	105989964	+	*Ace*	angiotensin I converting enzyme (peptidyl-dipeptidase A) 1
68.86	105994675	106005443	+	*Ace3*	angiotensin I converting enzyme (peptidyl-dipeptidase A) 3
68.87	106008124	106034549	+	*Kcnh6*	potassium voltage-gated channel, subfamily H (eag-related), member 6
68.89	106036872	106059324	+	*Dcaf7*	DDB1 and CUL4 associated factor 7
68.89	106066061	106073612	+	*Taco1*	translational activator of mitochondrially encoded cytochrome c oxidase I
68.89	106084613	106155446	+	*Map3k3*	mitogen-activated protein kinase kinase kinase 3
68.89	106156256	106160860	-	*Limd2*	LIM domain containing 2
68.89	106160850	106161158	+	*Gm10840*	predicted gene 10840
68.89	106163330	106202168	-	*Strada*	STE20-related kinase adaptor alpha
68.89	106197408	106216344	-	*Ccdc47*	coiled-coil domain containing 47
68.89	106216926	106249139	+	*Ddx42*	DEAD (Asp-Glu-Ala-Asp) box polypeptide 42
68.89	106249142	106256079	-	*Ftsj3*	FtsJ homolog 3 (E. coli)
68.89	106256154	106263120	+	*Psmc5*	protease (prosome, macropain) 26S subunit, ATPase 5
68.89	106263179	106272972	-	*Smarcd2*	SWI/SNF related, matrix associated, actin dependent regulator of chromatin, subfamily d, member 2
68.89	106276672	106288745	+	*Tcam1*	testicular cell adhesion molecule 1

68.89	106300271	106301865	-	*Gh*	growth hormone
68.89	106311341	106314762	-	*Cd79b*	CD79B antigen
68.91	106318592	106353288	-	*Scn4a*	sodium channel, voltage-gated, type IV, alpha
69.05	106365474	106377114	+	*2310007L24Rik*	RIKEN cDNA 2310007L24 gene
69.09	106377656	106388075	-	*Icam2*	intercellular adhesion molecule 2
69.14	106394650	106487852	-	*Ern1*	endoplasmic reticulum (ER) to nucleus signalling 1
69.46	106502147	106613423	-	*Tex2*	testis expressed gene 2
69.92	106654217	106750628	-	*Pecam1*	platelet/endothelial cell adhesion molecule 1
70.0	106751226	106768794	+	*Milr1*	mast cell immunoglobulin like receptor 1
70.01	106768253	106779537	-	*Polg2*	polymerase (DNA directed), gamma 2, accessory subunit
70.01	106780355	106789185	-	*Ddx5*	DEAD (Asp-Glu-Ala-Asp) box polypeptide 5
70.02	106789275	106819930	+	*Cep95*	centrosomal protein 95
70.03	106820066	106920715	-	*Smurf2*	SMAD specific E3 ubiquitin protein ligase 2
70.08	106988629	106999541	-	*Kpna2*	karyopherin (importin) alpha 2
70.11	107033083	107132127	-	*Bptf*	bromodomain PHD finger transcription factor
70.19	107166663	107189381	-	*Nol11*	nucleolar protein 11
70.21	107207892	107470699	-	*Pitpnc1*	phosphatidylinositol transfer protein, cytoplasmic 1
70.29	107479484	107504362	+	*Psmd12*	proteasome (prosome, macropain) 26S subunit, non-ATPase, 12
70.29	107547930	107693826	+	*Helz*	helicase with zinc finger domain
70.29	107703218	107716522	-	*Cacng1*	calcium channel, voltage-dependent, gamma subunit 1
70.29	107732357	107794557	-	*Cacng4*	calcium channel, voltage-dependent, gamma subunit 4
70.44	107874605	107915055	-	*Cacng5*	calcium channel, voltage-dependent, gamma subunit 5
70.8	107933387	108343928	-	*Prkca*	protein kinase C, alpha
71.8	108343354	108414396	+	*Apoh*	apolipoprotein H
71.83	108425192	108860615	+	*Cep112*	centrosomal protein 112
71.83	108920349	108950783	+	*Axin2*	axin2
71.84	109139291	109144369	-	*E030025P04Rik*	RIKEN cDNA E030025P04 gene
71.86	109225355	109298129	-	*Rgs9*	regulator of G-protein signaling 9
71.88	109354778	109363654	-	*Gm11696*	predicted gene 11696
71.88	109362831	109401369	+	*Gna13*	guanine nucleotide binding protein, alpha 13
71.88	109413917	109420405	+	*9930022D16Rik*	RIKEN cDNA 9930022D16 gene
71.89	109425976	109452813	+	*Amz2*	archaelysin family metallopeptidase 2
71.94	109450855	109473598	-	*Slc16a6*	solute carrier family 16 (monocarboxylic acid transporters), member 6
71.98	109473374	109573330	+	*Arsg*	arylsulfatase G
72.18	109573331	109611967	-	*Wipi1*	WD repeat domain, phosphoinositide interacting 1
72.33	109649405	109669656	+	*Prkar1a*	protein kinase, cAMP dependent regulatory, type I, alpha
72.37	109669749	109722279	-	*Fam20a*	family with sequence similarity 20, member A
72.61	109791557	109828022	-	*1700012B07Rik*	RIKEN cDNA 1700012B07 gene
72.88	109932190	109995845	-	*Abca8b*	ATP-binding cassette, sub-family A (ABC1), member 8b
73.07	110025634	110095978	-	*Abca8a*	ATP-binding cassette, sub-family A (ABC1), member 8a
73.22	110100749	110168196	-	*Abca9*	ATP-binding cassette, sub-family A (ABC1), member 9
73.37	110176820	110251776	-	*Abca6*	ATP-binding cassette, sub-family A (ABC1), member 6
73.53	110269369	110337716	-	*Abca5*	ATP-binding cassette, sub-family A (ABC1), member 5
73.6	110399122	110525522	+	*Map2k6*	mitogen-activated protein kinase kinase 6
75.01	110968033	111027968	+	*Kcnj16*	potassium inwardly-rectifying channel, subfamily J, member 16
75.23	111066164	111076821	+	*Kcnj2*	potassium inwardly-rectifying channel, subfamily J, member 2
75.93	112431190	112431636	-	*Gm11678*	predicted gene 11678
77.27	112782224	112787760	+	*Sox9*	SRY-box containing gene 9
78.74	113244853	113650079	-	*Slc39a11*	solute carrier family 39 (metal ion transporter), member 11
79.05	113619342	113626028	+	*Sstr2*	somatostatin receptor 2
79.1	113649169	113667054	+	*Cog1*	component of oligomeric golgi complex 1
79.12	113661319	113684155	-	*Fam104a*	family with sequence similarity 104, member A
79.15	113684412	113694647	+	*D11Wsu47e*	DNA segment, Chr 11, Wayne State University 47, expressed
79.17	113698172	113710017	-	*Cpsf4l*	cleavage and polyadenylation specific factor 4-like
79.22	113726850	113751881	-	*Cdc42ep4*	CDC42 effector protein (Rho GTPase binding) 4
79.3	113776374	114067046	-	*Sdk2*	sidekick homolog 2 (chicken)
79.82	114668524	114672331	+	*Rpl38*	ribosomal protein L38
79.85	114675431	114720977	+	*Ttyh2*	tweety homolog 2 (Drosophila)
80.19	114727408	114757889	+	*Dnaic2*	dynein, axonemal, intermediate chain 2

80.44	114765388	114790739	+	*Kif19a*	kinesin family member 19A
80.54	114791217	114795945	-	*Btbd17*	BTB (POZ) domain containing 17
80.54	114798924	114806745	+	*Gpr142*	G protein-coupled receptor 142
80.54	114851152	114872617	+	*Gprc5c*	G protein-coupled receptor, family C, group 5, member C
80.54	114890041	114904654	+	*Cd300a*	CD300A antigen
80.55	114922781	114934386	-	*Cd300lb*	CD300 antigen like family member B
80.56	114956116	114969157	-	*Cd300c*	CD300C antigen
80.56	114982274	114989922	-	*Cd300ld*	CD300 molecule-like family member d
80.57	114996721	115001880	-	*AF251705*	cDNA sequence AF251705
80.57	115010335	115016193	-	*Gm11709*	predicted gene 11709
80.57	115020728	115027012	-	*Gm11710*	predicted gene 11710
80.57	115031486	115037769	-	*Gm11711*	predicted gene 11711
80.58	115042131	115048409	-	*Cd300lh*	CD300 antigen like family member H
80.58	115051917	115062177	-	*Cd300e*	CD300e antigen
80.59	115091431	115162236	+	*Rab37*	RAB37, member of RAS oncogene family
80.59	115116214	115133992	-	*Cd300lf*	CD300 antigen like family member F
80.65	115163341	115181181	+	*Slc9a3r1*	solute carrier family 9 (sodium/hydrogen exchanger), member 3 regulator 1
80.68	115182832	115187859	-	*Nat9*	N-acetyltransferase 9 (GCN5-related, putative)
80.69	115187487	115247023	+	*Tmem104*	transmembrane protein 104
80.8	115249169	115265429	-	*Grin2c*	glutamate receptor, ionotropic, NMDA2C (epsilon 3)
80.84	115268024	115277050	-	*Fdxr*	ferredoxin reductase
80.84	115279622	115297663	-	*Fads6*	fatty acid desaturase domain family, member 6
80.84	115307163	115332303	+	*Otop2*	otopetrin 2
80.84	115315192	115322041	-	*Ush1g*	Usher syndrome 1G
80.84	115334731	115346927	+	*Otop3*	otopetrin 3
80.84	115347707	115367756	-	*Hid1*	HID1 domain containing
80.84	115381916	115396132	+	*Cdr2l*	cerebellar degeneration-related protein 2-like
80.84	115403752	115412674	+	*Ict1*	immature colon carcinoma transcript 1
80.84	115415689	115419962	-	*Atp5h*	ATP synthase, H+ transporting, mitochondrial F0 complex, subunit d
80.84	115420128	115431274	+	*Kctd2*	potassium channel tetramerisation domain containing 2
80.84	115440545	115448270	+	*4933422H20Rik*	RIKEN cDNA 4933422H20 gene
80.84	115462474	115474398	+	*Slc16a5*	solute carrier family 16 (monocarboxylic acid transporters), member 5
80.84	115475667	115490467	+	*Armc7*	armadillo repeat containing 7
80.84	115490420	115491862	-	*Nt5c*	5',3'-nucleotidase, cytosolic
80.84	115497353	115514387	-	*Hn1*	hematological and neurological expressed sequence 1
80.84	115523102	115536276	-	*Sumo2*	SMT3 suppressor of mif two 3 homolog 2 (yeast)
80.87	115564434	115583985	+	*Nup85*	nucleoporin 85
80.89	115584255	115604051	-	*Gga3*	golgi associated, gamma adaptin ear containing, ARF binding protein 3
80.9	115603925	115608036	+	*Mrps7*	mitchondrial ribosomal protein S7
80.9	115607918	115612969	-	*Mif4gd*	MIF4G domain containing
80.91	115614178	115628281	-	*Slc25a19*	solute carrier family 25 (mitochondrial thiamine pyrophosphate carrier), member 19
80.91	115644045	115708597	-	*Grb2*	growth factor receptor bound protein 2
80.91	115765433	115799366	+	*2310067B10Rik*	RIKEN cDNA 2310067B10 gene
80.91	115799183	115813639	-	*Caskin2*	CASK-interacting protein 2
80.91	115814724	115823094	+	*Tsen54*	tRNA splicing endonuclease 54 homolog (S. cerevisiae)
80.91	115824049	115855780	+	*Llgl2*	lethal giant larvae homolog 2 (Drosophila)
80.91	115861612	115892592	+	*Myo15b*	myosin XVB
80.91	115892595	115933477	-	*Recql5*	RecQ protein-like 5
80.91	115899966	115906269	+	*Smim5*	small integral membrane protein 5
80.91	115912017	115913920	+	*Smim6*	small integral membrane protein 6
80.91	115933282	115966725	+	*Sap30bp*	SAP30 binding protein
80.91	115974709	116008412	+	*Itgb4*	integrin beta 4
80.91	116008457	116012719	-	*Galk1*	galactokinase 1
80.91	116021912	116027962	-	*H3f3b*	H3 histone, family 3B
80.91	116030322	116061214	+	*Unk*	unkempt homolog (Drosophila)

80.91	116062095	116077961	-	*Unc13d*	unc-13 homolog D (C. elegans)
80.91	116078573	116086995	-	*Wbp2*	WW domain binding protein 2
80.91	116105752	116127210	-	*Trim47*	tripartite motif-containing 47
80.91	116121846	116131128	-	*Trim65*	tripartite motif-containing 65
80.91	116131817	116138868	-	*Mrpl38*	mitochondrial ribosomal protein L38
80.93	116142285	116168166	-	*Fbf1*	Fas (TNFRSF6) binding factor 1
80.96	116171888	116199045	-	*Acox1*	acyl-Coenzyme A oxidase 1, palmitoyl
81.0	116198855	116215318	+	*Ten1*	TEN1 telomerase capping complex subunit
81.02	116220559	116238077	-	*Evpl*	envoplakin
81.06	116245166	116274217	-	*Srp68*	signal recognition particle 68
81.1	116280939	116283938	+	*Galr2*	galanin receptor 2
81.11	116288001	116307233	-	*Exoc7*	exocyst complex component 7
81.16	116330704	116335399	-	*Foxj1*	forkhead box J1
81.17	116336353	116413032	-	*Rnf157*	ring finger protein 157
81.29	116434094	116439077	+	*Ubald2*	UBA-like domain containing 2
81.3	116441325	116455237	-	*Qrich2*	glutamine rich 2
81.34	116470845	116494202	-	*Prpsap1*	phosphoribosyl pyrophosphate synthetase-associated protein 1
81.42	116530925	116536674	+	*Sphk1*	sphingosine kinase 1
81.42	116537740	116581447	-	*Ube2o*	ubiquitin-conjugating enzyme E2O
81.43	116586432	116597680	+	*Aanat*	arylalkylamine N-acetyltransferase
81.43	116598165	116627019	-	*Rhbdf2*	rhomboid 5 homolog 2 (Drosophila)
81.45	116645595	116654313	-	*Cygb*	cytoglobin
81.46	116653534	116668389	+	*Gm11744*	predicted gene 11744
81.49	116671660	116675799	+	*1810032O08Rik*	RIKEN cDNA 1810032O08 gene
81.49	116675303	116694868	-	*St6galnac2*	ST6 (alpha-N-acetyl-neuraminyl-2,3-beta-galactosyl-1,3) -N-acetylgalactosaminide alpha-2,6-sialyltransferase 2
81.49	116765025	116775507	-	*St6galnac1*	ST6 (alpha-N-acetyl-neuraminyl-2,3-beta-galactosyl-1,3) -N-acetylgalactosaminide alpha-2,6-sialyltransferase 1
81.49	116803062	116828046	-	*Mxra7*	matrix-remodelling associated 7
81.49	116837432	116843449	-	*Jmjd6*	jumonji domain containing 6
81.49	116843278	116854263	+	*Mettl23*	methyltransferase like 23
81.49	116849901	116853094	-	*Srsf2*	serine/arginine-rich splicing factor 2
81.49	116852440	116875635	+	*Mfsd11*	major facilitator superfamily domain containing 11
81.49	116918863	116986948	+	*Mgat5b*	mannoside acetylglucosaminyltransferase 5, isoenzyme B
81.99	117115168	117159268	+	*Sec14l1*	SEC14-like 1 (S. cerevisiae)
82.61	117199661	117362325	+	*Sept9*	septin 9
82.96	117484368	117489014	+	*Gm11733*	predicted gene 11733
82.96	117654573	117763439	+	*Tnrc6c*	trinucleotide repeat containing 6C
82.96	117765988	117782198	-	*Tmc6*	transmembrane channel-like gene family 6
82.96	117782076	117793110	+	*Tmc8*	transmembrane channel-like gene family 8
82.96	117797660	117807301	+	*6030468B19Rik*	RIKEN cDNA 6030468B19 gene
82.96	117809668	117839904	+	*Gm20708*	predicted gene 20708
82.96	117809668	117814283	+	*Syngr2*	synaptogyrin 2
82.96	117815526	117826092	-	*Tk1*	thymidine kinase 1
82.96	117825924	117839908	+	*Afmid*	arylformamidase
82.96	117849251	117855743	+	*Birc5*	baculoviral IAP repeat-containing 5
82.96	117860752	117865543	+	*Tmem235*	transmembrane protein 235
82.96	117867952	117873481	-	*Tha1*	threonine aldolase 1
82.96	117966079	117970047	-	*Socs3*	suppressor of cytokine signaling 3
82.96	117986292	118024011	+	*Pgs1*	phosphatidylglycerophosphate synthase 1
82.96	118023080	118125944	-	*Dnah17*	dynein, axonemal, heavy chain 17
82.96	118132019	118248560	-	*Cyth1*	cytohesin 1
83.06	118259651	118290231	-	*Usp36*	ubiquitin specific peptidase 36
83.09	118301069	118355740	-	*Timp2*	tissue inhibitor of metalloproteinase 2
83.09	118332360	118342500	-	*BC100451*	cDNA sequence BC100451
83.14	118392751	118402092	-	*Lgals3bp*	lectin, galactoside-binding, soluble, 3 binding protein
83.15	118406289	118419086	-	*Cant1*	calcium activated nucleotidase 1
83.17	118428203	118449963	+	*C1qtnf1*	C1q and tumor necrosis factor related protein 1
83.21	118476829	118489209	+	*Engase*	endo-beta-N-acetylglucosaminidase
83.22	118489762	118911597	-	*Rbfox3*	RNA binding protein, fox-1 homolog (C. elegans) 3

83.33	118988216	118992665	+	*Enpp7*	ectonucleotide pyrophosphatase/phosphodiesterase 7
83.33	119022962	119031270	+	*Cbx2*	chromobox 2
83.33	119036305	119040969	-	*Cbx8*	chromobox 8
83.34	119077573	119086221	-	*Cbx4*	chromobox 4
83.34	119143045	119228499	-	*Tbc1d16*	TBC1 domain family, member 16
83.35	119228572	119265236	+	*Ccdc40*	coiled-coil domain containing 40
83.35	119267887	119285454	+	*Gaa*	glucosidase, alpha, acid
83.35	119288363	119300089	-	*Eif4a3*	eukaryotic translation initiation factor 4A3
83.36	119307768	119345375	+	*Card14*	caspase recruitment domain family, member 14
83.36	119343425	119355536	-	*Sgsh*	N-sulfoglucosamine sulfohydrolase (sulfamidase)
83.36	119355557	119381079	+	*Slc26a11*	solute carrier family 26, member 11
83.48	119393100	119487418	+	*Rnf213*	ring finger protein 213
83.8	119491347	119511437	+	*Endov*	endonuclease V
83.95	119538719	119547753	-	*Nptx1*	neuronal pentraxin 1
83.96	119602905	119899576	+	*Rptor*	regulatory associated protein of MTOR, complex 1
83.96	119913441	119919548	+	*Chmp6*	charged multivesicular body protein 6
83.96	119942763	120006782	+	*Baiap2*	brain-specific angiogenesis inhibitor 1-associated protein 2
83.96	120007313	120047167	-	*Aatk*	apoptosis-associated tyrosine kinase
83.96	120064430	120086827	-	*Azi1*	5-azacytidine induced gene 1
83.96	120090531	120098731	-	*Enthd2*	ENTH domain containing 2
83.96	120098928	120100704	+	*1810043H04Rik*	RIKEN cDNA 1810043H04 gene
83.96	120103960	120151346	-	*Slc38a10*	solute carrier family 38, member 10
83.96	120232947	120292282	+	*Bahcc1*	BAH domain and coiled-coil containing 1
84.07	120345690	120348542	-	*Actg1*	actin, gamma, cytoplasmic 1
84.07	120348678	120351190	+	*0610009L18Rik*	RIKEN cDNA 0610009L18 gene
84.08	120361534	120368168	+	*Fscn2*	fascin homolog 2, actin-bundling protein, retinal (Strongylocentrotus purpuratus)
84.08	120369055	120378764	-	*2310003H01Rik*	RIKEN cDNA 2310003H01 gene
84.09	120380370	120437708	-	*Nploc4*	nuclear protein localization 4 homolog (S. cerevisiae)
84.13	120442644	120446950	+	*Tspan10*	tetraspanin 10
84.14	120447605	120453500	-	*Pde6g*	phosphodiesterase 6G, cGMP-specific, rod, gamma
84.15	120456606	120458068	-	*Oxld1*	oxidoreductase like domain containing 1
84.15	120458115	120464358	+	*Ccdc137*	coiled-coil domain containing 137
84.16	120464866	120467607	-	*Arl16*	ADP-ribosylation factor-like 16
84.16	120467635	120483979	+	*Hgs*	HGF-regulated tyrosine kinase substrate
84.18	120484613	120489065	+	*Mrpl12*	mitochondrial ribosomal protein L12
84.19	120491840	120499187	+	*Slc25a10*	solute carrier family 25 (mitochondrial carrier, dicarboxylate transporter), member 10
84.24	120530699	120538986	+	*Gcgr*	glucagon receptor
84.25	120542888	120549727	-	*Fam195b*	family with sequence similarity 195, member B
84.26	120549979	120551132	-	*Ppp1r27*	protein phosphatase 1, regulatory subunit 27
84.27	120560298	120573253	-	*P4hb*	prolyl 4-hydroxylase, beta polypeptide
84.29	120578104	120581624	-	*Arhgdia*	Rho GDP dissociation inhibitor (GDI) alpha
84.31	120592121	120598365	-	*Alyref*	Aly/REF export factor
84.32	120598421	120608198	+	*Anapc11*	anaphase promoting complex subunit 11
84.33	120608477	120609093	+	*Npb*	neuropeptide B
84.33	120610087	120617936	-	*Pcyt2*	phosphate cytidylyltransferase 2, ethanolamine
84.34	120618372	120625240	-	*Sirt7*	sirtuin 7
84.35	120625117	120633600	-	*Mafg*	v-maf musculoaponeurotic fibrosarcoma oncogene family, protein G (avian)
84.37	120639416	120643769	-	*Pycr1*	pyrroline-5-carboxylate reductase 1
84.37	120646031	120648158	-	*Myadml2*	myeloid-associated differentiation marker-like 2
84.38	120653788	120661175	-	*Notum*	notum pectinacetylesterase homolog (Drosophila)
84.41	120672973	120709447	+	*Aspscr1*	alveolar soft part sarcoma chromosome region, candidate 1 (human)
84.45	120710942	120713738	-	*Stra13*	stimulated by retinoic acid 13
84.45	120713925	120721128	+	*Lrrc45*	leucine rich repeat containing 45
84.46	120721470	120723969	+	*Rac3*	RAS-related C3 botulinum substrate 3
84.47	120725399	120727281	-	*Dcxr*	dicarbonyl L-xylulose reductase
84.47	120729489	120732114	-	*Cbr2*	carbonyl reductase 2

84.51	120762794	120764419	+	*Hmga1-rs1*	high mobility group AT-hook I, related sequence 1
84.53	120780746	120784207	-	*Rfng*	RFNG O-fucosylpeptide 3-beta-N-acetylglucosaminyltransferase
84.54	120784272	120789102	+	*Gps1*	G protein pathway suppressor 1
84.54	120789201	120796403	-	*Dus1l*	dihydrouridine synthase 1-like (S. cerevisiae)
84.56	120805940	120824178	-	*Fasn*	fatty acid synthase
84.59	120826529	120932872	-	*Ccdc57*	coiled-coil domain containing 57
84.73	120948480	120958775	+	*Slc16a3*	solute carrier family 16 (monocarboxylic acid transporters), member 3
84.75	120961749	120991330	-	*Csnk1d*	casein kinase 1, delta
84.85	121036747	121039418	-	*Cd7*	CD7 antigen
84.87	121053458	121063569	-	*Sectm1b*	secreted and transmembrane 1B
84.89	121067407	121081220	-	*Sectm1a*	secreted and transmembrane 1A
84.96	121116215	121118677	-	*Tex19.2*	testis expressed gene 19.2
85.0	121146143	121148319	+	*Tex19.1*	testis expressed gene 19.1
85.02	121160271	121161973	+	*Uts2r*	urotensin 2 receptor
85.04	121177591	121204711	-	*Ogfod3*	2-oxoglutarate and iron-dependent oxygenase domain containing 3
85.08	121204435	121222647	+	*Hexdc*	hexosaminidase (glycosyl hydrolase family 20, catalytic domain) containing
85.1	121222588	121229322	-	*BC017643*	cDNA sequence BC017643
85.12	121237253	121255856	+	*Narf*	nuclear prelamin A recognition factor
85.15	121259990	121309896	+	*Foxk2*	forkhead box K2
85.24	121327224	121354445	-	*Wdr45b*	WD repeat domain 45B
85.28	121356125	121388251	-	*Rab40b*	Rab40b, member RAS oncogene family
85.37	121421401	121431288	+	*Fn3krp*	fructosamine 3 kinase related protein
85.38	121434913	121450491	+	*Fn3k*	fructosamine 3 kinase
85.41	121451949	121617164	+	*Tbcd*	tubulin-specific chaperone d
85.49	121510978	121519333	-	*Zfp750*	zinc finger protein 750
86.14	121616197	121673153	-	*B3gntl1*	UDP-GlcNAc:betaGal beta-1,3-N-acetylglucosaminyltransferase -like 1
86.89	121701544	121716306	+	*Metrnl*	meteorin, glial cell differentiation regulator-like
87.9	121830247	121843423	+	*Ptchd3*	patched domain containing 3
87.95	121905775	121925329	-	*Gm12588*	predicted gene 12588
87.98	121942410	121956727	-	*Gm9631*	predicted gene 9631
syntenic	102999108	103000650		*Gm9778*	predicted gene 9778
syntenic	105943703	105945252		*Gm9910*	predicted gene 9910

Genomic coordinates of genes were determined from the mouse.
Genome Database (MGD), 2012. Eppig JT et al. Nucleaic Acids Res 2012; 40:D881-886.
